# Predictors of significant tricuspid regurgitation in atrial fibrillation: a meta-analysis

**DOI:** 10.3389/fcvm.2025.1428964

**Published:** 2025-03-06

**Authors:** Xiuxiu Zhang, Na Zhang, Jia Fu, Dapeng Yu

**Affiliations:** Department of Cardiovascular Surgery, Dong E Hospital, Dong’e, China

**Keywords:** significant tricuspid regurgitation, atrial fibrillation, echocardiography parameters, predictors, meta-analysis

## Abstract

**Aims:**

Significant tricuspid regurgitation (TR) in atrial fibrillation (AF) patients is becoming a global issue, as it can lead to progressive right ventricular enlargement and heart failure, thereby increasing morbidity and mortality. This study aimed to evaluate potential predictors of significant TR in AF patients using open databases.

**Methods:**

PubMed, EMBASE, the Cochrane Library, and Web of Science were searched for relevant studies from inception to September 2023. Using STATA 14.0 statistical software, hazard ratios (HRs) were calculated for data synthesis. The potential predictors included clinical characteristics, echocardiography parameters, and prior comorbidities. Evidence certainty was evaluated based on the GRADE system.

**Results:**

In total, 12 studies involving almost 16,000 patients were included in this review. Female sex (HR = 2.14; 95% CI: 1.84–2.49; I^2^ = 0.0%; *p* = 0.430), persistent atrial fibrillation (HR = 2.99; 95% CI: 2.47–3.61; I^2^ = 0.0%; *p* = 0.896), left ventricular ejection fraction [standard mean difference (SMD) = −0.16; 95% CI:−0.30 to −0.03; I^2^ = 69.8%; *p* < 0.000], age (HR = 1.07; 95% CI: 1.04–1.09; I^2^ = 72.3%; *p* = 0.013), heart failure (HR = 1.86; 95% CI: 1.45–2.39; I^2^ = 9.0%; *p* = 0.348), age ≥65 years (HR = 2.30; 95% CI: 1.63–3.25; I^2^ = 55.1%; *p* = 0.108), chronic lung disease (HR = 1.33; 95% CI: 1.02–1.74; I^2^ = 0.0%; *p* = 0.882), right ventricle fractional area change (SMD = 0.18; 95% CI: 0.01–0.36; I^2^ = 0.0%; *p* = 0.440), systolic pulmonary arterial pressure (SMD = 0.97; 95% CI: 0.76–1.19; I^2^ = 41.5%; *p* = 0.181), and proper ventricular systolic pressure (SMD = 1.07; 95% CI: 0.54–1.59; I^2^ = 92.4%; *p* < 0.000) may negatively influence significant TR.

**Conclusions:**

This meta-analysis identified a potential negative influence of several clinical characteristics, echocardiography parameters, and previous comorbidities on significant TR. However, due to the low level of certainty of evidence, our analysis can only provide some guidance to practitioners and researchers. Caution is advised, and further validation is needed.

## Introduction

Atrial fibrillation (AF), an epidemic disease with an increasing global public health burden, is the most common in-hospital arrhythmia in clinical practice (1) and is increasingly found to be the primary reason for morbidity and mortality in cardiovascular diseases. Recently, the prevalence of AF has been increasing, and it is estimated that one in every four to six adults will suffer from AF in their lifetime (2, 3). Clinically, AF is usually treated and controlled by drugs or ablation (4). Despite the maintenance of normal ventricular systolic function and size, a prolonged duration of illness can still result in atrial and annular dilation due to AF. This dilation may subsequently lead to a series of secondary or functional heart changes, including tricuspid (TR) and mitral regurgitation (MR) (5–7). In recent years, a large number of studies (8–10) have focused on the impact of significant TR (moderate or severe TR) in patients with AF; that is, significant TR can lead to progressive right ventricular enlargement and increased morbidity and mortality in heart failure (HF) (8–10). Currently, epidemiological evidence shows that the prevalence of TR (including significant TR) in the elderly population has markedly increased, approximately 8%–9% (10, 11), and AF was considered one of the main causes of atrial functional TR (12). Therefore, finding potential predictors of significant TR in patients with AF as early and as accurately as possible is vital for patients (especially the elderly), healthcare providers, and policymakers.

The factors associated with significant TR in AF typically include clinical characteristics, echocardiographic parameters, and prior comorbidities (13–15). Although not all of these factors, such as sex, can be changed, unchangeable predictive factors can also play a preventive and indicative role (16–18). In recent years, many studies (16, 19, 20) have reported some potential predictive factors, but most were observational studies. So far, only four published meta-analyses have focused on TR patients. Of these, three (21–23) focused on the independent risk factors of cardiac implantation (permanent pacemaker or cardioverter defibrillator) for TR, and the other (24) discussed the risk factors for TR after valve surgery. Unfortunately, no meta-analysis has reviewed the comprehensive predictive value of clinical characteristics, echocardiographic parameters, and prior comorbidities in multicenter clinical trials of a large size. Thus, this study was intended to identify the potential predictors of significant TR in AF patients based on open databases.

## Methods

This systematic review and meta-analysis was guided by the Preferred Reporting Items for Systematic Reviews and Meta-Analyses (PRISMA 2020) statement (25).

### Search strategy

PubMed, EMBASE, the Cochrane Library, and the Web of Science database were comprehensively searched for relevant studies from their inception until September 2023. The study used “Atrial Fibrillations,” “Auricular Fibrillation”, “Tricuspid Valve Incompetence”, “Tricuspid Valve Regurgitation”, “Significant Tricuspid Incompetence”, “Significant Tricuspid Regurgitation”, and other relevant keywords in the medical subject heading (MeSH) terms to establish the search strategy (Supplementary Table S1).

### Inclusion and exclusion criteria

The inclusion criteria were as follows:

1. Population: AF patients.
2. Intervention: significant TR (patients with a clinical qualitative or quantitative diagnosis of moderate to severe TR).
3. Comparator: non-significant TR (no TR and mild TR).
4. Outcome: including at least one of the following factors: clinical characteristics (age, age ≥ 65 years, female), echocardiography parameters [left ventricular ejection fraction (LVEF), systolic pulmonary arterial pressure (SPAP), right ventricle fractional area change (RV FAC), and right ventricular systolic pressure (RVSP)], and previous comorbidities [persistent atrial fibrillation (PAF), coronary artery disease (CAD), chronic kidney disease (CKD), HF, diabetes mellitus (DM), hypertension, and chronic lung disease (CLD)].

The exclusion criteria were as follows:

1. Article type: conference abstracts, case reports, and reviews.
2. Duplicate reports.

### Data extraction

The included studies were selected by two independent reviewers, and disagreements were resolved in consultation with a third reviewer. We extracted the author's name, publication year, country, study design, mean age, sample size, female proportion, target population, and median follow-up time from the included studies.

### Quality assessment

Risk Of Bias In Non-Randomized Studies - of Interventions (ROBINS-I), a method to evaluate bias risk in non-randomized studies, was selected to assess the methodological quality of the included articles (http://www.riskofbias.info). Based on the assessment results, the bias risk of the studies was classified as “Low”, “Moderate”, “Serious”, or “Critical”.

Based on the outcome evaluation results and characteristics of the included studies, an evidence certainty assessment table was created using the GRADE system (26, 27).

### Statistical analysis

This study used STATA 14.0 software (StataCorp, College Station, TX, USA). Hazard ratio (HR), standard mean difference (SMD), and 95% confidence intervals (CIs) were used to assess the clinical characteristics, echocardiography parameters, and the results of prior comorbidities. The χ^2^ test and I-squared (I^2^) statistic were used to assess the heterogeneity. The random effects model was used if *p* ≤ 0.10 and I^2^ ≥ 50%, which meant existing heterogeneity among studies model. Otherwise, the fixed-effect model was applied. Begg’s rank correlation (28), funnel plots, and Egger’s weighted regression (29) were performed to test the publication bias. Trim-and-fill analysis was conducted to judge whether the publication bias impacted the outcome synthesis if significant bias was present. Subgroup analysis was used to explore possible causes of heterogeneity if necessary. The robustness of the results in this study was determined by leave-one-out analysis. *p* < 0.05 indicated statistical significance.

## Results

### Study screening

**Figure 1:**
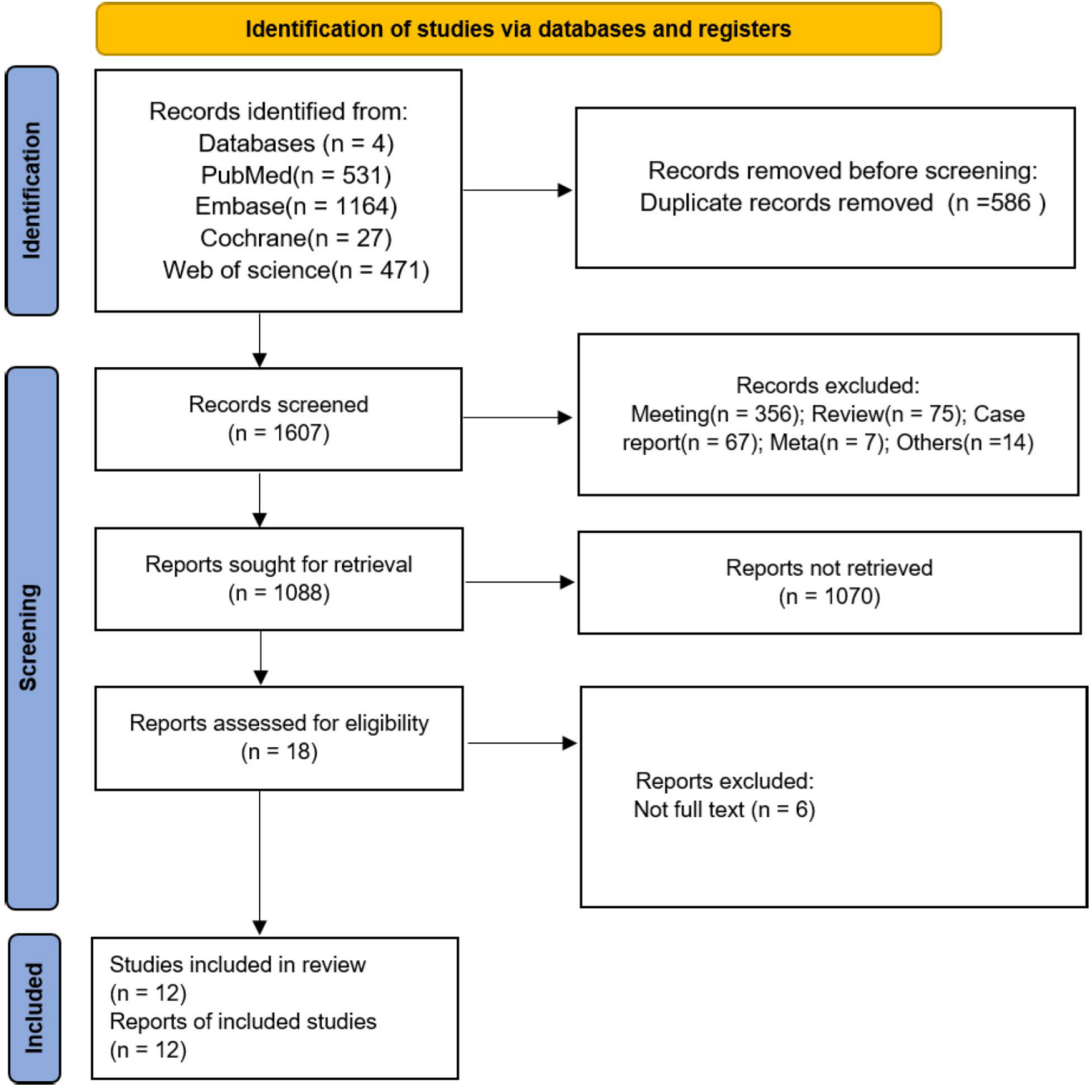
PRISMA flow chart for study screening and inclusion.

A total of 2,193 studies were retrieved as potential reports from four open databases. After the initial removal of 586 duplicate records, 1,607 articles were reserved for title and abstract review, and 519 articles with ineligible study designs were excluded. After excluding 1,070 articles, 18 articles remained for full-text review, and, ultimately, 12 studies (8, 13–20, 30–32) were included in the meta-analysis. The flow chart is shown in Figure 1.

### Study characteristics

The 12 retrospective studies that met the inclusion criteria were published between 2015 and 2023, and the sample sizes ranged from 71 to 4,613. Almost 16,000 patients were included in this review. The included studies were conducted in Korea, Japan, Romania, Thailand, and America. The mean age of the included study population ranged from 63 to 78, and the female proportion ranged from 27.6% to 64.7%. The basic characteristics are outlined in Table 1.

**Table 1:** Baseline characteristics of the included studies.

Study ID	Country	Study design	Simple size (I/C)	Target population	Age (years) (mean ± SD)	Sex, female (%)	Median follow-up time (months)	Outcomes
Min Soo Cho 2023	Korea	Retrospective	164/4,449	Non-valvular AF patients without significant structural abnormalities	63.0 ± 11.3	30.3	34.8 (IQR: 14.4–63.6)	Age ≥65 years, Female, LVEF, PAF, HF, CKD
Yuko Yamamoto 2022	Japan	Retrospective	80/264	Patients with persistent AF	73.0 ± 9.3	27.6	NA	Age, female, LVEF, CAD, HF, CKD, DM, hypertension, CLD, RV FAC
Ancuta Elena Vîjan 2022	Romania	Retrospective	20/226	Patients with AF	71.5 ± 9.4	63	34 (IQR: 28–39)	LV EF, PAF, HF, SPAP
Natthaporn Prapan 2020	Thailand	Retrospective	65/235	AF patients with LVEF	68.8 ± 10.8	50	33.84 (IQR: 23.52–49.68)	Age, female, LVEF, PAF, SPAP
Sri Harsha Patlolla 2022	USA	Retrospective	232/459	Adult patients with new-onset AF	68.0 (IQR: 58.0–76.0)	38.9	159.6 (IQR: 120–190.8)	Age ≥65 years, female, LVEF, PAF, CAD, HF, CKD, DM, Hypertension, CLD
Susan X. Zhao 2017	USA	Retrospective	81/89	Patients with chronic AF in the absence of structural or known coronary heart disease.	73 ± 11	56	39.6 ± 22.8	Age, female, LVEF, RV FAC, RVSP
Taishi Fujisawa 2022	Japan	Retrospective	1,107/1,104	Newly diagnosed or referred AF patients with no prior history of HF who were with known TR grade at baseline	66.9 ± 11.2	30.3	24.3 (IQR: 12.2–24.4)	Age, female, LVEF, PAF, CAD, DM, hypertension, CLD
Jiyeon Song 2023	Korea	Retrospective	68/219	Patients with persistent AF	66.9 ± 11.4	39	58.8	Age ≥65 years, LVEF
Jae Yeong Cho 2016	Korea	Retrospective	55/16	Patients with severe TR	Experimental group: 72.3 ± 11.8; control: 70.1 ± 15.5	64.7	NA	LVEF, RVSP
Yukio Abe 2018	Japan	Retrospective	33/241	Patients with AF and LVEF ≥50% but without other underlying heart diseases	Experimental group: 78 ± 9; control: 71 ± 10	39	24 ± 17	LVEF, RVSP
Yong Soo Kim 2023	Korea	Retrospective	877/6,009	Consecutive acute ischemic stroke patients	Experimental group: 75.8 ± 10.1; control: 66.7 ± 13.5	40	12	LVEF, RVSP
Jae-Hyung Park 2015	Korea	Retrospective	49/40	Patients with lone AF	075.2 ± 11.2	48.3	NA	LVEF, RV FAC, SPAP

IQR, interquartile range; NA, not available.

### Quality assessment

**Figure 2:**
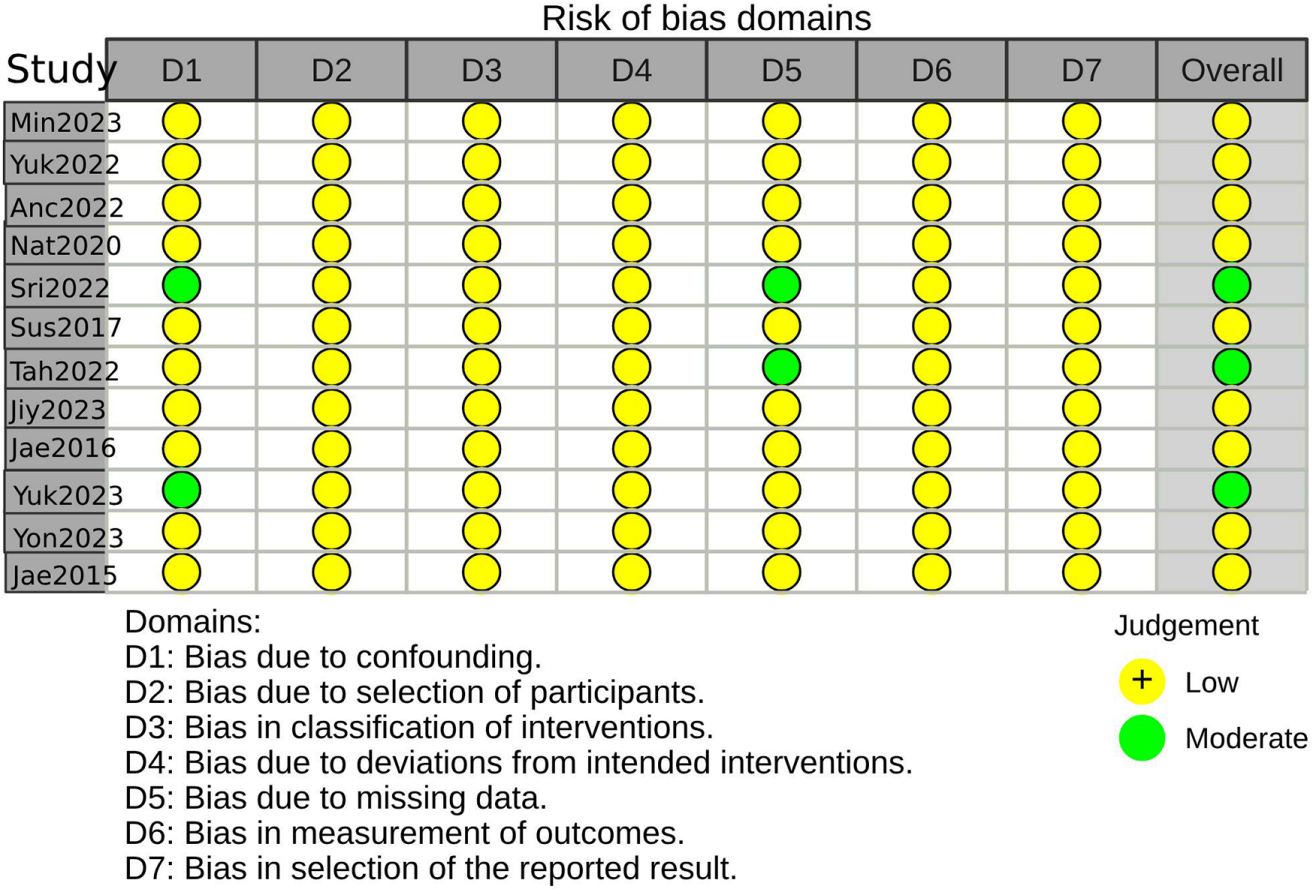
Risk-of-bias summary using the ROBINS-I(a).

**Figure 3:**
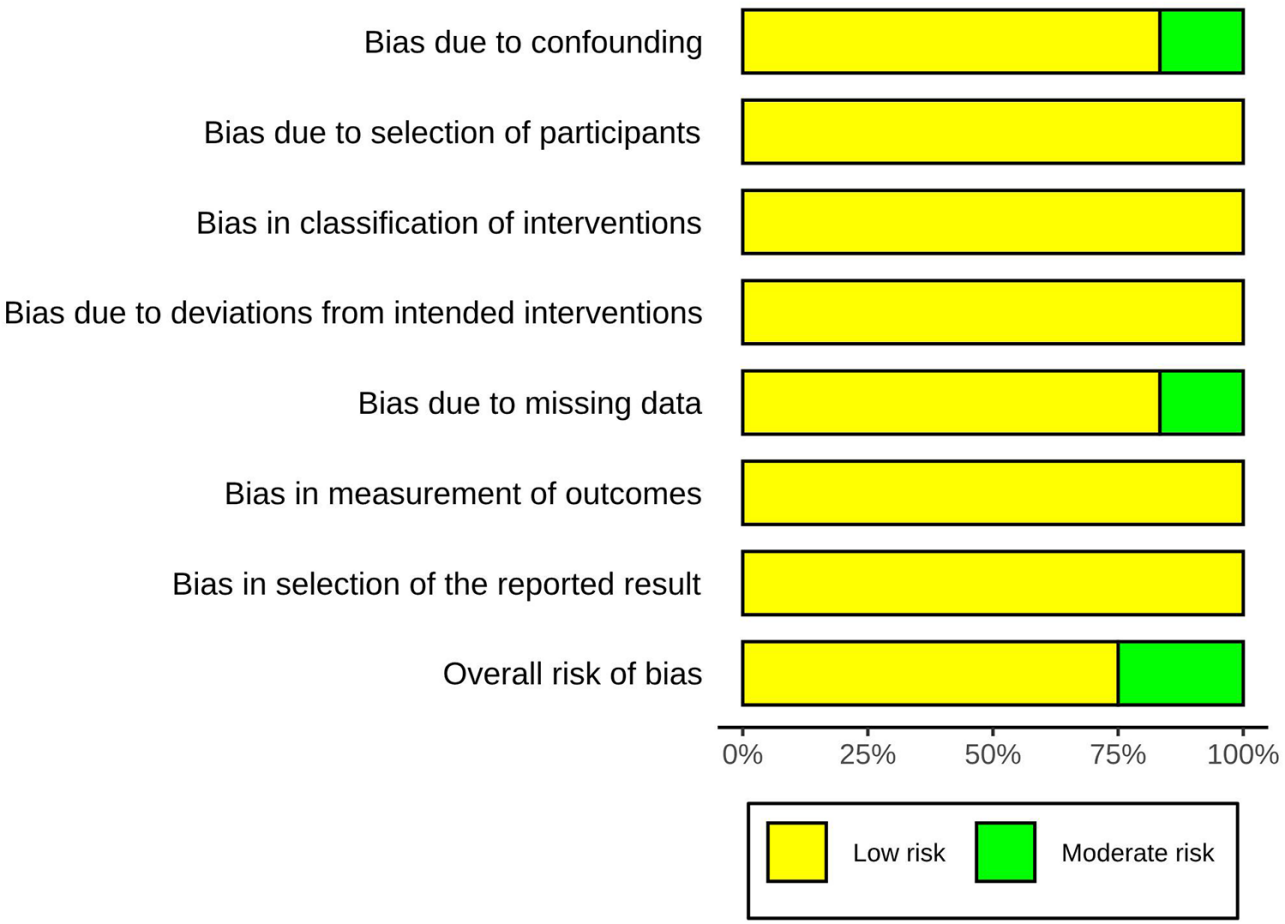
Risk-of-bias summary using the ROBINS-I(b).

Three studies were considered to be of moderate risk due to confounding and missing data. The other nine were evaluated as low risk (Figures 2, 3, Supplementary Table S2). The certainty of evidence is summarized in Table 2 using the GRADE system. Pooled results of HR or SMD for each predictor below are also presented in order from higher to lower GRADE certainty levels.

**Table 2:** Evidence certainty of predictors based on the GRADE Working Group grades of evidence.

Predictor	Study, *n*	Study design	Risk of bias	Inconsistency	Indirectness	Imprecision	Other considerations	Certainty	Rank
Female	6	Observational	Not Serious	Not Serious	Not Serious	Not Serious	None	Moderate	⊕⊕⊕〇
PAF	5	Observational	Not Serious	Not Serious	Not Serious	Not Serious	None	Moderate	⊕⊕⊕〇
LV ejection fraction	10	Observational	Not Serious	Not Serious	Not Serious	Not Serious	None	Moderate	⊕⊕⊕〇
Age	4	Observational	Not Serious	Not Serious	Not Serious	Not Serious	None	Low	⊕⊕〇〇
HF	4	Observational	Not Serious	Not Serious	Not Serious	Not Serious	None	Low	⊕⊕〇〇
Age ≥65 years	3	Observational	Not Serious	Not Serious	Not Serious	Not Serious	None	Low	⊕⊕〇〇
CLD	3	Observational	Not Serious	Not Serious	Not Serious	Not Serious	None	Low	⊕⊕〇〇
RV FAC	3	Observational	Not Serious	Not Serious	Not Serious	Not Serious	None	Low	⊕⊕〇〇
SPAP	3	Observational	Not Serious	Not Serious	Not Serious	Not Serious	None	Low	⊕⊕〇〇
RVSP	4	Observational	Not serious	Not serious	Not serious	Not serious	None	Low	⊕⊕〇〇
LVEF	4	Observational	Not serious	Serious	Not serious	Not serious	None	Very low	
CAD	3	Observational	Not serious	Serious	Not serious	Not serious	None	Very low	
CKD	3	Observational	Not serious	Serious	Not serious	Not serious	None	Very low	
DM	3	Observational	Not serious	Serious	Not serious	Not serious	None	Very low	
Hypertension	3	Observational	Not serious	Serious	Not serious	Not serious	None	Very low	

GRADE Working Group grades of evidence:

High quality: Further research is very unlikely to change our confidence in the estimate of effect. Moderate quality: Further research is likely to have an important impact on our confidence in the estimate of effect and may change the estimate. Low quality: Further research is very likely to have an important impact on our confidence in the estimate of effect and is likely to change the estimate. Very low quality: We are very uncertain about the estimate.

### Predictors of TR

**Figure 4:**
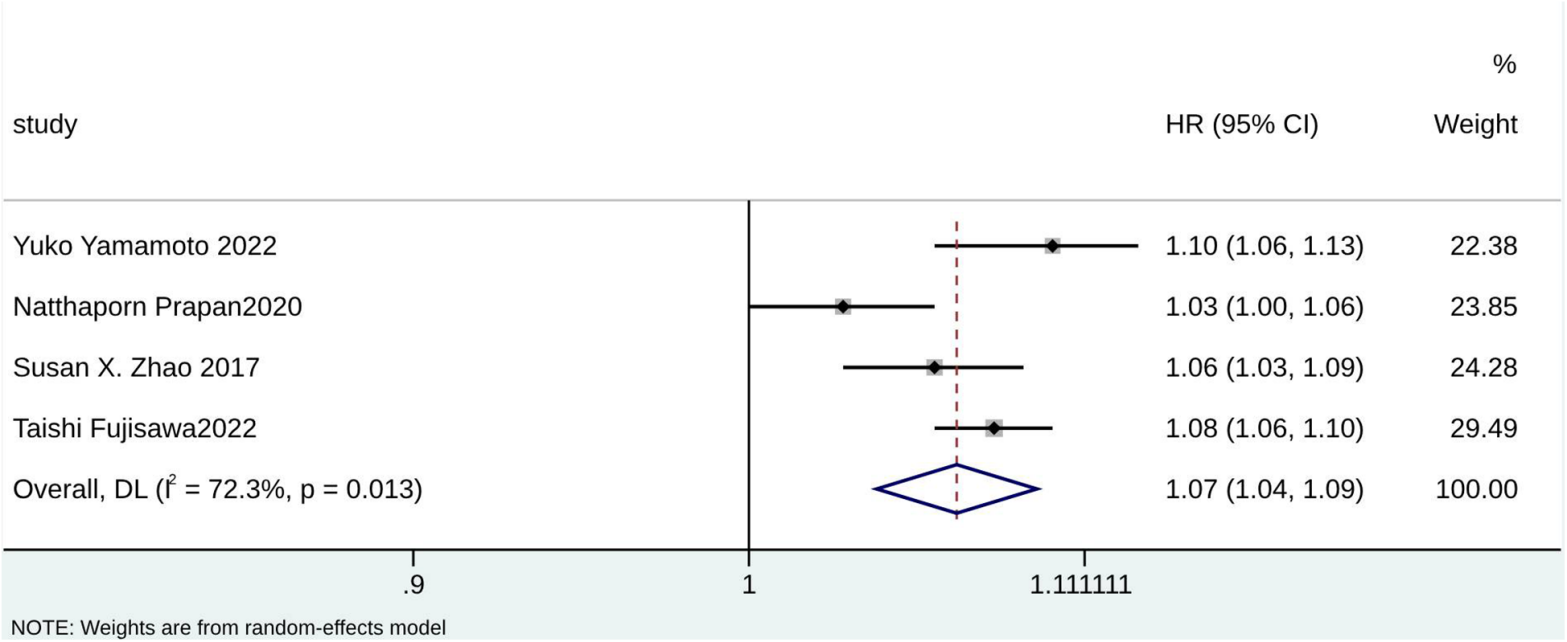
Forest plot for age.

**Figure 5:**
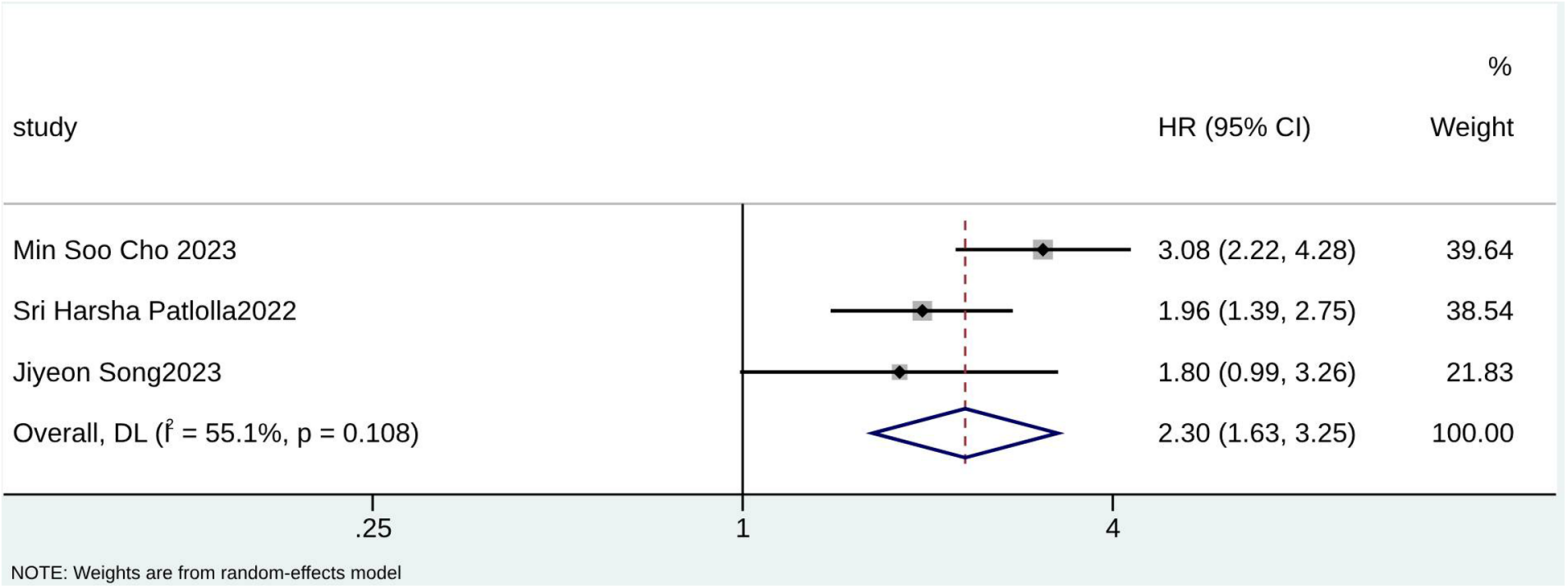
Forest plot for age ≥65 years.

**Figure 6:**
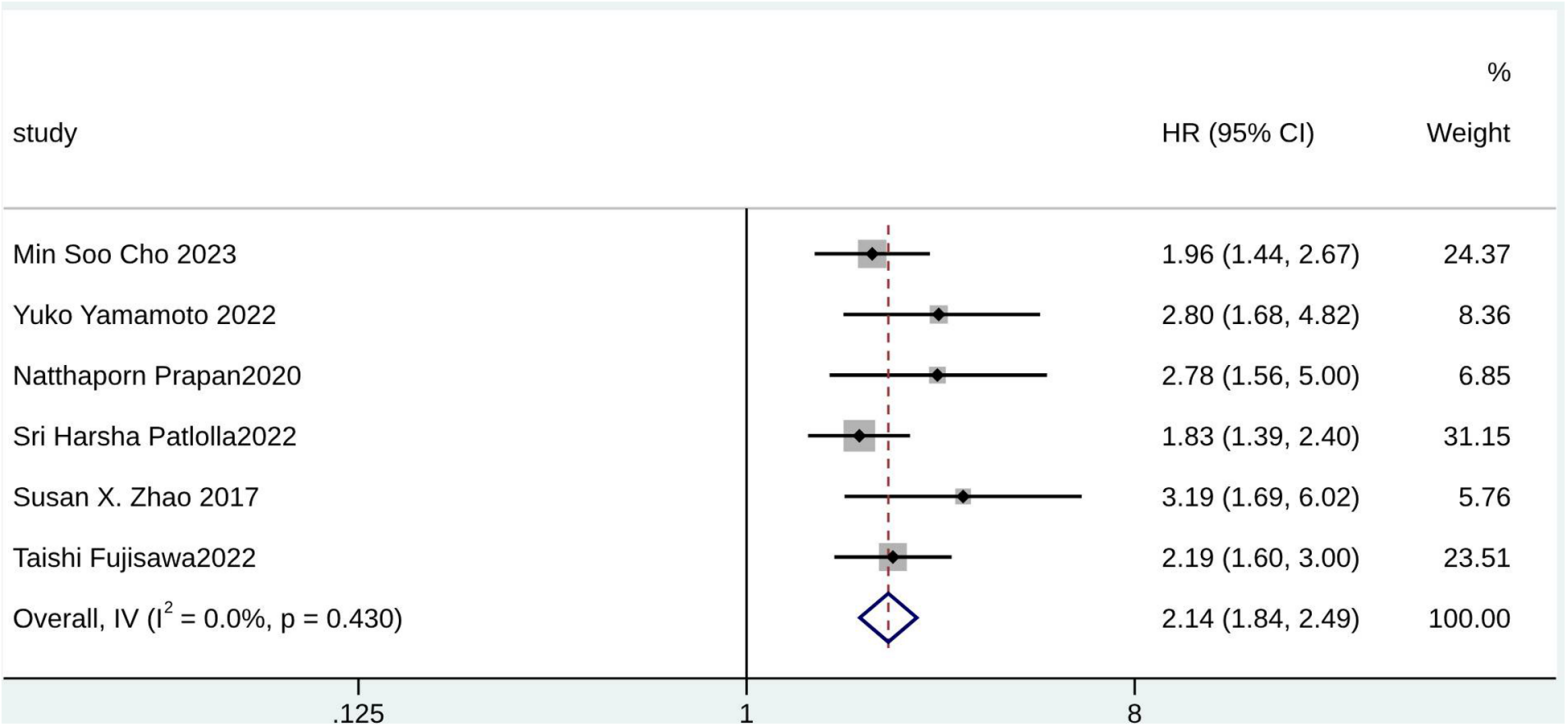
Forest plot for female sex.

According to the pooled analysis of clinical characteristics, female sex (HR = 2.14; 95% CI: 1.84–2.49; I^2^ = 0.0%; *p* = 0.430), age (HR = 1.07; 95% CI: 1.04–1.09; I^2^ = 72.3%; *p* = 0.013), and age ≥65 years (HR = 2.30; 95% CI: 1.63–3.25; I^2^ = 55.1%; *p* = 0.108) were potential predictors for TR in patients with AF (Figures 4–6). The GRADE evidence certainty level for female sex was the highest (moderate) of all the predictors, followed by age and age ≥65 years (low).

**Figure 7:**
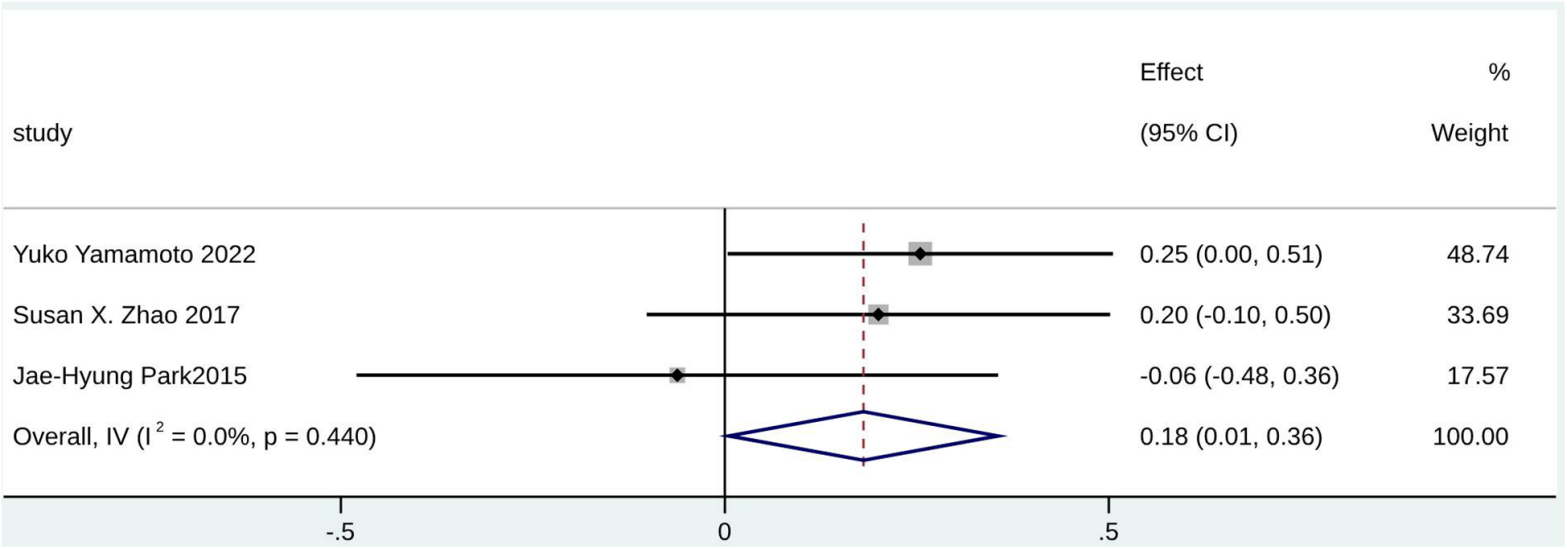
Forest plot for RV FAC.

Furthermore, echocardiography parameters including RV FAC (SMD = 0.18; 95% CI: 0.01–0.36; I^2^ = 0.0%; *p* = 0.440), SPAP (SMD = 0.97; 95% CI: 0.76–1.19; I^2^ = 41.5%; *p* = 0.181), and RVSP (SMD = 1.07; 95% CI: 0.54–1.59; I^2^ = 92.4%; *p* < 0.000) were potential predictors for significant TR in patients with AF (Figure 7, Supplementary Figures S32, S33). However, RVSP had high heterogeneity. A statistically significant difference was found for LVEF (SMD = −0.16; 95% CI: −0.30 to −0.03; I^2^ = 69.8%; *p* < 0.000) (Supplementary Figure S34), which may serve as a potential predictor for non-significant TR in patients with AF (HR = 1.00; 95% CI: 0.96–1.03; I^2^ = 71.7%; *p* = 0.014) (Supplementary Figure S35).

Regarding previous comorbidities, PAF (HR = 2.99; 95% CI: 2.47–3.61; I^2^ = 0.0%; *p* = 0.896), HF (HR = 1.86; 95% CI: 1.45–2.39; I^2^ = 9.0%; *p* = 0.348), and CLD (HR = 1.33; 95% CI: 1.02–1.74; I^2^ = 0.0%; *p* = 0.882) were identified as potential predictors for significant TR in patients with AF (Supplementary Figures S36–S38). However, statistically significant associations were not observed for CAD (HR = 1.10; 95% CI: 0.72–1.68; I^2^ = 57.7%; *p* = 0.094), CKD (HR = 1.11; 95% CI: 0.55–2.26; I^2^ = 90.7%; *p* < 0.000), DM (HR = 0.76; 95% CI: 0.42–1.36; I^2^ = 78.7%; *p* = 0.009), and hypertension (HR = 1.07; 95% CI: 0.87–1.32; I^2^ = 0.0%; *p* = 0.688) (Supplementary Figures S39–S42).

### Publication bias and sensitivity analysis

The funnel plots showed that there may be a publication bias in some results, such as LVEF (HR) and RV FAC (Supplementary Figures S1–S15), but this possibility was negated by Begg’s and Egger's tests (Supplementary Table S3). The trim-and-fill results showed that bias may have impacted the pooled result for the female sex (Supplementary Figure S3^1^). The sensitivity analysis indicated robust results (Supplementary Figures S16–S30).

## Discussion

This study systematically reviewed predictors of significant TR in AF patients. We identified 10 potential predictors for significant TR in AF patients, including age, age ≥65 years, female, LVEF (SMD), RV FAC, SPAP, RVSP, PAF, HF, and CLD.

Demographic characteristics, such as age, sex, and BMI, are well-known factors associated with significant TR occurrence (33, 34). The type and number of collagen fibers in the tricuspid annulus differ with age, which may be the main reason why the gradual expansion of the tricuspid orifice and the more elliptical evolution of the triangle geometry play a role in the progression of age-affected TR (35, 36). In aging populations, the number of elderly patients with long-term AF is increasing (37–39). Therefore, it is necessary to find predictors of significant TR in AF patients over 65 years of age. A study found sex differences among clinical characteristics, valve anatomy, and morphological characteristics in AF patients with TR (40). Compared to male patients, female patients exhibited a higher circumferential index but fewer cells and less elasticity in the valve ring (41). This may indicate that sex has a predictive value for significant TR in AF patients. Consistent with existing literature, this study indicated that age, age ≥65 years, and sex were potential predictors for significant TR in AF and proved the predictive advantages of these clinical factors statistically. Moreover, significant TR in female AF patients aged ≥65 should be investigated in future clinical practice.

In addition, some echocardiographic parameters related to cardiac remodeling, such as LVEF, tricuspid annulus diameter (TAD), RV FAC, stent height, and some hemodynamic parameters, such as SPAP and RVSP, have also been confirmed to be associated with significant TR in AF in previous studies (10, 13). Several cohort studies have observed that moderate pulmonary hypertension in AF patients may be accompanied by left ventricular diastolic dysfunction or organic changes (such as tricuspid annulus changes), suggesting that SPAP may be related to the occurrence or progression of TR (42, 43). In addition, a study (31) showed that RVSP increased during the follow-up of patients with reversible TR, which may be related to respiratory variability and the inferior vena cava (IVC) size. As for parameters such as LVEF, a retrospective study (31) showed that LVEF in AF patients increasing by more than 10% may promote an improvement in reversible TR. This study showed that LVEF, RV FAC, SPAP, and RVSP were all predictive factors of significant TR in AF, proving these factors’ predictive advantages statistically. Other parameters, such as left atrial diameter (LAD), TAD, and right atrial diameter (RAD), have not been discussed in this study because it was difficult to obtain enough data. The correlation between these parameters and significant TR in patients with AF should be analyzed in subsequent research.

It is believed that the progression of AF patients with or without significant TR may be regulated by previous comorbidities (44), and the mortality of patients with simple AF was lower than that of patients with prior comorbidities (45). For AF patients with significant TR, the common comorbidities include PAF, HF, CLD, CAD, CKD, DM, and hypertension (16, 19, 20). Several studies have shown that long-term AF, a type of arrhythmia, can lead to right atrial dilatation. Maintaining sinus rhythm (rhythm control) may help prevent significant TR. This suggests a potential correlation between non-paroxysmal AF (or persistent atrial fibrillation) and significant TR in patients with AF (46, 47). In addition, 2-year follow-up data from previous studies showed that 36.9% of AF patients with significant TR developed heart failure even with preserved LVEF (9, 15), and nearly two-thirds developed new-onset lung disease (14). All the studies mentioned above demonstrate a meaningful correlation between prior comorbidities and TR in AF. This study specifically identified PAF, HF, and CLD as predictors for significant TR in AF, providing statistical evidence for the predictive value of these factors. However, our results did not support a correlation between significant TR and CAD, CKD, DM, and hypertension in patients with AF. In addition to prior comorbidities mentioned above, dyslipidemia, smoking, and alcohol abuse had not been discussed because it was difficult to obtain sufficient data. The correlation between these factors and significant TR in patients with AF should be analyzed in subsequent research.

Given the limitations of the current stage of the study, the original data only support a broad analysis of the relationship between various influencing factors and the occurrence of significant TR in patients with AF. Several limitations were identified during the study. First, potential language bias existed because only studies published in English were included. Second, due to the rarity of isolated TR and the limited number of original studies, subgroup analysis according to different AF characteristics was not performed. In subsequent research, it is necessary to discuss the predicted value of predictors of TR in AF patients according to the following subgroups: primary, secondary (caused by left valvular diseases, left ventricular systolic dysfunction, or pulmonary hypertension unrelated to any heart disease), isolated TR (accounts for 8%–10% of all TR patients approximately and there seem to be no apparent causes), persistent TR, and new-onset TR. Third, the enrolled sample size was limited. The discussion on the correlation between significant TR and more clinical characteristics, echocardiography parameters, follow-up durations, and prior comorbidities is of great clinical significance. However, the number of studies that can be included is minimal, which may make it difficult to obtain convincing results. Fortunately, no publication bias existed in most of the results, and the sensitivity analysis indicated that the results were robust.

Clinical characteristics (including age, age ≥65 years, and female sex), echocardiographic parameters (including LVEF, RV FAC, RVSP, and SPAP), and prior comorbidities (including PAF, HF, and CLD) may serve as potential predictors for significant TR. Considering the limited cumulative amount of relevant evidence, further studies are needed to determine the long-term prognostic values of the above factors in significant TR, and more attention should be paid to looking for superior predictors of this aspect in the future.

## Data Availability

The original contributions presented in the study are included in the article/Supplementary Material, and further inquiries can be directed to the corresponding author.
