# Supplementary material for: Predictors of significant tricuspid regurgitation in atrial fibrillation: a meta-analysis
Source: Front Cardiovasc Med. 2025 Mar 6;12:1428964. doi: 10.3389/fcvm.2025.1428964 (PMC11922934; doi:10.3389/fcvm.2025.1428964)
Supplement: Supplementary file 2 [file Table2.docx]

**Search strategy of PubMed**

| NO. | Search Details | Results |
| --- | --- | --- |
| #7 | (#1 OR #3) AND (#2 OR #4) AND #5 | 531 |
| #6 | (#1 OR #3) AND (#2 OR #4) AND #5 | 612 |
| #5 | (((((predictor) OR (risk factor)) OR (risk)) OR (predictive factor)) OR (predict)) OR (determinant) | 14,556,576 |
| #4 | ((((((((((((((((((((((((((((Tricuspid Valve Regurgitation)) OR (Tricuspid Valve Incompetence)) OR (Tricuspid Incompetence)) OR (Tricuspid Regurgitation)) OR (right atrioventricular cardiac regurgitation)) OR (right atrioventricular cardiac valve insufficiency)) OR (right atrioventricular cardiac valvular insuffiency)) OR (right atrioventricular cardiac valvular regurgitation)) OR (right atrioventricular heart valve insufficiency)) OR (right atrioventricular heart valve regurgitation)) OR (right atrioventricular heart valvular regurgitation)) OR (right atrioventricular valve insufficiency)) OR (right atrioventricular valve regurgitation)) OR (right atrioventricular valvular insufficiency)) OR (right atrioventricular valvular regurgitation)) OR (tricuspid cardiac valve insufficiency)) OR (tricuspid cardiac valve regurgitation)) OR (tricuspid cardiac valvular insufficiency)) OR (tricuspid cardiac valvular regurgitation)) OR (tricuspid heart valve insufficiency)) OR (tricuspid heart valve regurgitation)) OR (tricuspid heart valvular insufficiency)) OR (tricuspid heart valvular regurgitation)) OR (tricuspid insufficiency)) OR (tricuspid valve insufficiency)) OR (tricuspid valvular insufficiency)) OR (tricuspid valvular regurgitation)) OR (tricuspidal insufficiency) | 15,171 |
| #3 | ((((((((((((((((((Atrial Fibrillations) OR (Auricular Fibrillation)) OR (Auricular Fibrillations)) OR (Persistent Atrial Fibrillation)) OR (Persistent Atrial Fibrillations)) OR (Familial Atrial Fibrillation)) OR (Familial Atrial Fibrillations)) OR (Paroxysmal Atrial Fibrillation)) OR (Paroxysmal Atrial Fibrillations)) OR (atrium fibrillation)) OR (auricular fibrilation)) OR (cardiac atrial fibrillation)) OR (cardiac atrium fibrillation)) OR (heart atrial fibrillation)) OR (heart atrium fibrillation)) OR (heart fibrillation atrium)) OR (non-valvular atrial fibrillation)) OR (nonvalvular atrial fibrillation)) OR (atrial fibrillation) | 109,068 |
| #2 | "Tricuspid Valve Insufficiency"[Mesh] | 7,314 |
| #1 | "Atrial Fibrillation"[Mesh] | 71,847 |

**Search strategy of EMBASE**

| No. | Query | Results |
| --- | --- | --- |
| #7 | #6 AND 'human'/de | 1164 |
| #6 | (#1 OR #2) AND (#3 OR #4) AND #5 | 1206 |
| #5 | 'predictor':ti,ab,kw OR 'risk factor':ti,ab,kw OR 'risk':ti,ab,kw OR 'predictive factor':ti,ab,kw OR 'predict':ti,ab,kw OR 'determinant':ti,ab,kw | 4864458 |
| #4 | 'tricuspid valve regurgitation':ti,ab,kw OR 'tricuspid valve incompetence':ti,ab,kw OR 'tricuspid incompetence':ti,ab,kw OR 'tricuspid regurgitation':ti,ab,kw OR 'right atrioventricular cardiac regurgitation':ti,ab,kw OR 'right atrioventricular cardiac valve insufficiency':ti,ab,kw OR 'right atrioventricular cardiac valvular insuffiency':ti,ab,kw OR 'right atrioventricular cardiac valvular regurgitation':ti,ab,kw OR 'right atrioventricular heart valve insufficiency':ti,ab,kw OR 'right atrioventricular heart valve regurgitation':ti,ab,kw OR 'right atrioventricular heart valvular regurgitation':ti,ab,kw OR 'right atrioventricular valve insufficiency':ti,ab,kw OR 'right atrioventricular valve regurgitation':ti,ab,kw OR 'right atrioventricular valvular insufficiency':ti,ab,kw OR 'right atrioventricular valvular regurgitation':ti,ab,kw OR 'tricuspid cardiac valve insufficiency':ti,ab,kw OR 'tricuspid cardiac valve regurgitation':ti,ab,kw OR 'tricuspid cardiac valvular insufficiency':ti,ab,kw OR 'tricuspid cardiac valvular regurgitation':ti,ab,kw OR 'tricuspid heart valve insufficiency':ti,ab,kw OR 'tricuspid heart valve regurgitation':ti,ab,kw OR 'tricuspid heart valvular insufficiency':ti,ab,kw OR 'tricuspid heart valvular regurgitation':ti,ab,kw OR 'tricuspid insufficiency':ti,ab,kw OR 'tricuspid valve insufficiency':ti,ab,kw OR 'tricuspid valvular insufficiency':ti,ab,kw OR 'tricuspid valvular regurgitation':ti,ab,kw OR 'tricuspidal insufficiency':ti,ab,kw | 16573 |
| #3 | 'tricuspid valve regurgitation'/exp | 28365 |
| #2 | 'atrial fibrillations':ti,ab,kw OR 'auricular fibrillation':ti,ab,kw OR 'auricular fibrillations':ti,ab,kw OR 'persistent atrial fibrillation':ti,ab,kw OR 'persistent atrial fibrillations':ti,ab,kw OR 'familial atrial fibrillation':ti,ab,kw OR 'familial atrial fibrillations':ti,ab,kw OR 'paroxysmal atrial fibrillation':ti,ab,kw OR 'paroxysmal atrial fibrillations':ti,ab,kw OR 'atrium fibrillation':ti,ab,kw OR 'auricular fibrilation':ti,ab,kw OR 'cardiac atrial fibrillation':ti,ab,kw OR 'cardiac atrium fibrillation':ti,ab,kw OR 'heart atrial fibrillation':ti,ab,kw OR 'heart atrium fibrillation':ti,ab,kw OR 'heart fibrillation atrium':ti,ab,kw OR 'non-valvular atrial fibrillation':ti,ab,kw OR 'nonvalvular atrial fibrillation':ti,ab,kw OR 'atrial fibrillation':ti,ab,kw | 170813 |
| #1 | 'atrial fibrillation'/exp | 217347 |

**Search strategy of Cochrane Library**

| NO. | Search deatiles | Hits |
| --- | --- | --- |
| #1 | MeSH descriptor: [Atrial Fibrillation] explode all trees | 7431 |
| #2 | MeSH descriptor: [Tricuspid Valve Insufficiency] explode all trees | 107 |
| #3 | (Atrial Fibrillations):ti,ab,kw OR (Auricular Fibrillation):ti,ab,kw OR (Auricular Fibrillations):ti,ab,kw OR (Persistent Atrial Fibrillation):ti,ab,kw OR (Persistent Atrial Fibrillations):ti,ab,kw OR (Familial Atrial Fibrillation):ti,ab,kw OR (Familial Atrial Fibrillations):ti,ab,kw OR (Paroxysmal Atrial Fibrillation):ti,ab,kw OR (Paroxysmal Atrial Fibrillations):ti,ab,kw OR (atrium fibrillation):ti,ab,kw OR (auricular fibrilation):ti,ab,kw OR (cardiac atrial fibrillation):ti,ab,kw OR (cardiac atrium fibrillation):ti,ab,kw OR (heart atrial fibrillation):ti,ab,kw OR (heart atrium fibrillation):ti,ab,kw OR (heart fibrillation atrium):ti,ab,kw OR (non-valvular atrial fibrillation):ti,ab,kw OR (nonvalvular atrial fibrillation):ti,ab,kw OR (atrial fibrillation):ti,ab,kw | 16003 |
| #4 | (Tricuspid Valve Regurgitation):ti,ab,kw OR (Tricuspid Valve Incompetence):ti,ab,kw OR (Tricuspid Incompetence):ti,ab,kw OR (Tricuspid Regurgitation):ti,ab,kw OR (right atrioventricular cardiac regurgitation):ti,ab,kw OR (right atrioventricular cardiac valve insufficiency):ti,ab,kw OR (right atrioventricular cardiac valvular insuffiency):ti,ab,kw OR (right atrioventricular cardiac valvular regurgitation):ti,ab,kw OR (right atrioventricular heart valve insufficiency):ti,ab,kw OR (right atrioventricular heart valve regurgitation):ti,ab,kw OR (right atrioventricular heart valvular regurgitation):ti,ab,kw OR (right atrioventricular valve insufficiency):ti,ab,kw OR (right atrioventricular valve regurgitation):ti,ab,kw OR (right atrioventricular valvular insufficiency):ti,ab,kw OR (right atrioventricular valvular regurgitation):ti,ab,kw OR (tricuspid cardiac valve insufficiency):ti,ab,kw OR (tricuspid cardiac valve regurgitation):ti,ab,kw OR (tricuspid cardiac valvular insufficiency):ti,ab,kw OR (tricuspid cardiac valvular regurgitation):ti,ab,kw OR (tricuspid heart valve insufficiency):ti,ab,kw OR (tricuspid heart valve regurgitation):ti,ab,kw OR (tricuspid heart valvular insufficiency):ti,ab,kw OR (tricuspid heart valvular regurgitation):ti,ab,kw OR (tricuspid insufficiency):ti,ab,kw OR (tricuspid valve insufficiency):ti,ab,kw OR (tricuspid valvular insufficiency):ti,ab,kw OR (tricuspid valvular regurgitation):ti,ab,kw OR (tricuspidal insufficiency):ti,ab,kw | 516 |
| #5 | (predictor):ti,ab,kw OR (risk factor):ti,ab,kw OR (risk):ti,ab,kw OR (predictive factor):ti,ab,kw OR (predict):ti,ab,kw OR (determinant):ti,ab,kw | 318137 |
| #6 | (#1 or #3) and (#2 or #4) and #5 | 27 |

**Search strategy of Web of Science**

| NO. | Search deatiles | Hits |
| --- | --- | --- |
| #1 | (((((((((((((((((TS=(Atrial Fibrillations) OR TS=(Auricular Fibrillation)) OR TS=(Auricular Fibrillations)) OR TS=(Persistent Atrial Fibrillation)) OR TS=(Persistent Atrial Fibrillations)) OR TS=(Familial Atrial Fibrillation)) OR TS=(Familial Atrial Fibrillations)) OR TS=(Paroxysmal Atrial Fibrillation)) OR TS=(Paroxysmal Atrial Fibrillations)) OR TS=(atrium fibrillation)) OR TS=(auricular fibrilation)) OR TS=(cardiac atrial fibrillation)) OR TS=(cardiac atrium fibrillation)) OR TS=(heart atrial fibrillation)) OR TS=(heart atrium fibrillation)) OR TS=(heart fibrillation atrium)) OR TS=(non-valvular atrial fibrillation)) OR TS=(nonvalvular atrial fibrillation)) OR TS=(atrial fibrillation) | 128036 |
| #2 | (((((((((((((((((((((((((((TS=(Tricuspid Valve Regurgitation)) OR TS=(Tricuspid Valve Incompetence)) OR TS=(Tricuspid Incompetence)) OR TS=(Tricuspid Regurgitation)) OR TS=(right atrioventricular cardiac regurgitation)) OR TS=(right atrioventricular cardiac valve insufficiency)) OR TS=(right atrioventricular cardiac valvular insuffiency)) OR TS=(right atrioventricular cardiac valvular regurgitation)) OR TS=(right atrioventricular heart valve insufficiency)) OR TS=(right atrioventricular heart valve regurgitation)) OR TS=(right atrioventricular heart valvular regurgitation)) OR TS=(right atrioventricular valve insufficiency)) OR TS=(right atrioventricular valve regurgitation)) OR TS=(right atrioventricular valvular insufficiency)) OR TS=(right atrioventricular valvular regurgitation)) OR TS=(tricuspid cardiac valve insufficiency)) OR TS=(tricuspid cardiac valve regurgitation)) OR TS=(tricuspid cardiac valvular insufficiency)) OR TS=(tricuspid cardiac valvular regurgitation)) OR TS=(tricuspid heart valve insufficiency)) OR TS=(tricuspid heart valve regurgitation)) OR TS=(tricuspid heart valvular insufficiency)) OR TS=(tricuspid heart valvular regurgitation)) OR TS=(tricuspid insufficiency)) OR TS=(tricuspid valve insufficiency)) OR TS=(tricuspid valvular insufficiency)) OR TS=(tricuspid valvular regurgitation)) OR TS=(tricuspidal insufficiency) | 11690 |
| #3 | ((((TS=(predictor) OR TS=(risk factor)) OR TS=(risk)) OR TS=(predictive factor)) OR TS=(predict)) OR TS=(determinant) | 7070391 |
| #4 | #3 AND #2 AND #1 | 471 |
