# Supplementary material for: Predictors of significant tricuspid regurgitation in atrial fibrillation: a meta-analysis
Source: Front Cardiovasc Med. 2025 Mar 6;12:1428964. doi: 10.3389/fcvm.2025.1428964 (PMC11922934; doi:10.3389/fcvm.2025.1428964)
Supplement: Supplementary file 3 [file Table3.docx]

**Supplementary Table 2. Risk of bias assessment results of included studes by ROBINS-I**

| **Study** | **D1** | **D2** | **D3** | **D4** | **D5** | **D6** | **D7** | **Overall** |
| --- | --- | --- | --- | --- | --- | --- | --- | --- |
| Min Soo Cho 2023 | Low | Low | Low | Low | Low | Low | Low | Low |
| Yuko Yamamoto 2022 | Low | Low | Low | Low | Low | Low | Low | Low |
| Ancut,a Elena Vîjan2022 | Low | Low | Low | Low | Low | Low | Low | Low |
| Natthaporn Prapan2020 | Low | Low | Low | Low | Low | Low | Low | Low |
| Sri Harsha Patlolla2022 | Moderate | Low | Low | Low | Moderate | Low | Low | Moderate |
| Susan X. Zhao 2017 | Low | Low | Low | Low | Low | Low | Low | Low |
| Taishi Fujisawa2022 | Low | Low | Low | Low | Moderate | Low | Low | Moderate |
| Jiyeon Song2023 | Low | Low | Low | Low | Low | Low | Low | Low |
| Jae Yeong Cho 2016 | Low | Low | Low | Low | Low | Low | Low | Low |
| Yukio Abe 2018 | Moderate | Low | Low | Low | Low | Low | Low | Moderate |
| Yong Soo Kim 2023 | Low | Low | Low | Low | Low | Low | Low | Low |
| Jae-Hyung Park2015 | Low | Low | Low | Low | Low | Low | Low | Low |
| Domains:  D1: Bias due to confounding.  D2: Bias due to selection of participants.  D3: Bias in classification of interventions.  D4: Bias due to deviations from intended interventions.  D5: Bias due to missing data.  D6: Bias in measurement of outcomes.  D7: Bias in selection of the reported result. | | | | | | | | |
