# Supplementary material for: Predictors of significant tricuspid regurgitation in atrial fibrillation: a meta-analysis
Source: Front Cardiovasc Med. 2025 Mar 6;12:1428964. doi: 10.3389/fcvm.2025.1428964 (PMC11922934; doi:10.3389/fcvm.2025.1428964)
Supplement: Supplementary file 4 [file Datasheet1.docx]

**
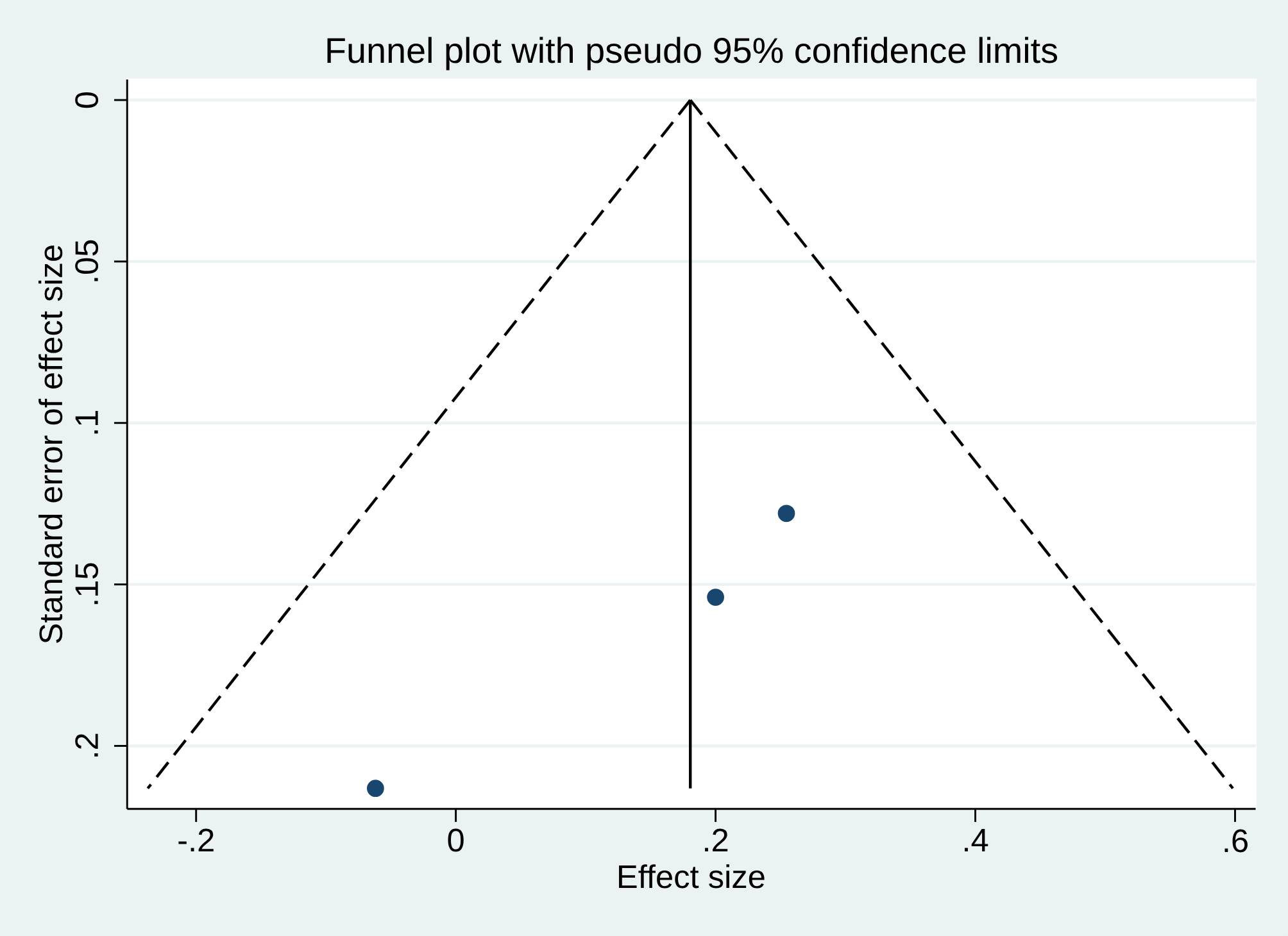
Supplementary Figure 1.**The funnel plot for Age

**
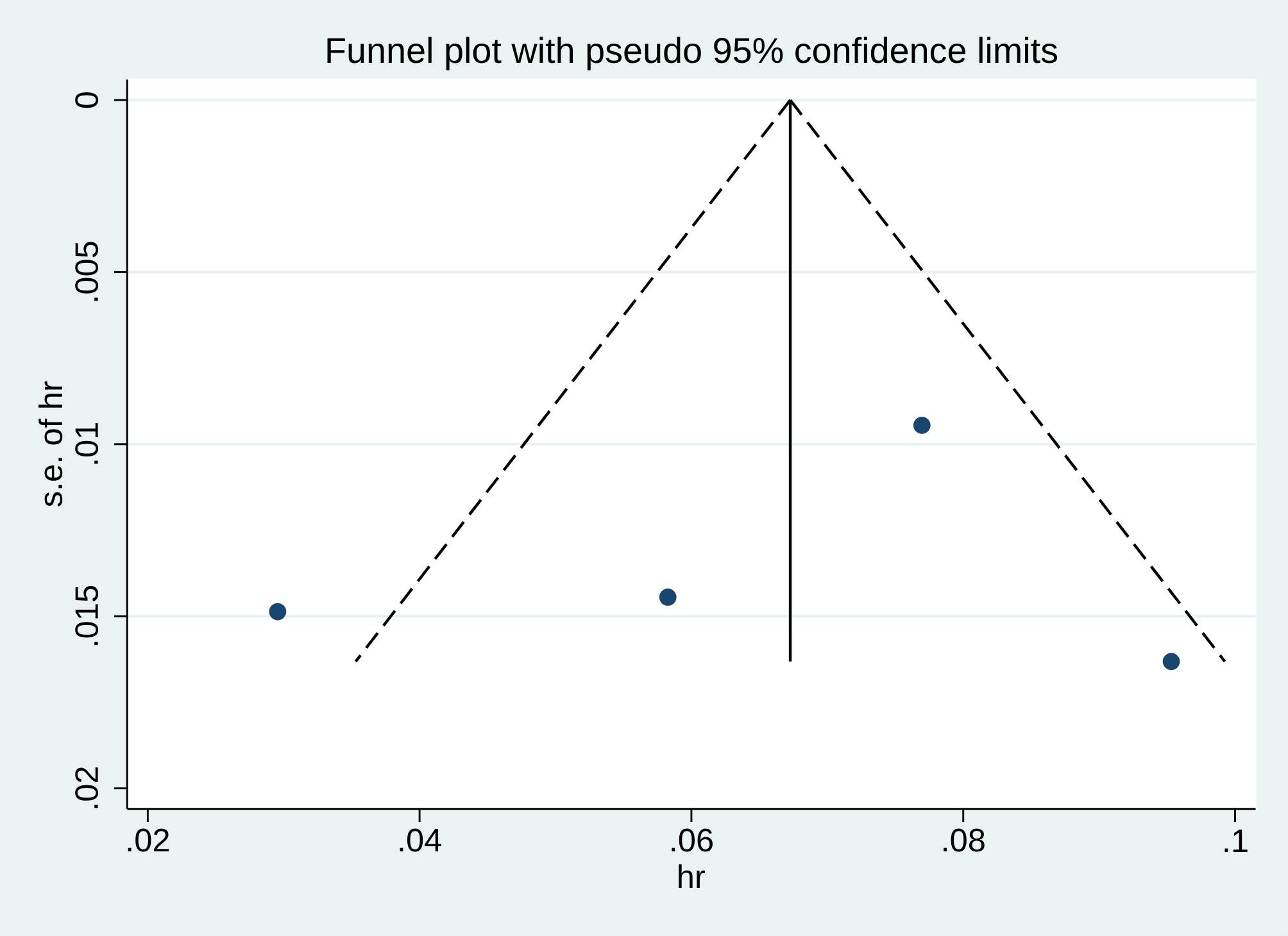
Supplementary Figure 2.**The funnel plot for Age ≥ 65 years.

**
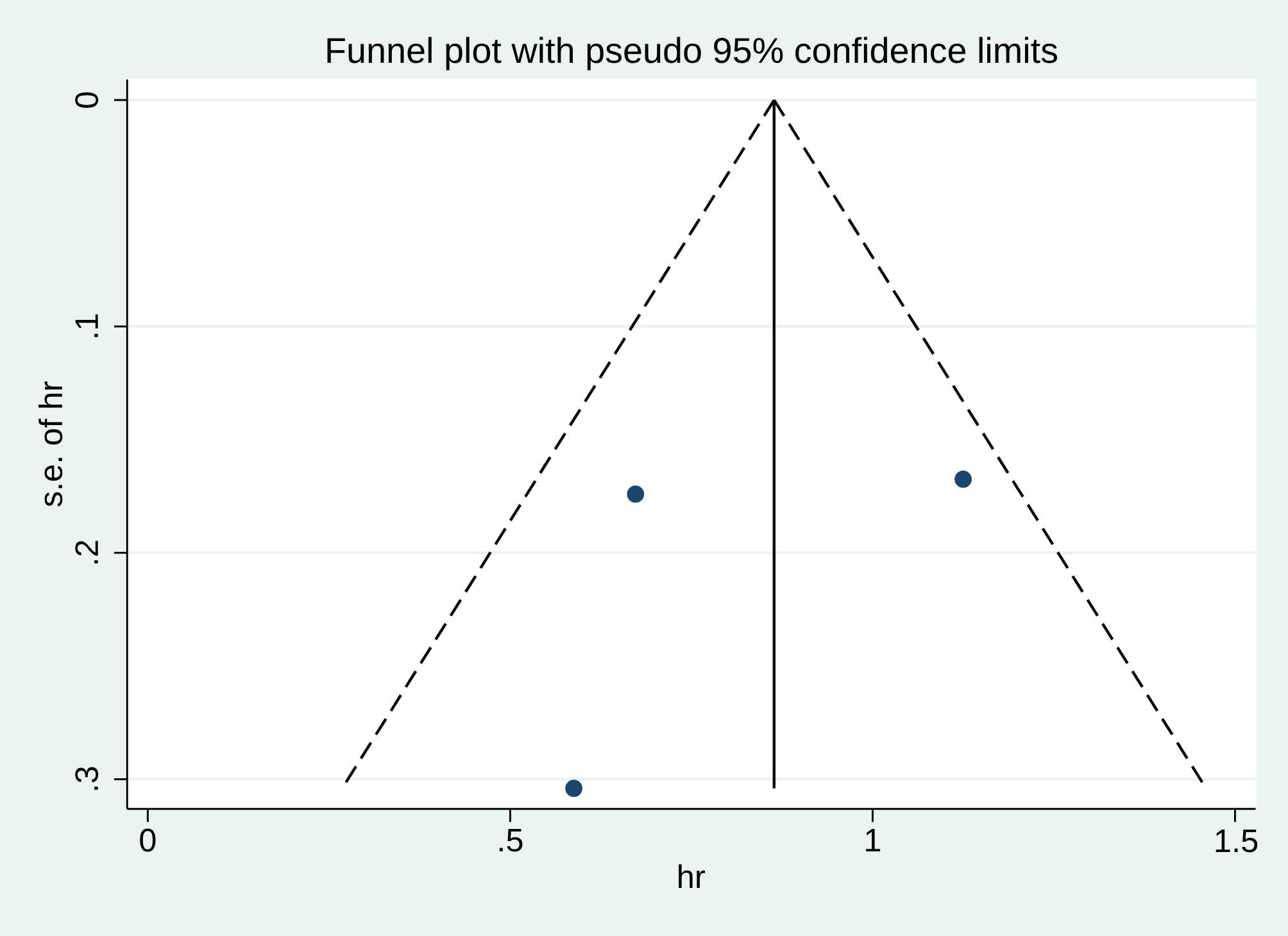
Supplementary Figure 3.**The funnel plot for Female

**
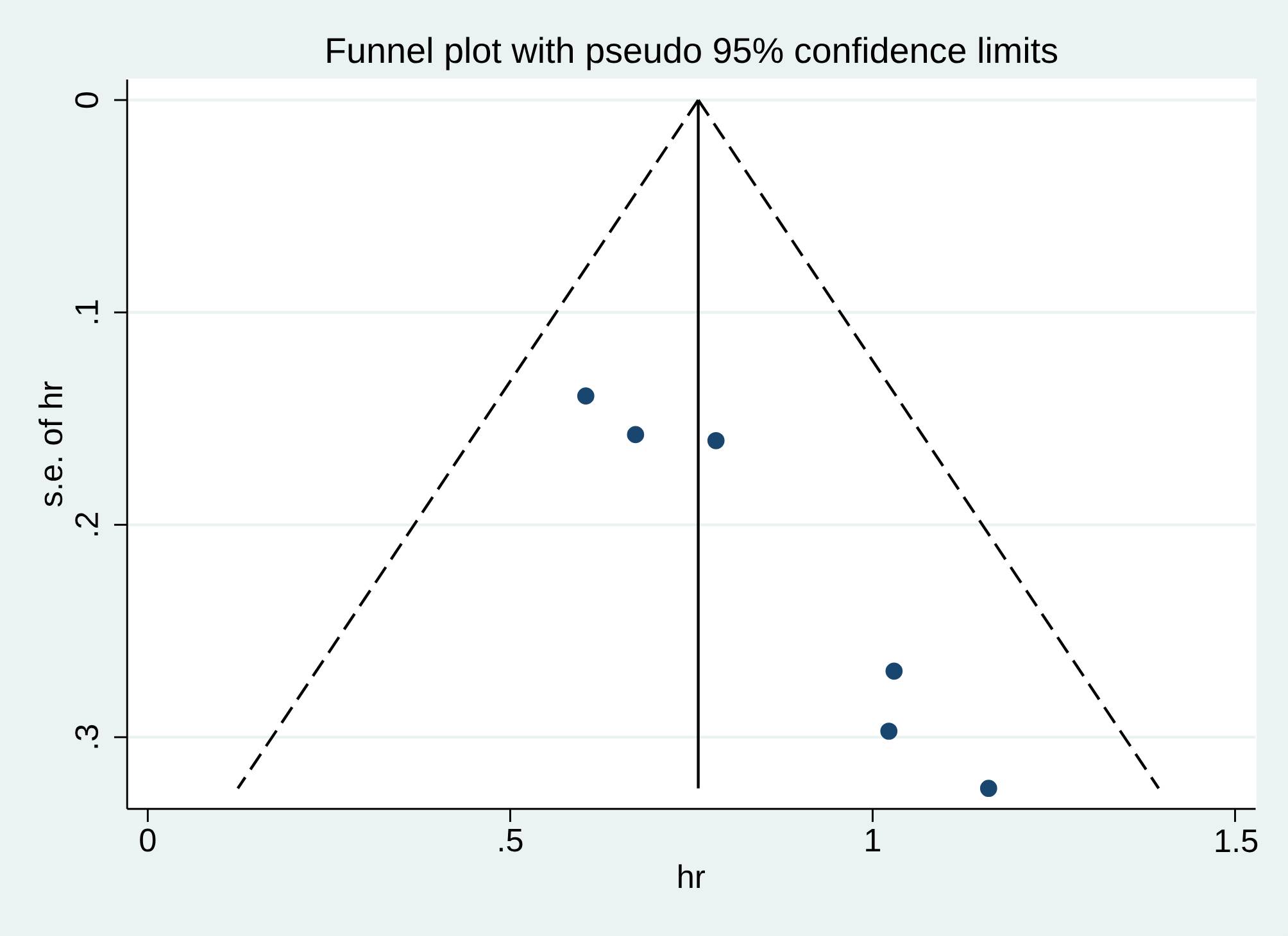
Supplementary Figure 4.**The funnel plot for LVEF(HR)

**
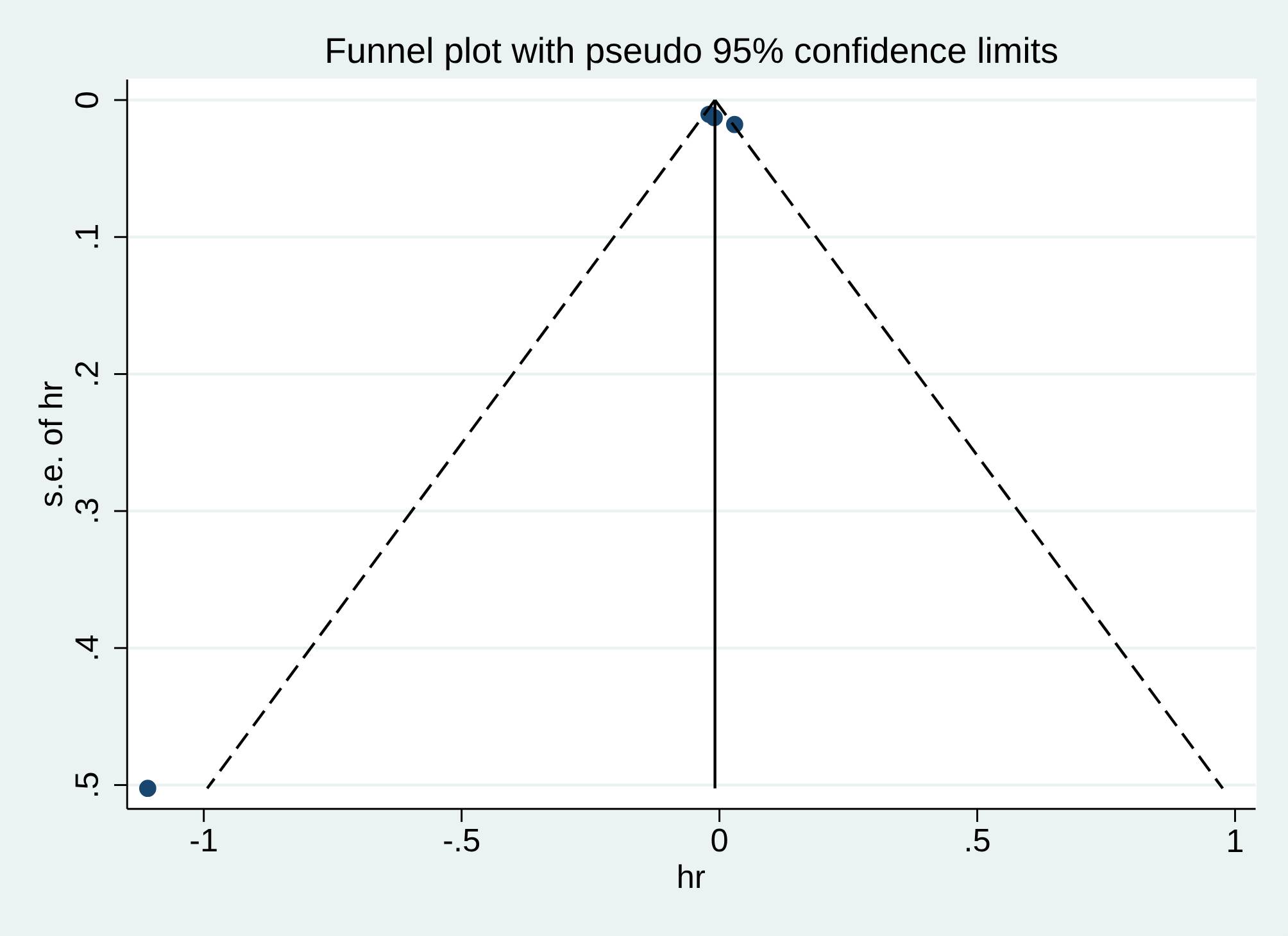
Supplementary Figure 5.**The funnel plot for LVEF(SMD).

**
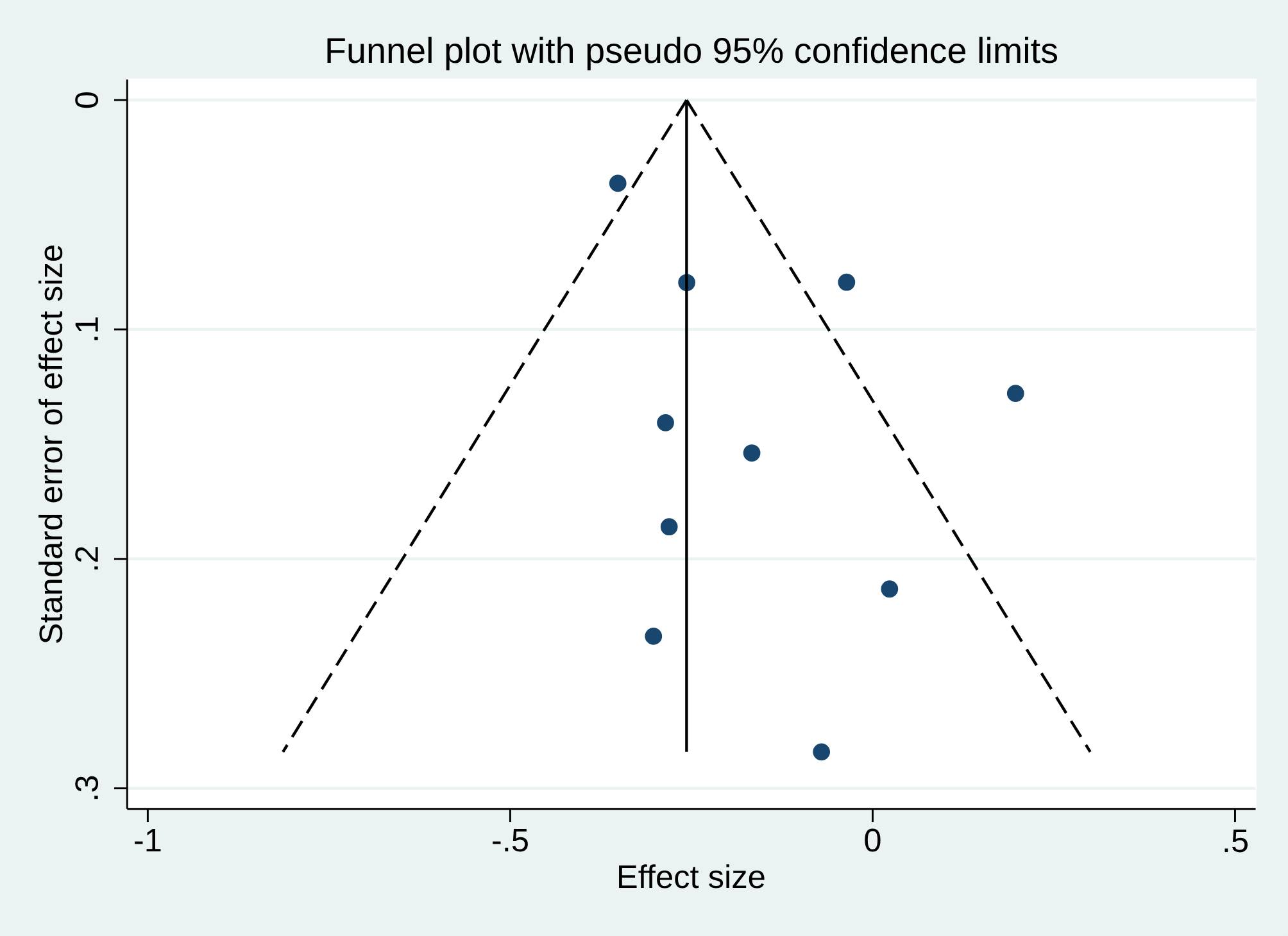
Supplementary Figure 6.**The funnel plot for RV FAC.

**
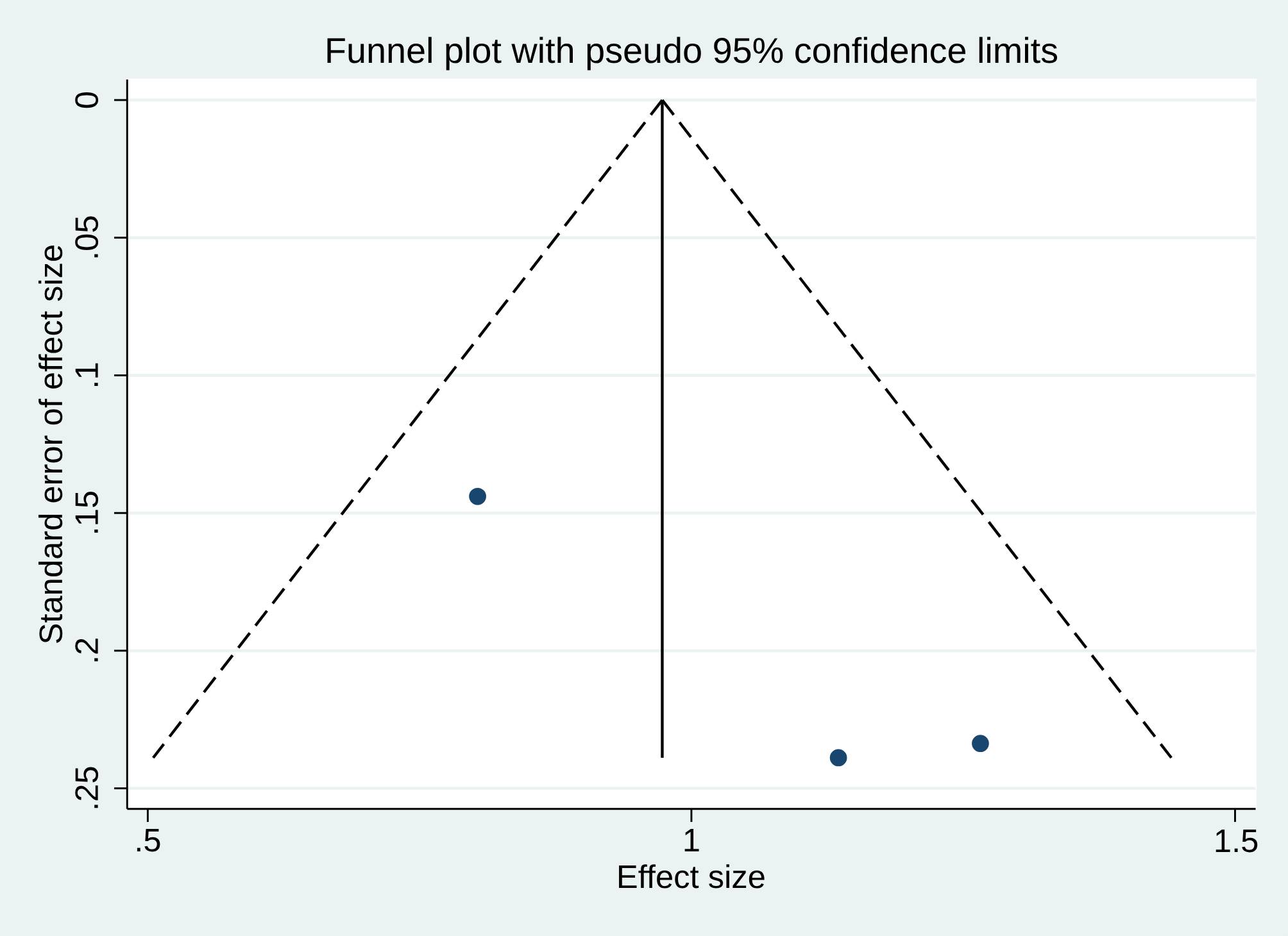
Supplementary Figure 7.**The funnel plot for SPAP.

**
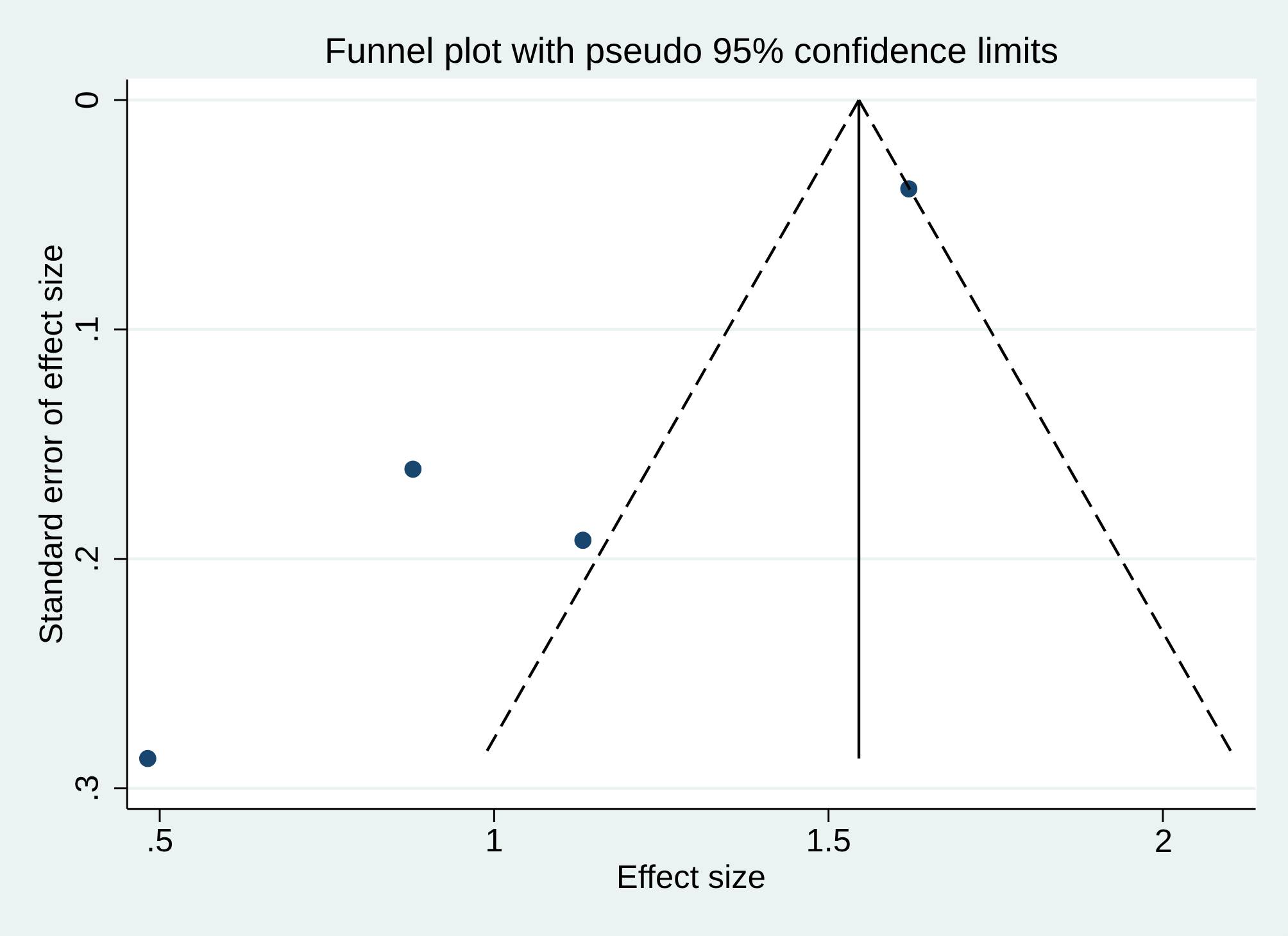
Supplementary Figure 8.**The funnel plot for RVSP.

**
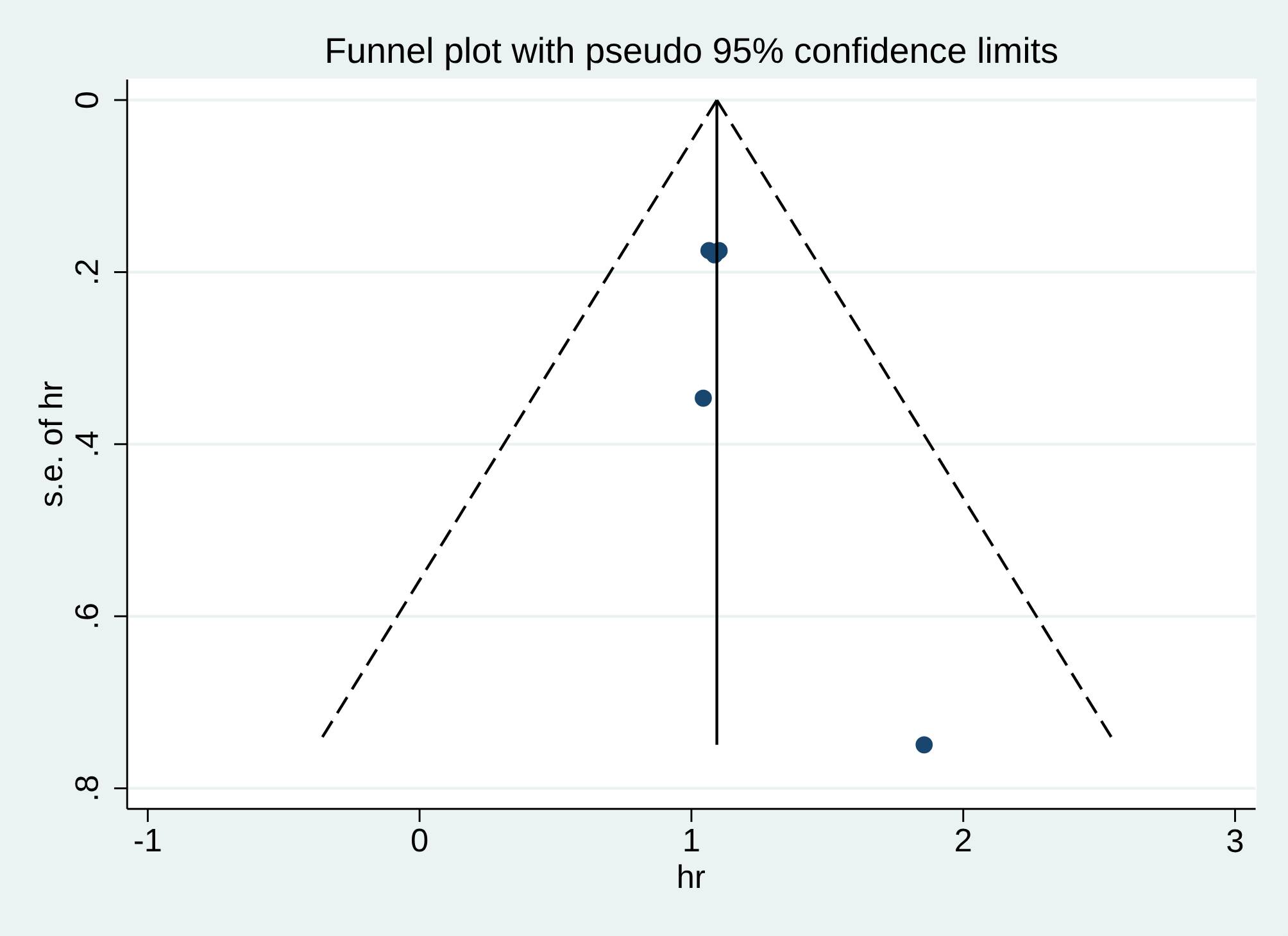
Supplementary Figure 9.**The funnel plot for PAF

**
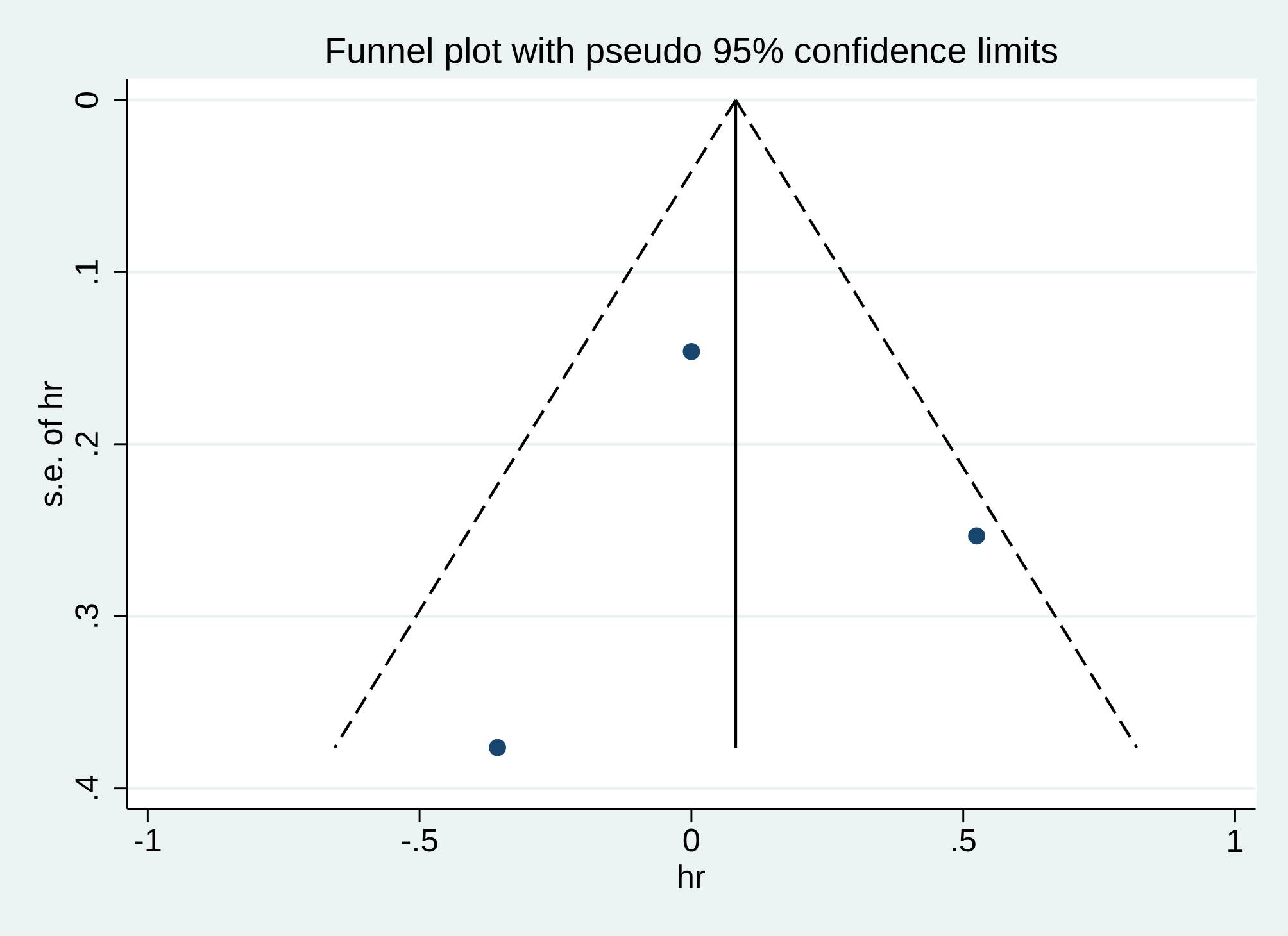
Supplementary Figure 10.**The funnel plot for CAD.

**
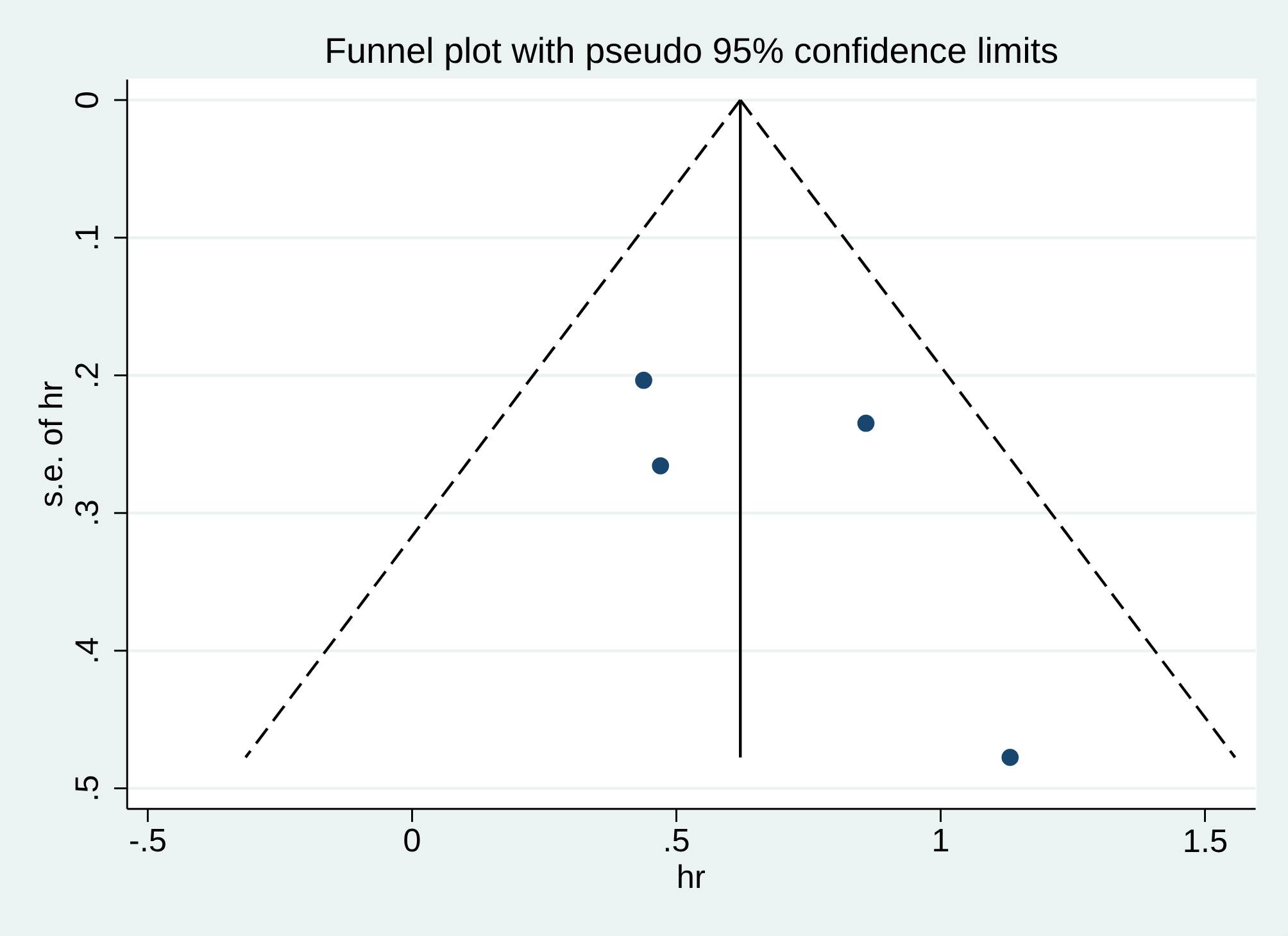
Supplementary Figure 11.**The funnel plot for HF.

**
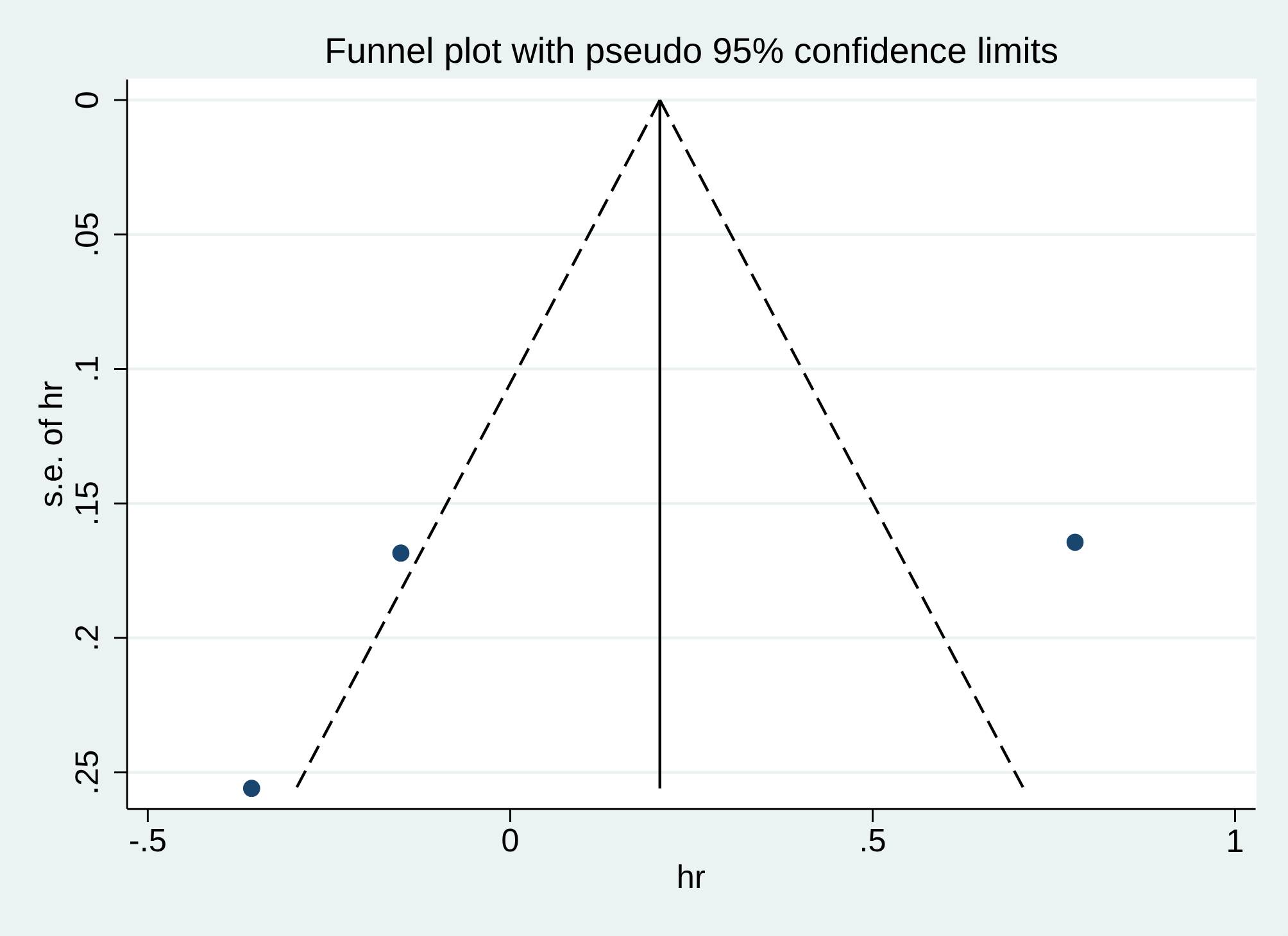
Supplementary Figure 12.**The funnel plot for CKD.

**
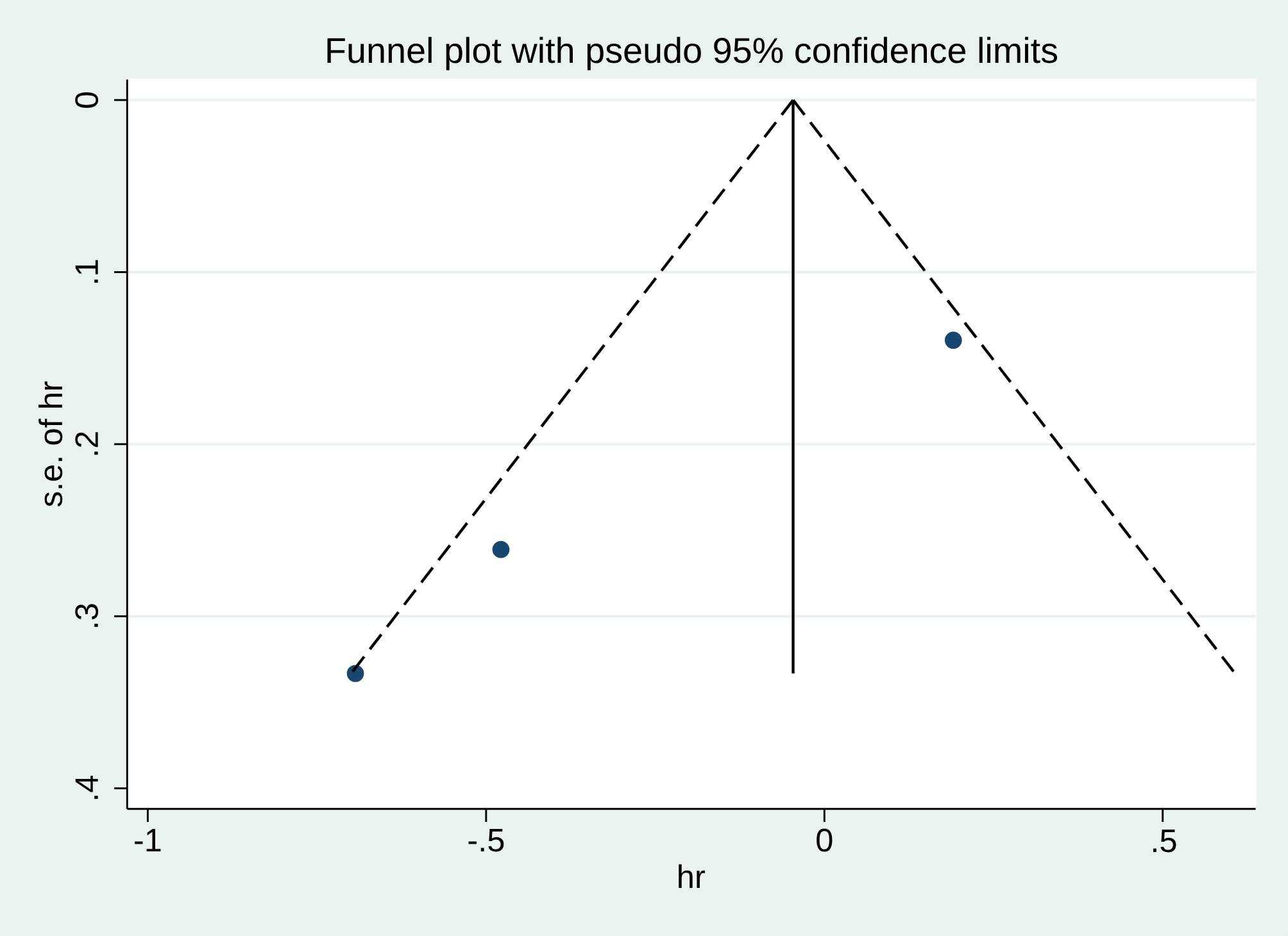
Supplementary Figure 13.**The funnel plot for DM.

**
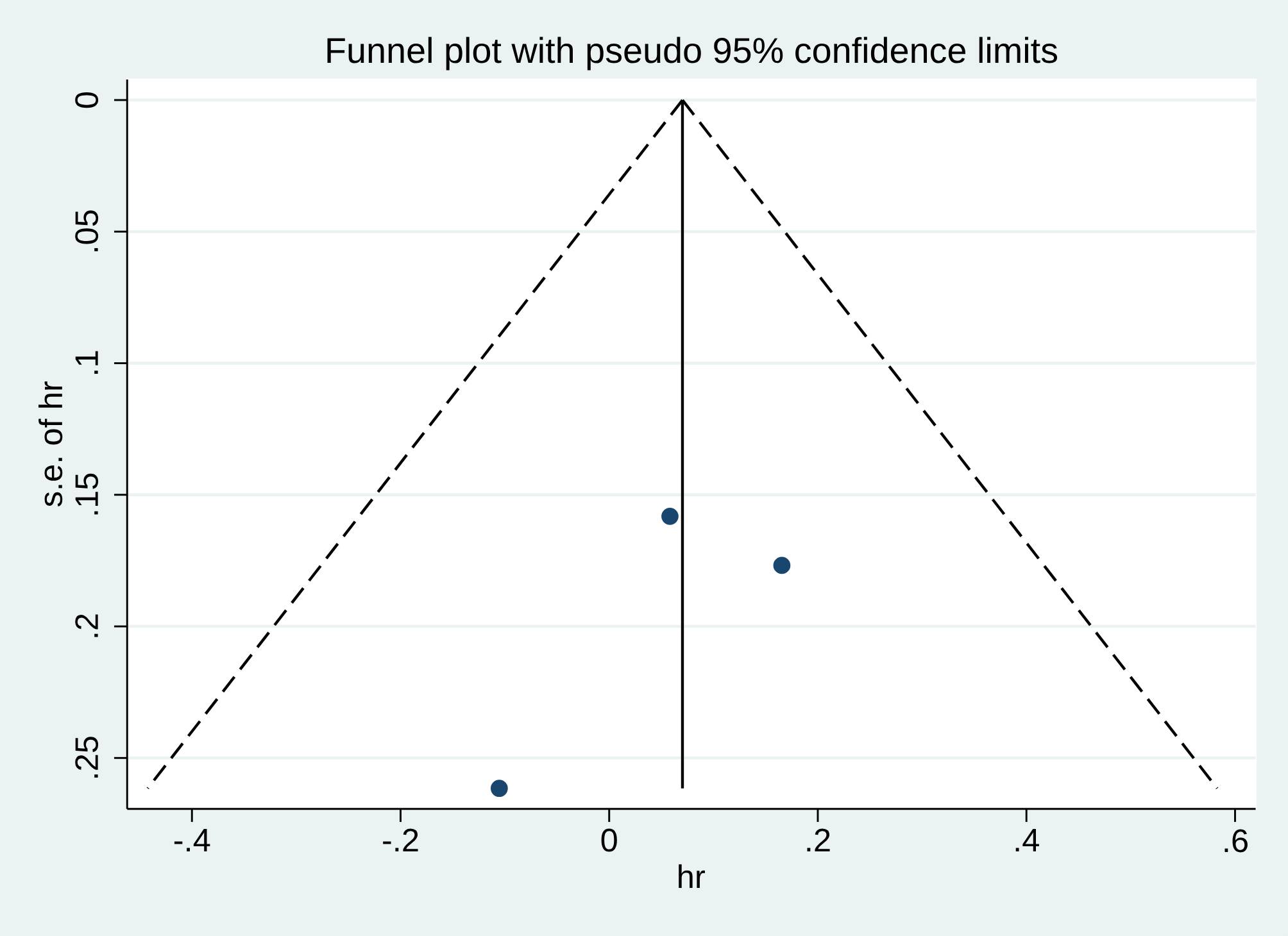
Supplementary Figure 14.**The funnel plot for hypertension.

**
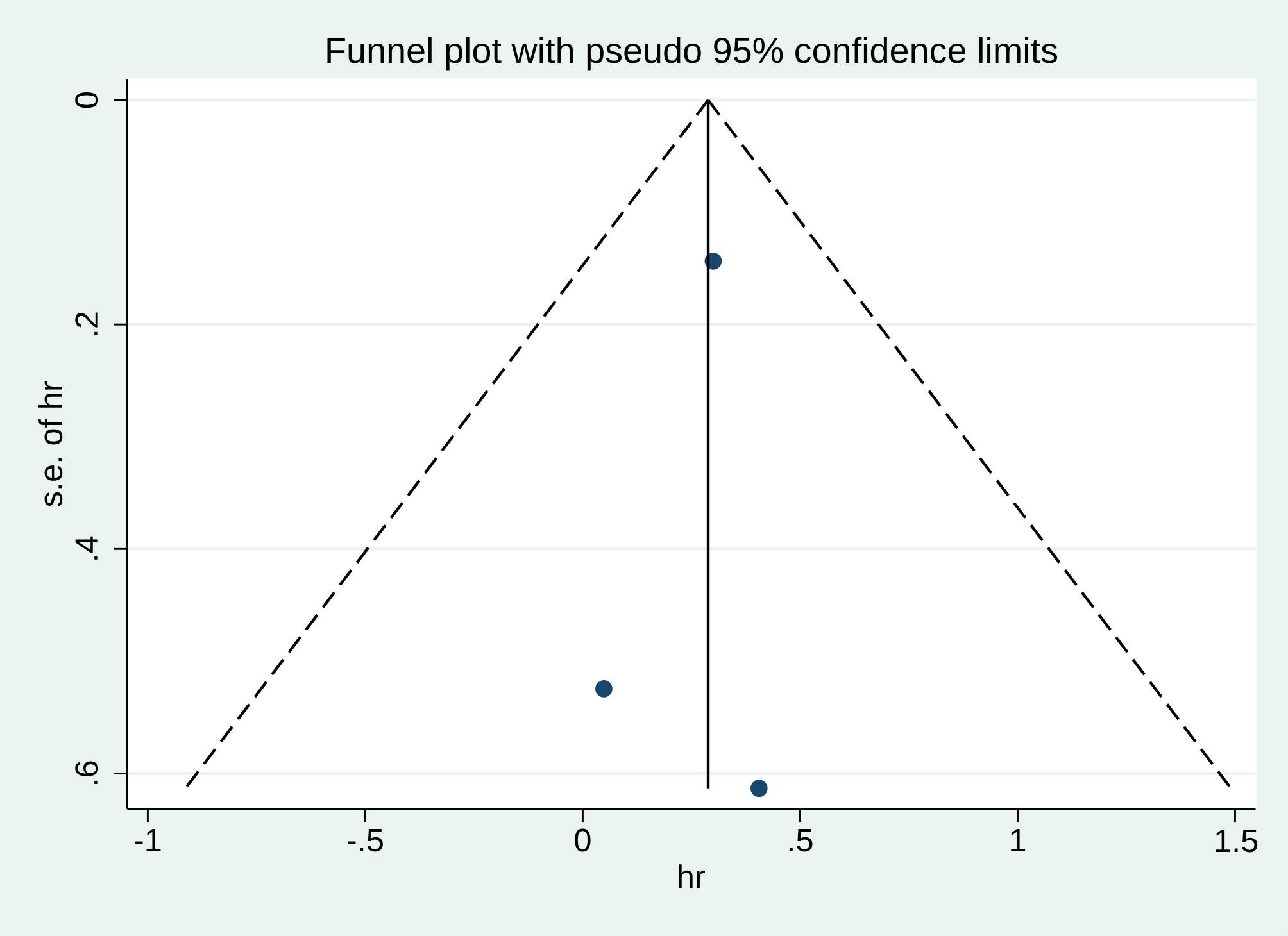
Supplementary Figure 15.**The funnel plot for CLD.

**
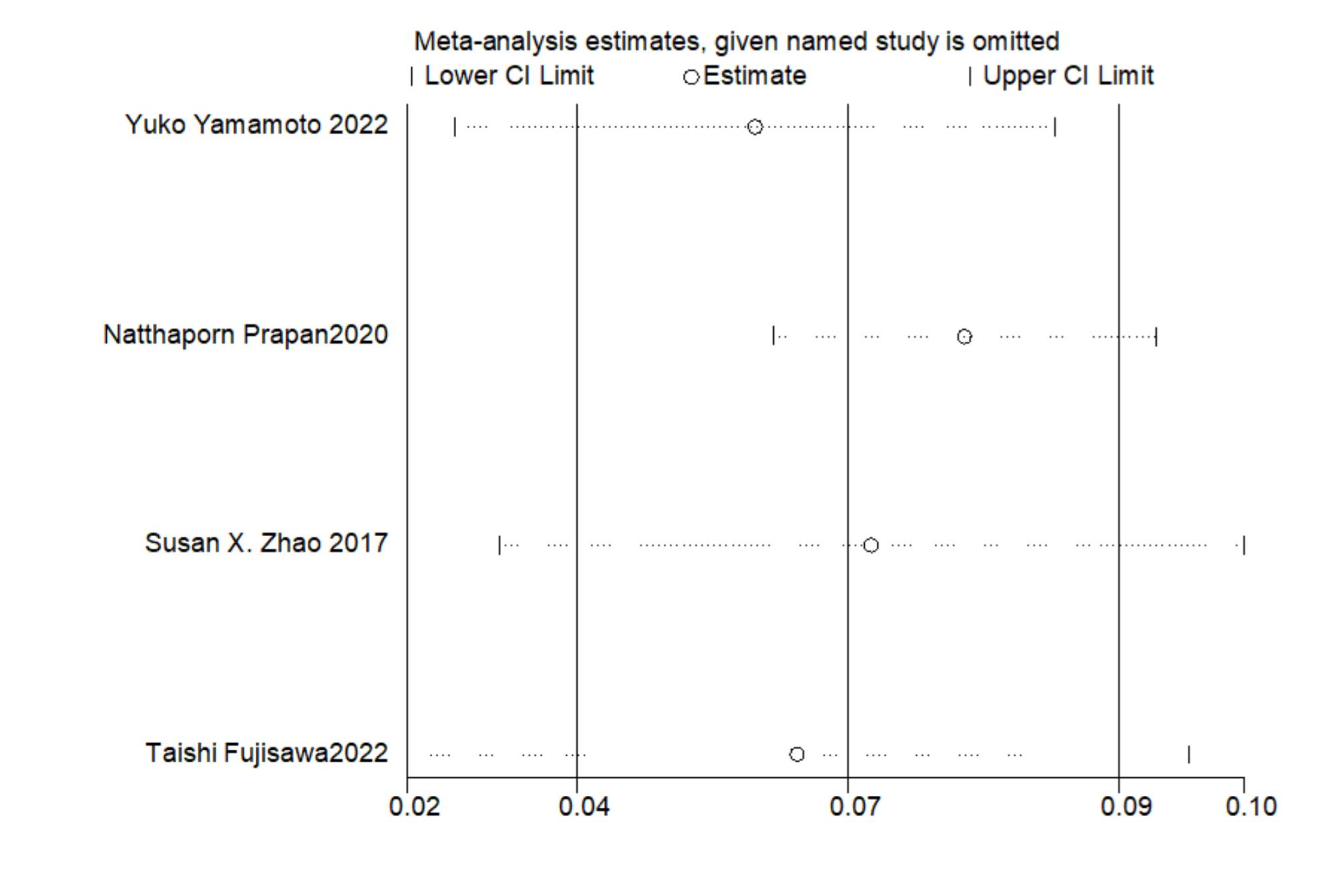
Supplementary Figure 16.**Sensitivity analysis of Age

**
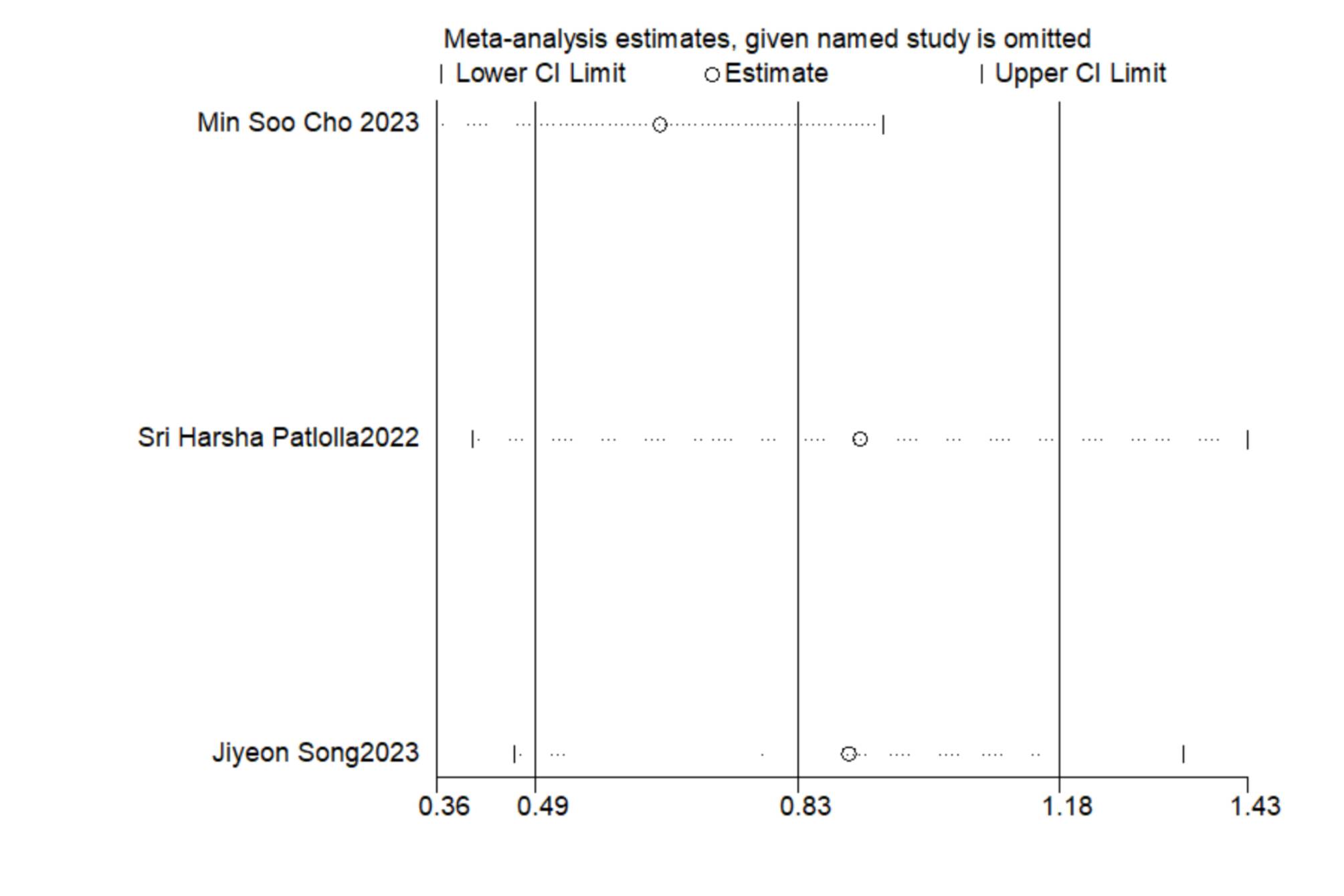
Supplementary Figure 17.**Sensitivity analysis of Age ≥ 65 years.

**
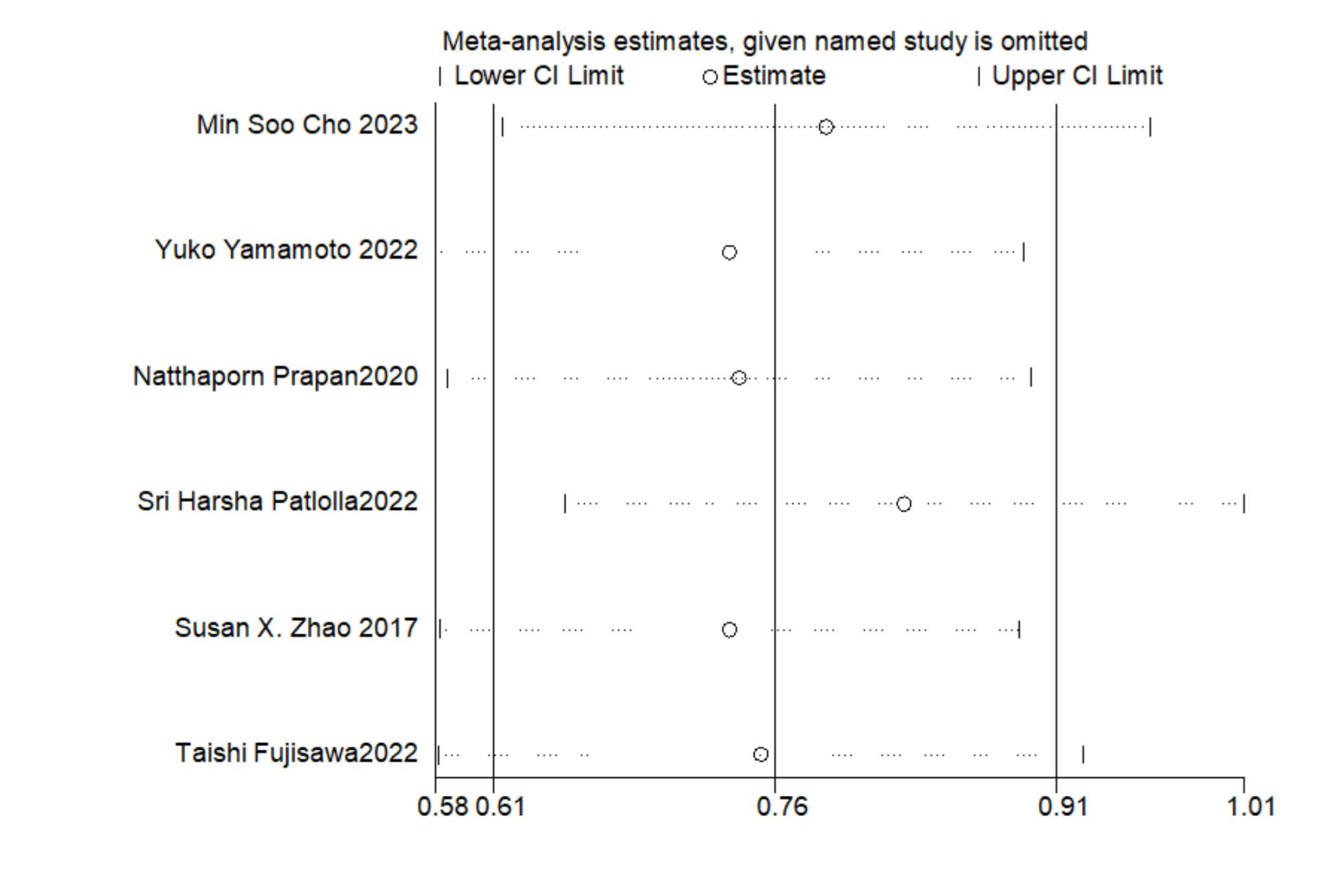
Supplementary Figure 18.**Sensitivity analysis of Female

**
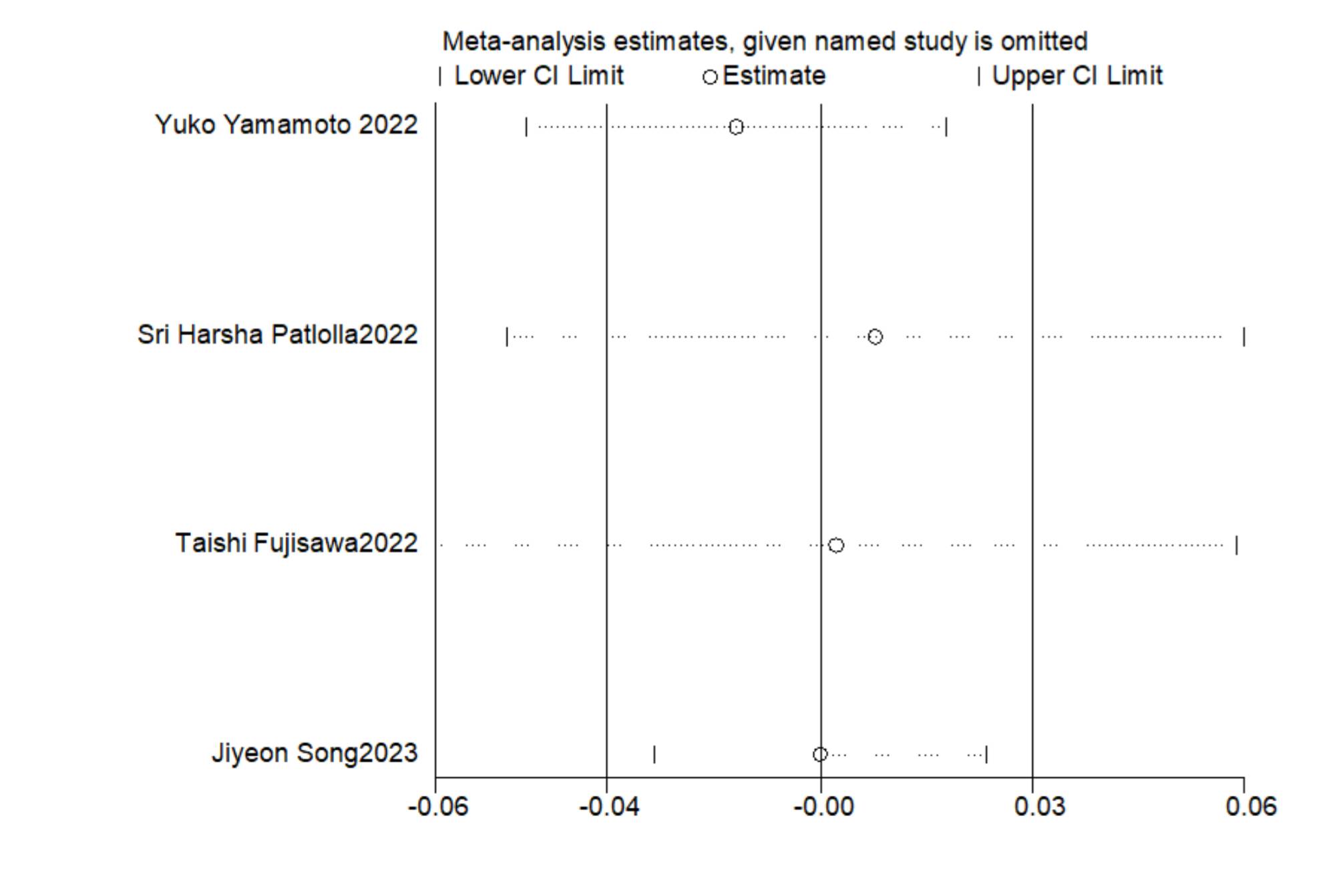
Supplementary Figure 19.**Sensitivity analysis of LVEF(HR)

**
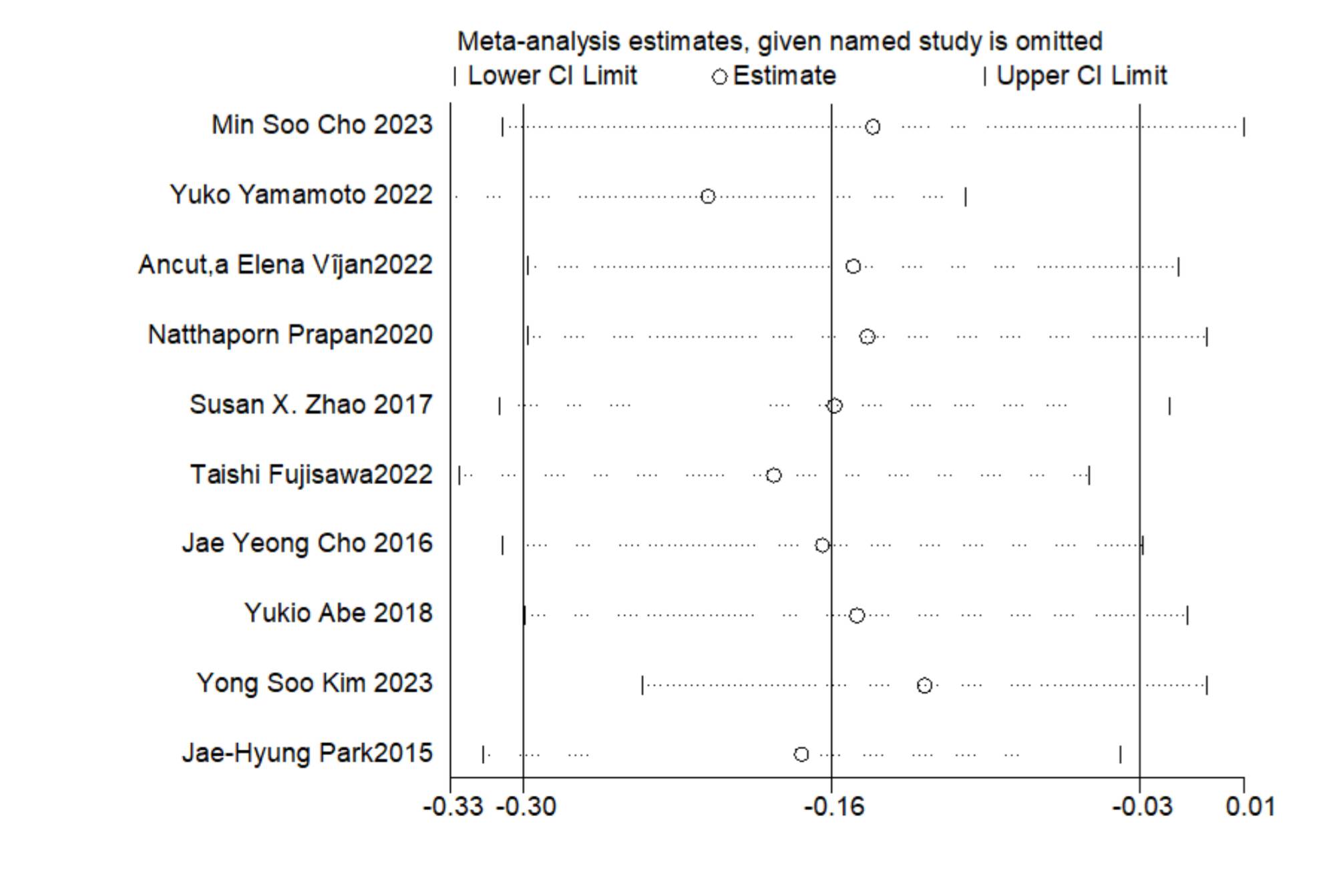
Supplementary Figure 20.**Sensitivity analysis of LVEF(SMD).

**
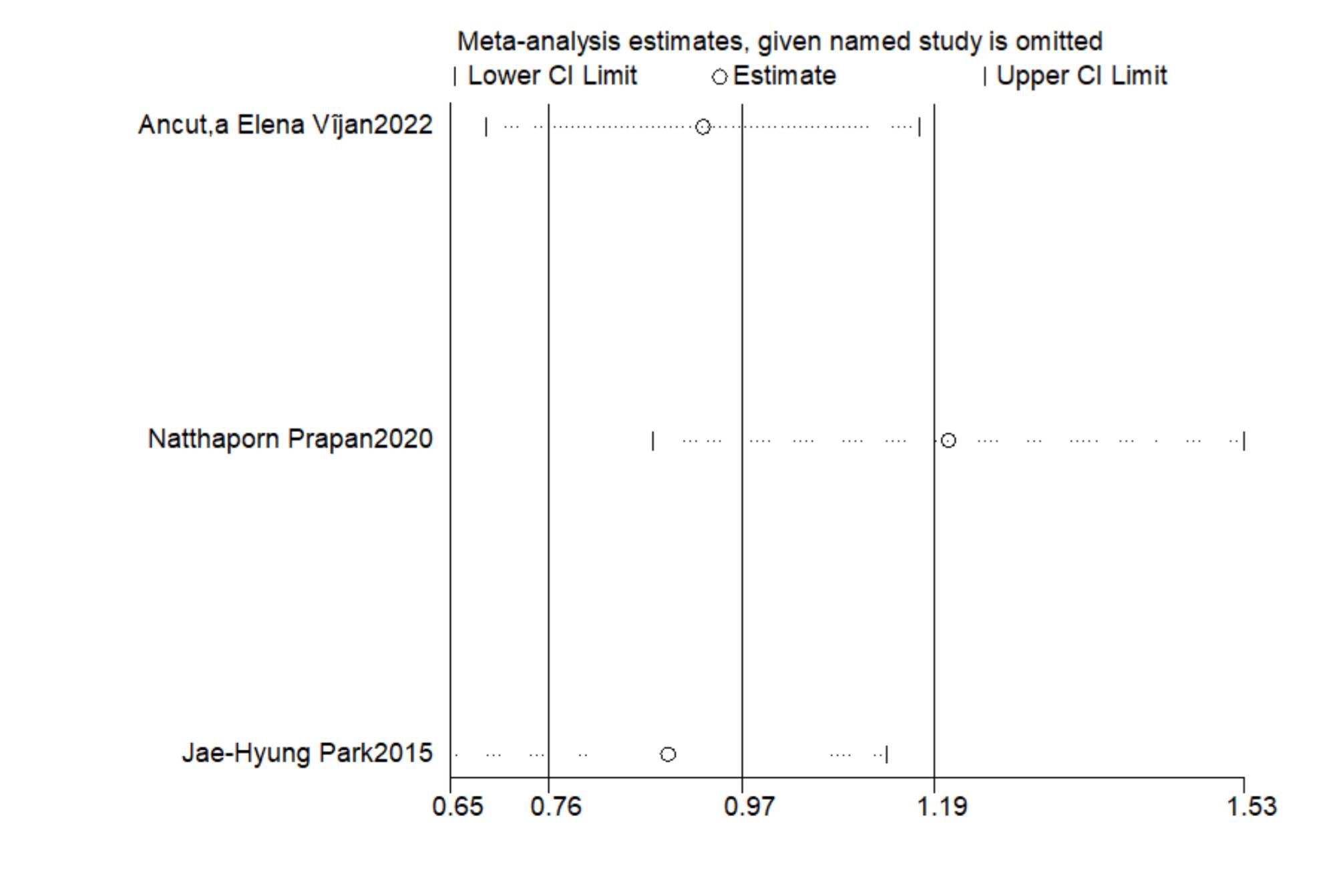
Supplementary Figure 21.**Sensitivity analysis of RV FAC.

**
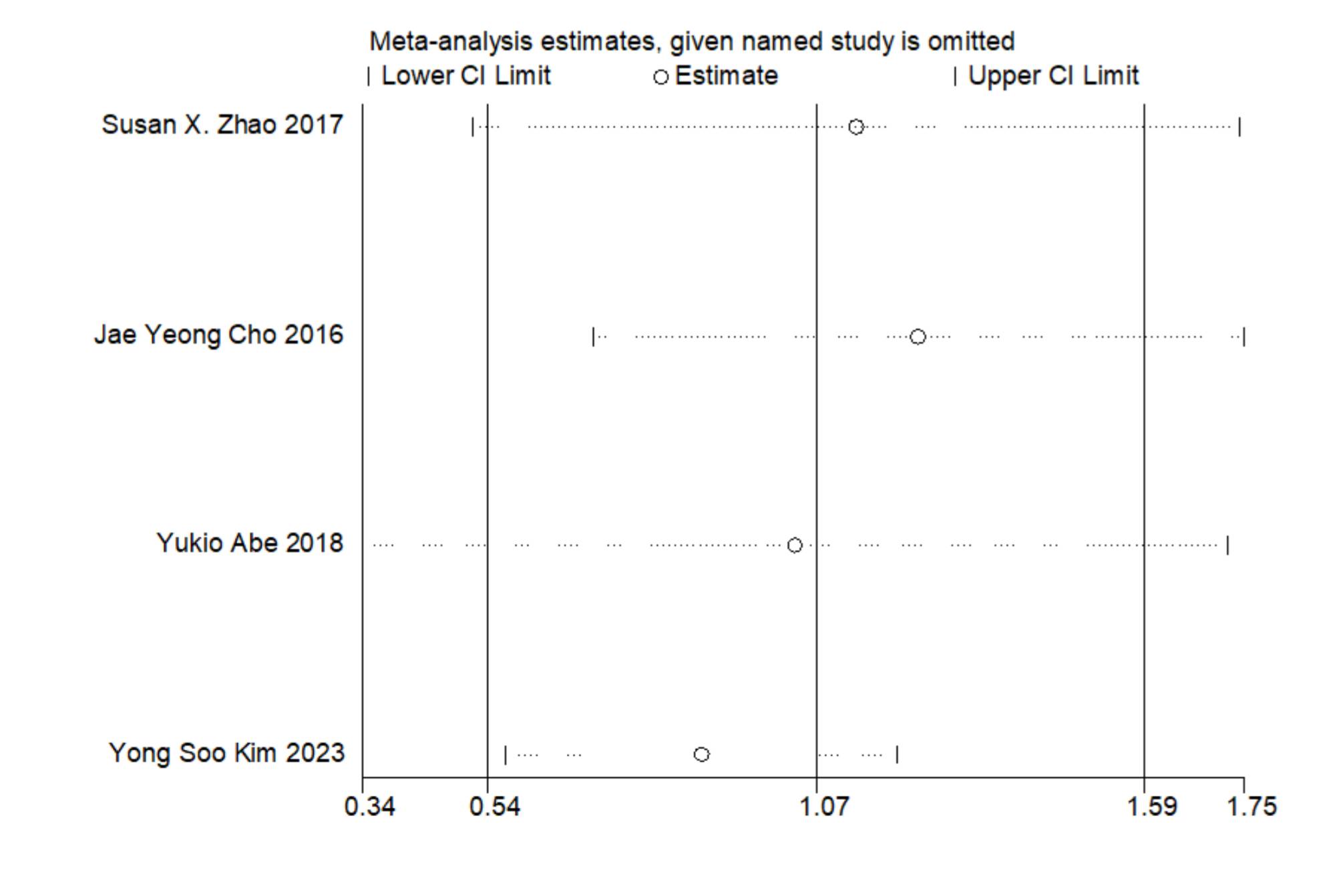
Supplementary Figure 22.**Sensitivity analysis of SPAP.

**
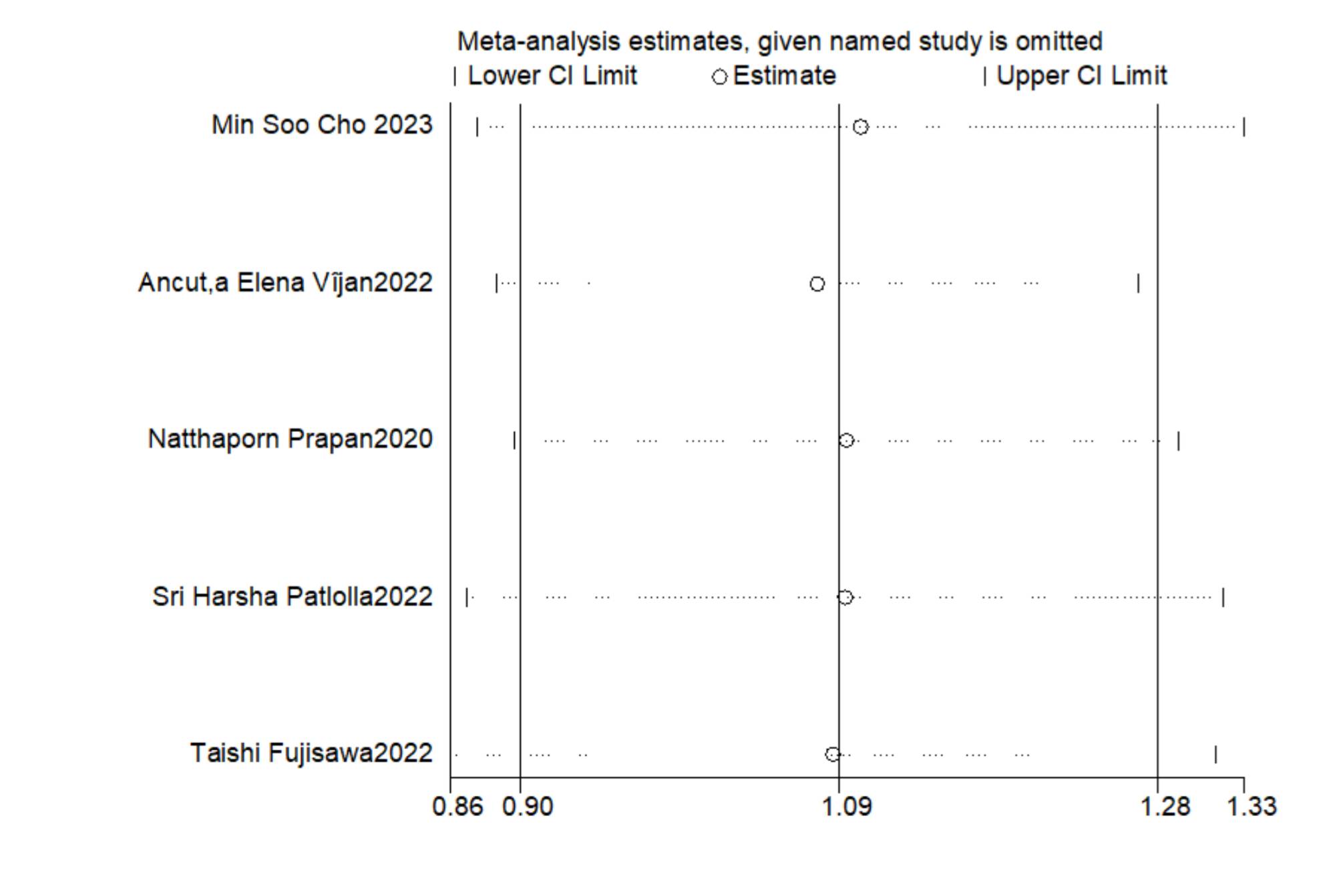
Supplementary Figure 23.**Sensitivity analysis of RVSP.

**
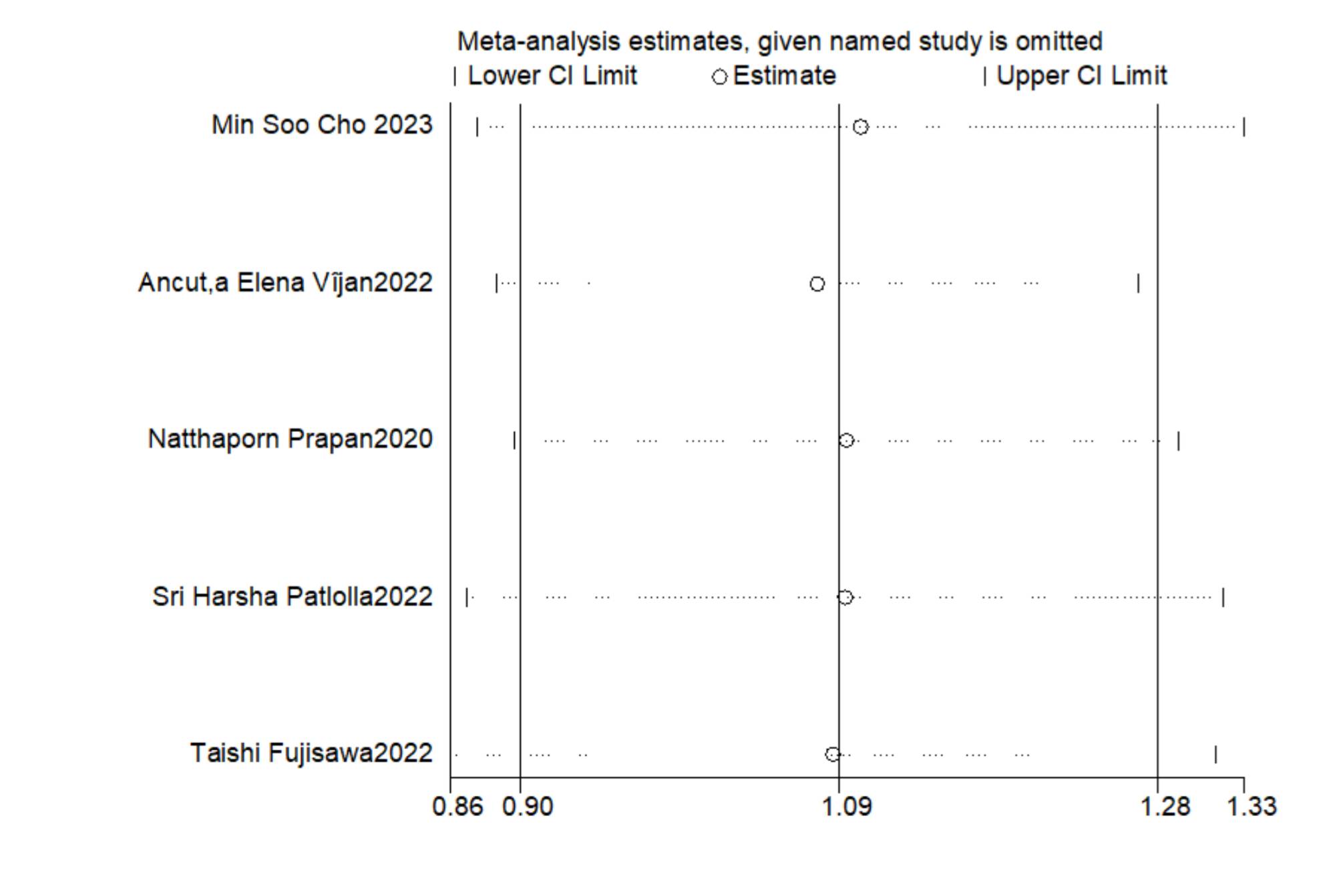
Supplementary Figure 24.**Sensitivity analysis of PAF

**
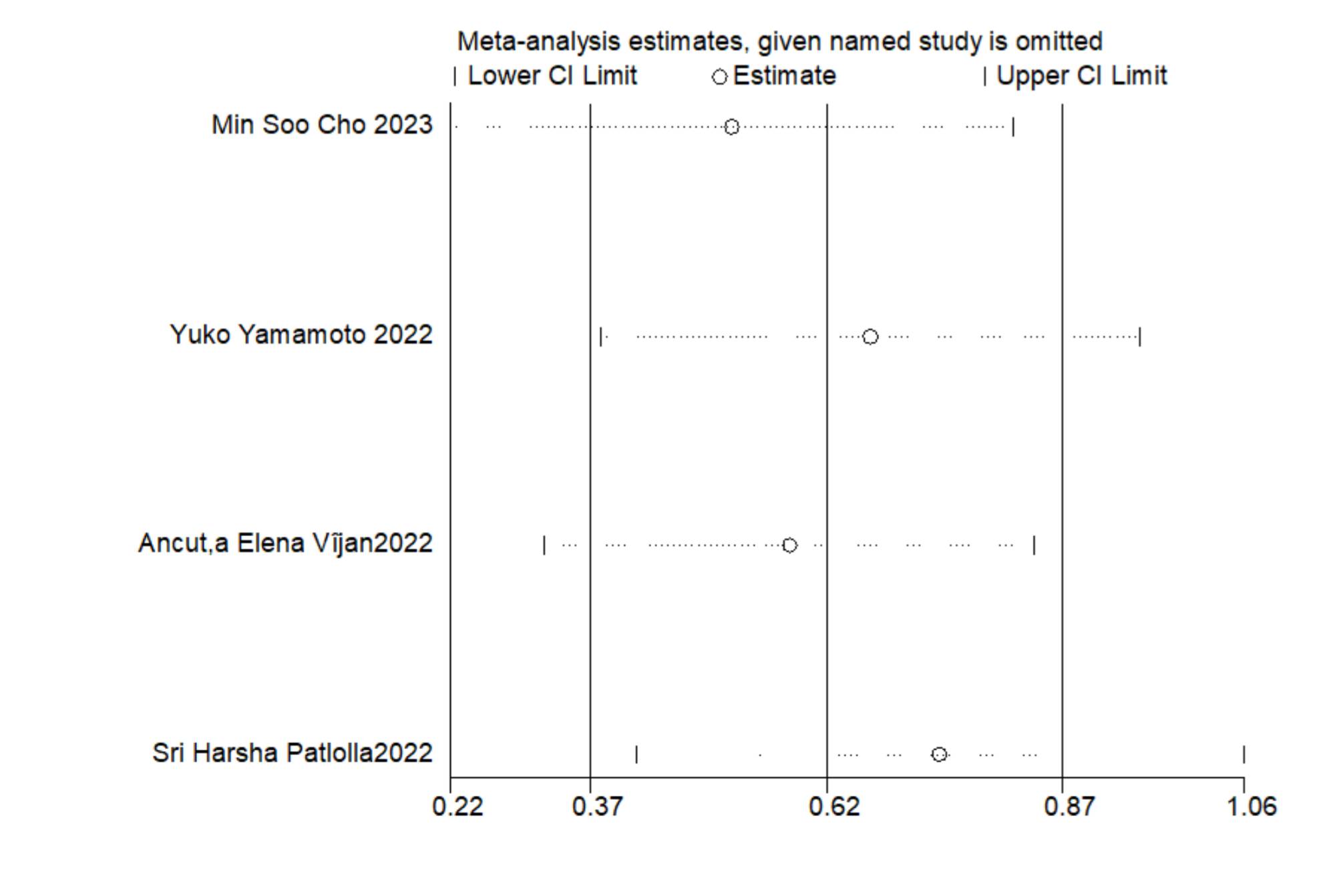
Supplementary Figure 25.**Sensitivity analysis of CAD.

**
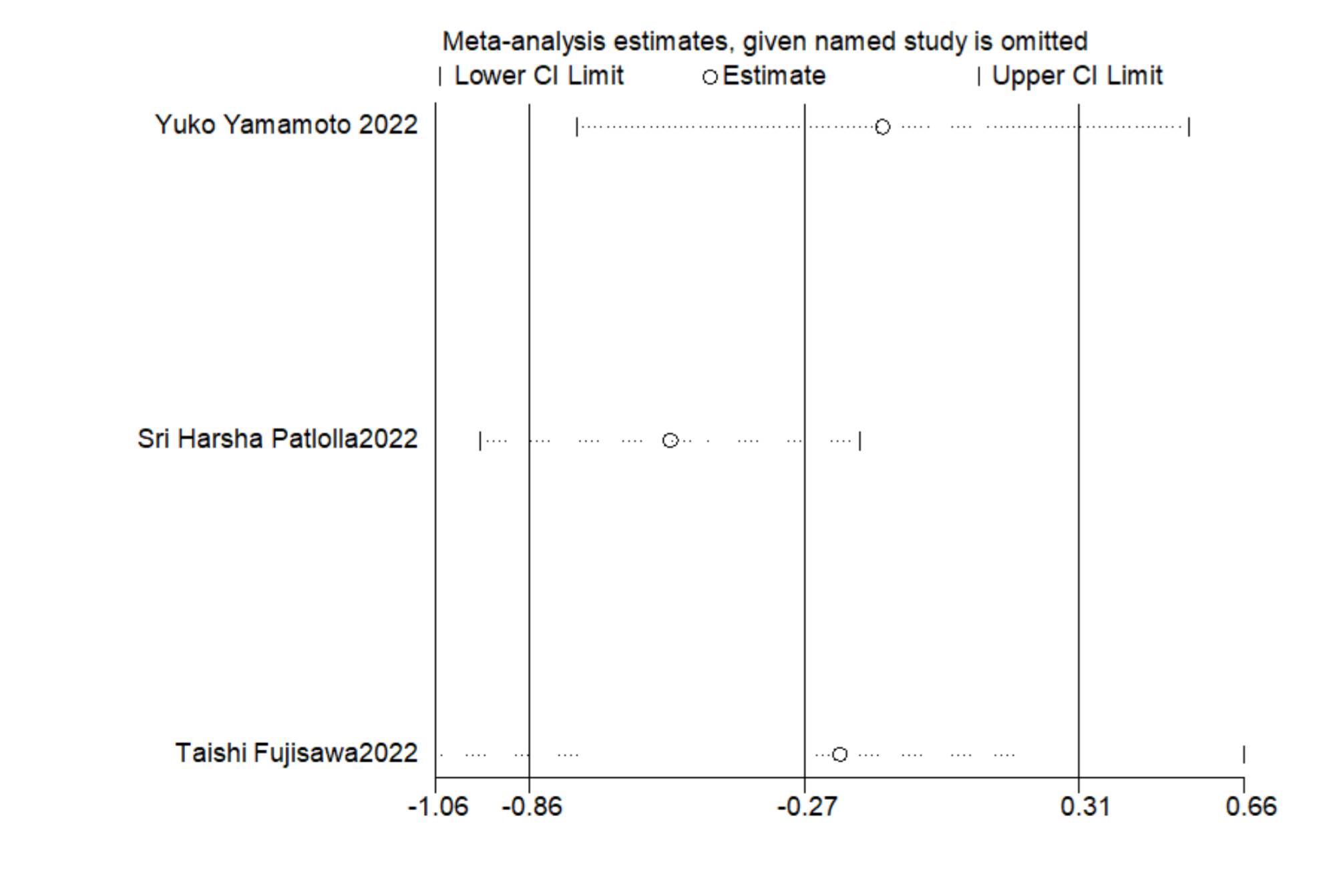
Supplementary Figure 26.**Sensitivity analysis of HF.

**
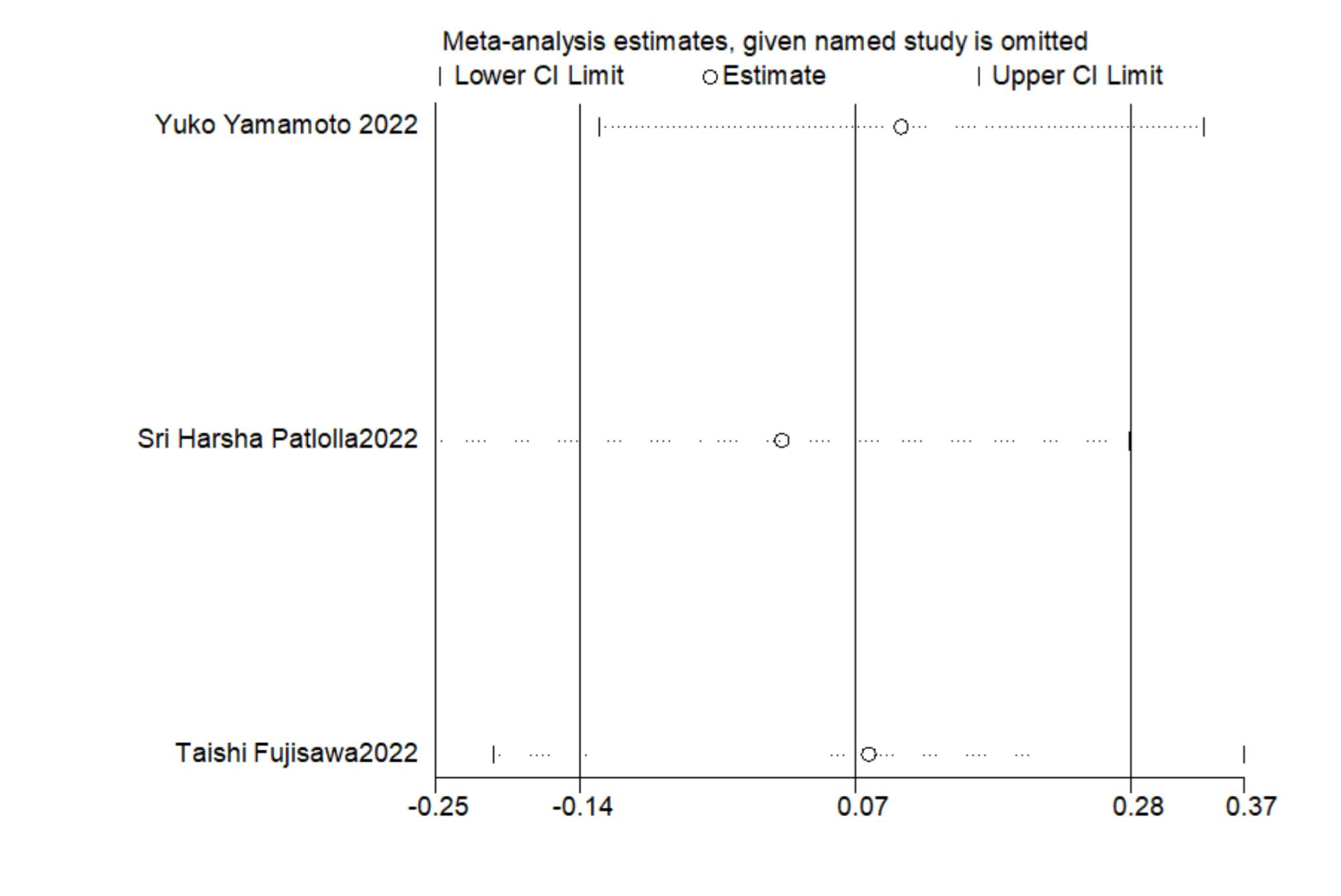
Supplementary Figure 27.**Sensitivity analysis of CKD.

**
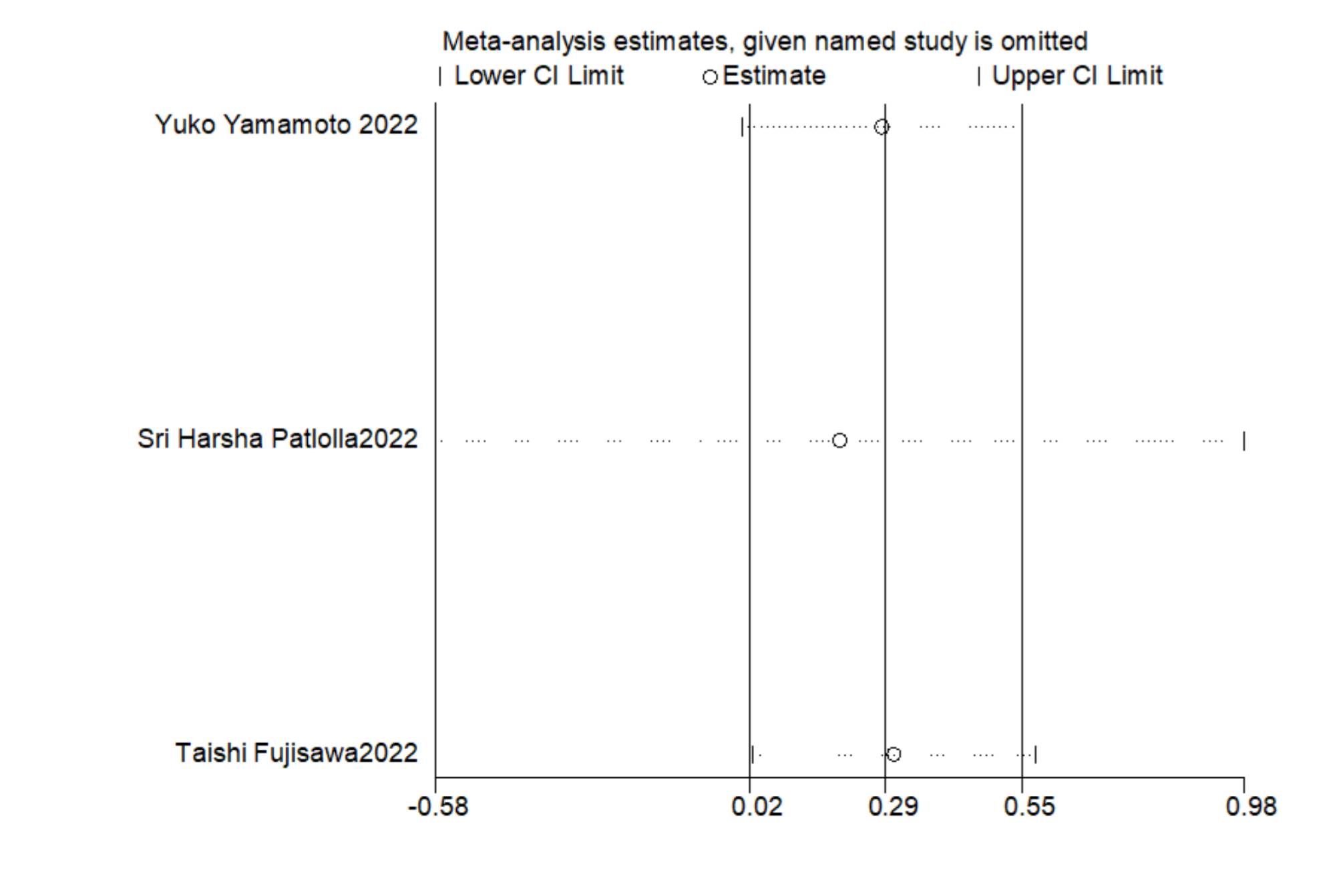
Supplementary Figure 28.**Sensitivity analysis of DM.

**
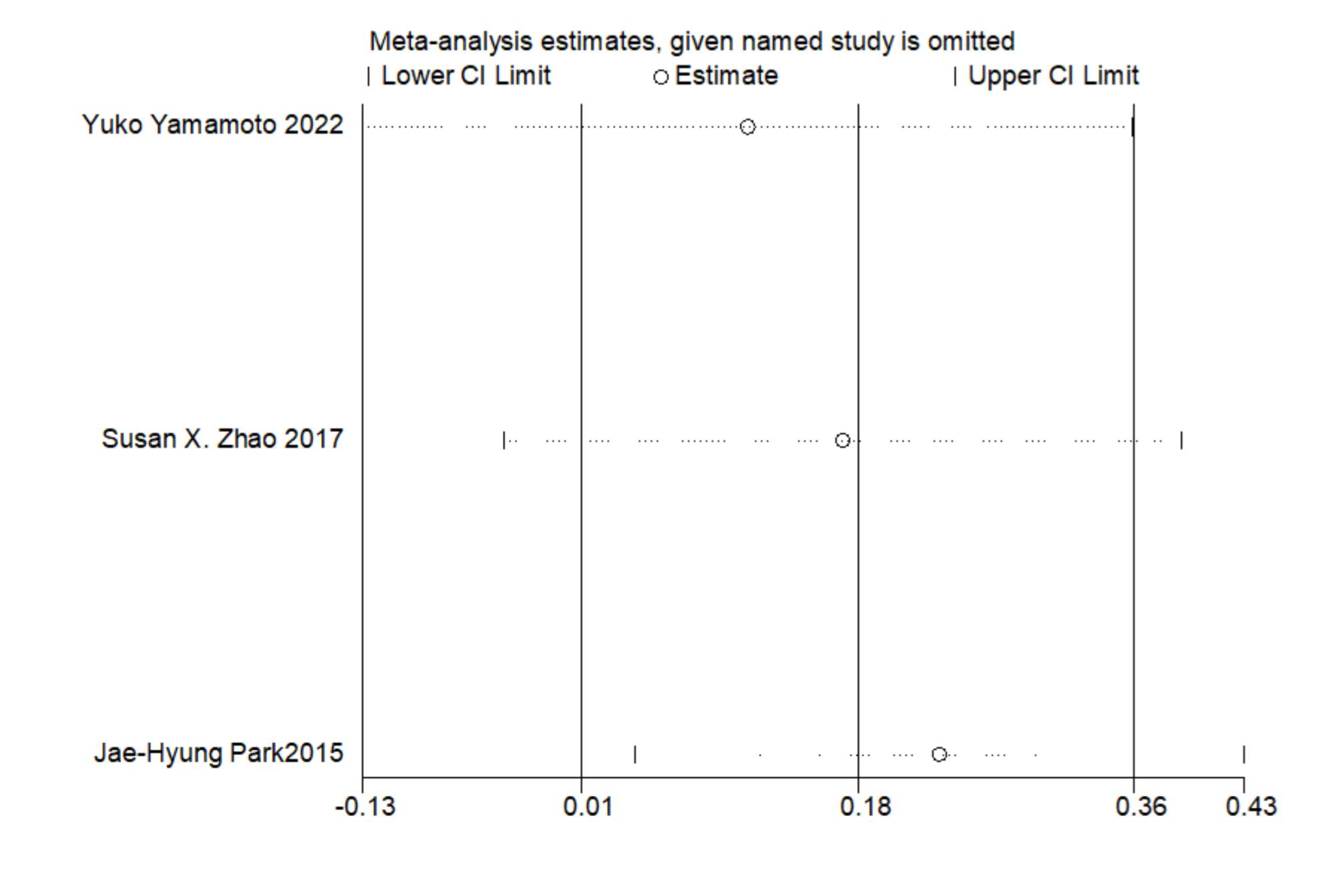
Supplementary Figure 29.**Sensitivity analysis of hypertension.

**
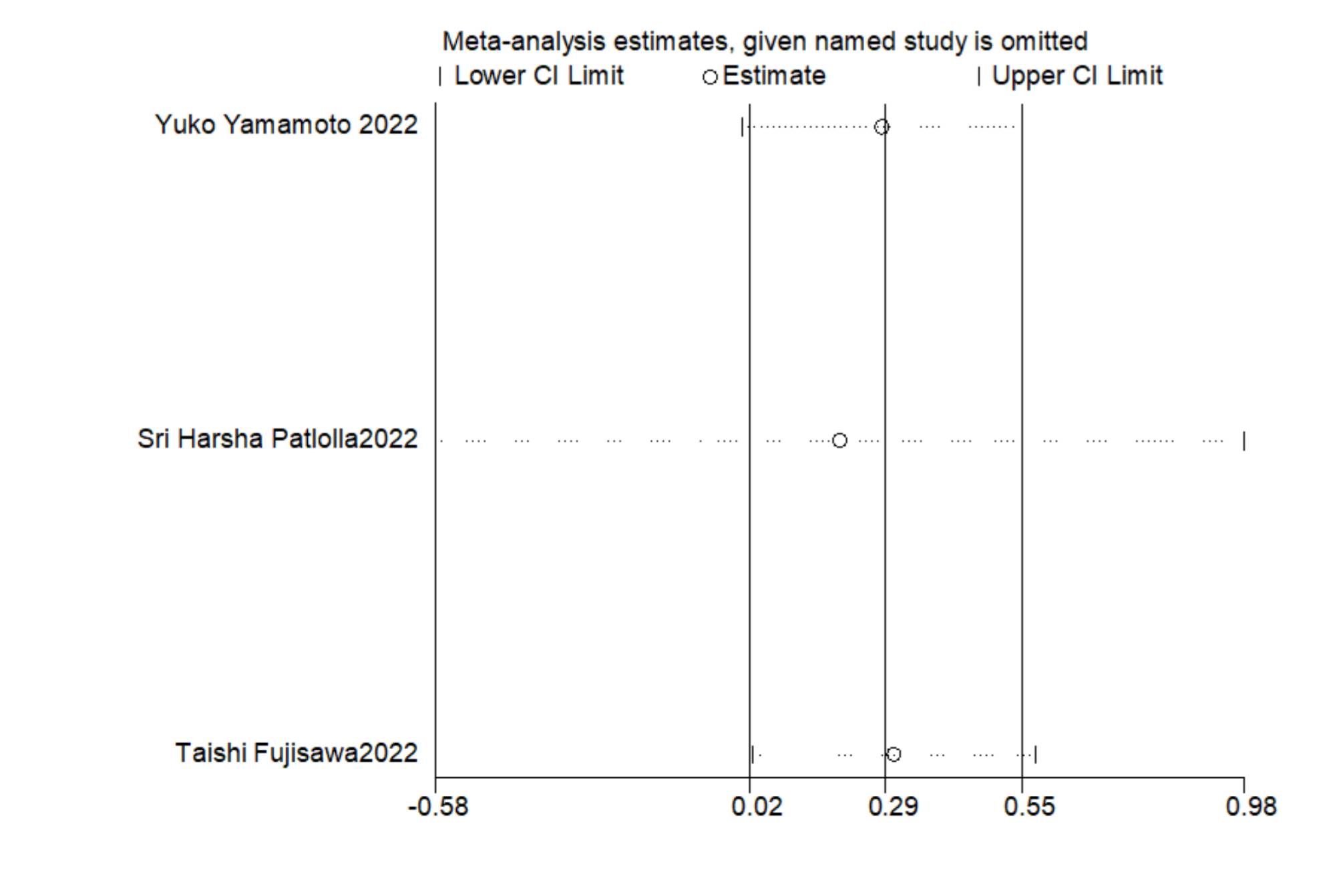
Supplementary Figure 30.**Sensitivity analysis of CLD.

**
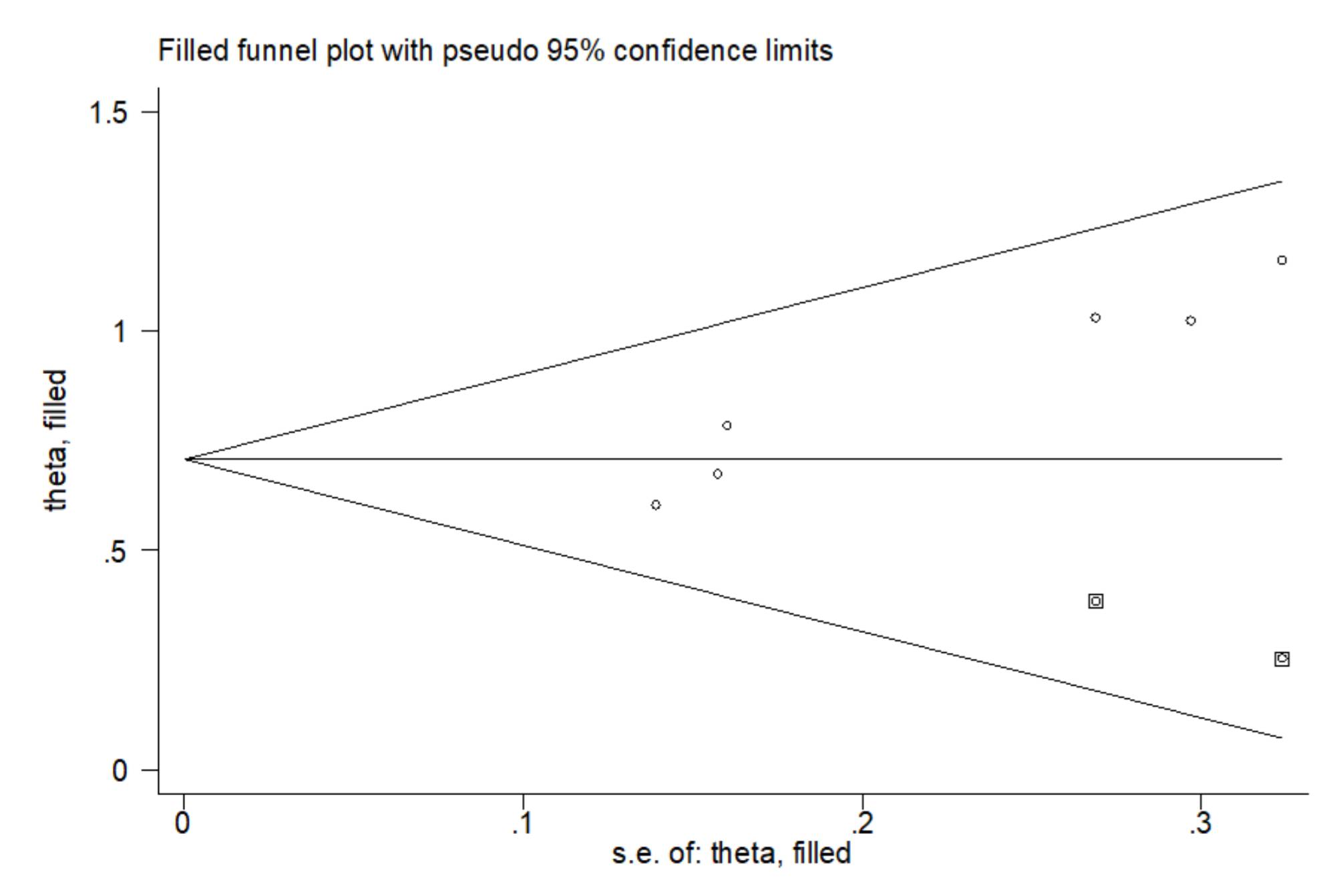
Supplementary Figure 31.**Trim and fill method analysis of Female

**
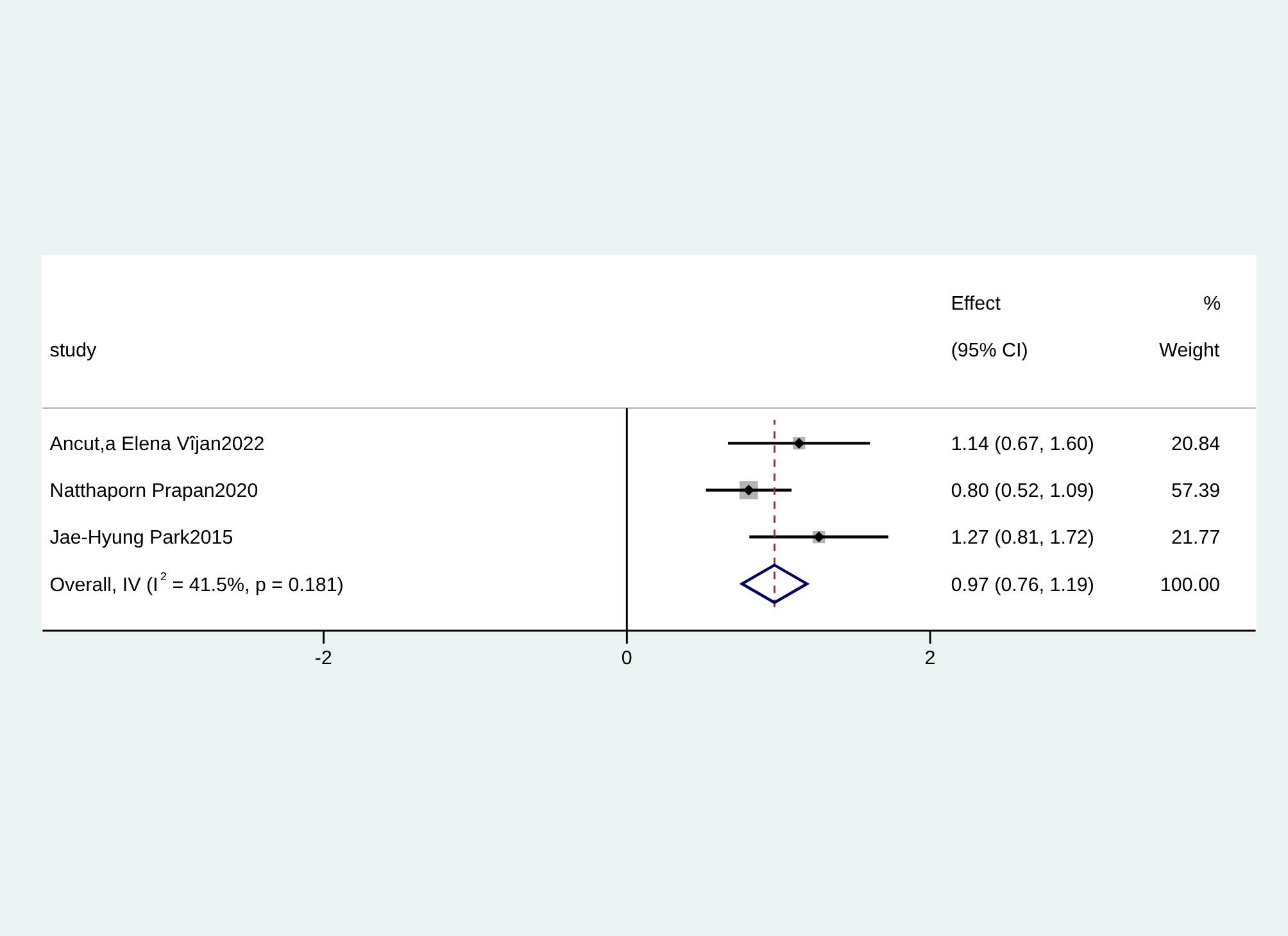
Supplementary Figure 32.**Forest plot for SPAP.

**
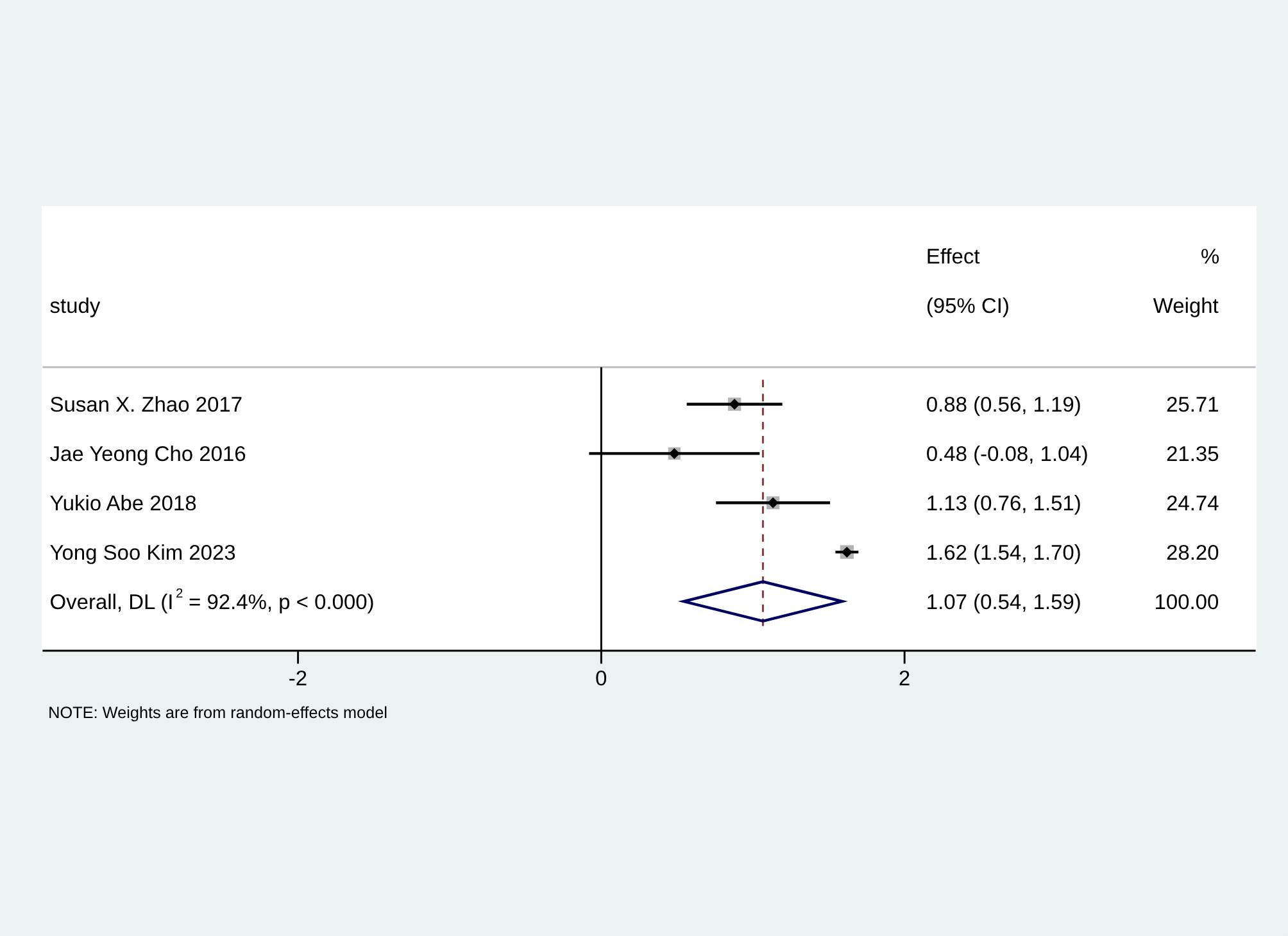
Supplementary Figure 33.**Forest plot for RVSP..

**
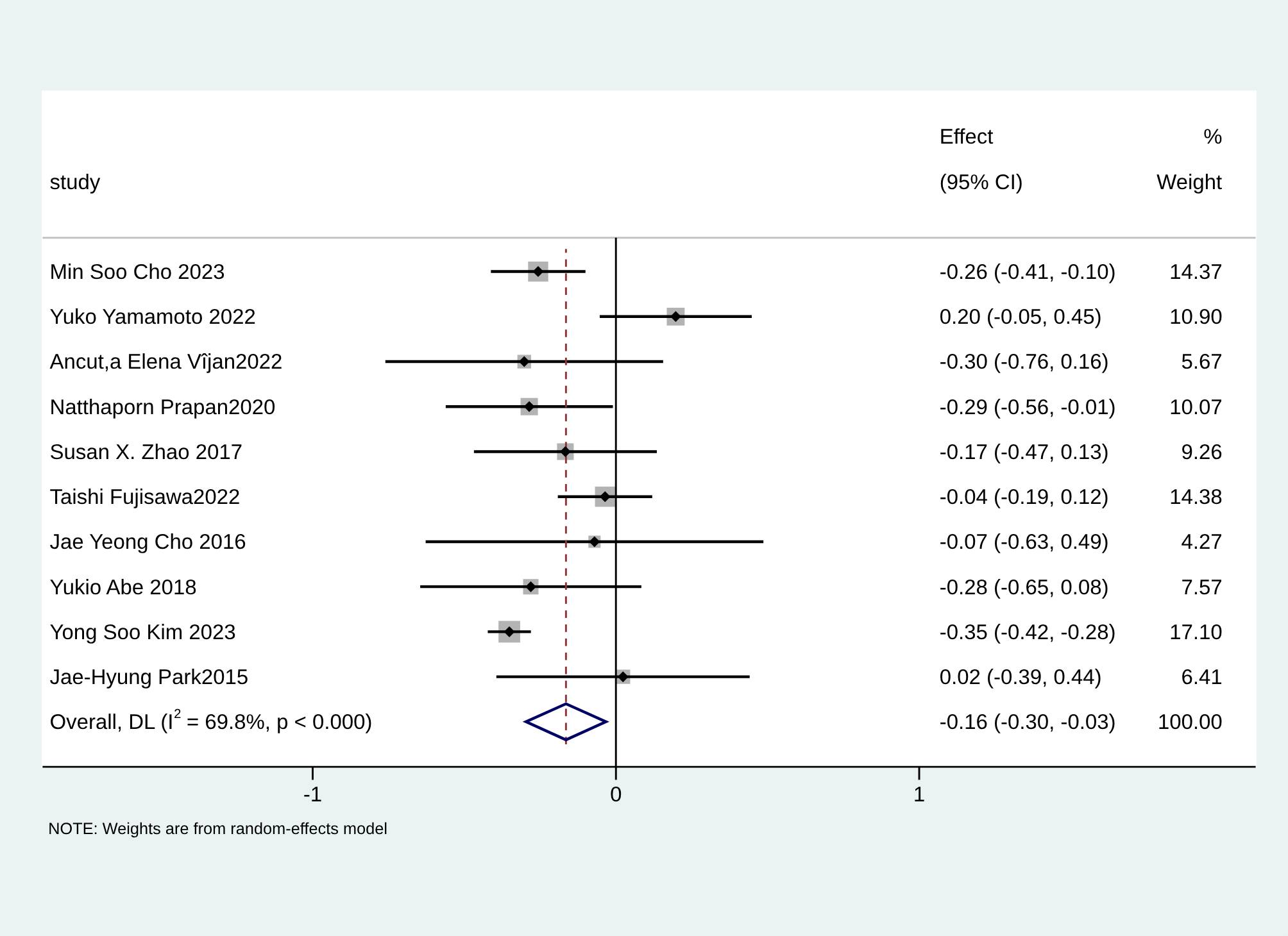
Supplementary Figure 34.**Forest plot for LVEF（SMD）..

**
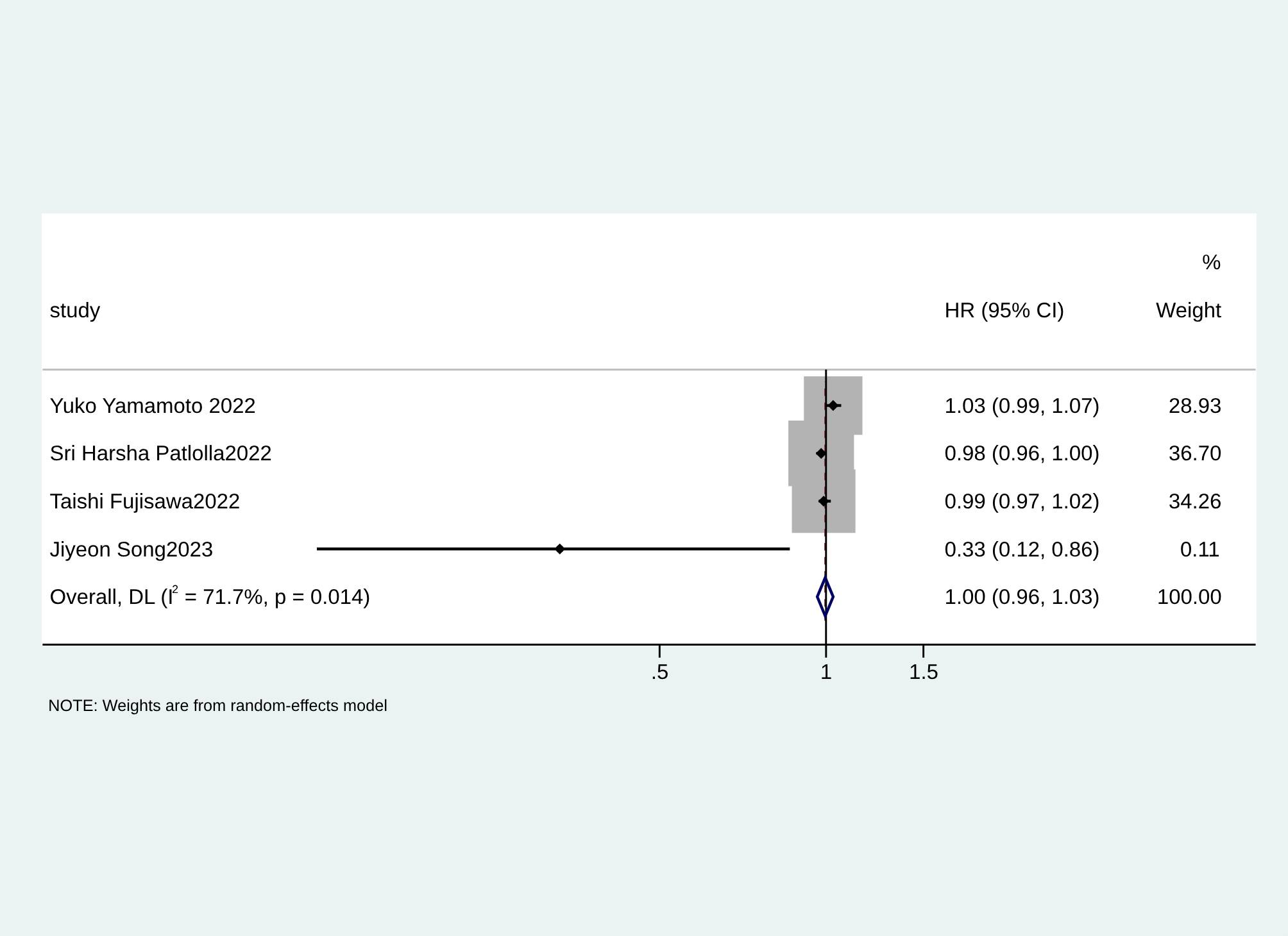
Supplementary Figure 35.**Forest plot for LVEF (HR).

**
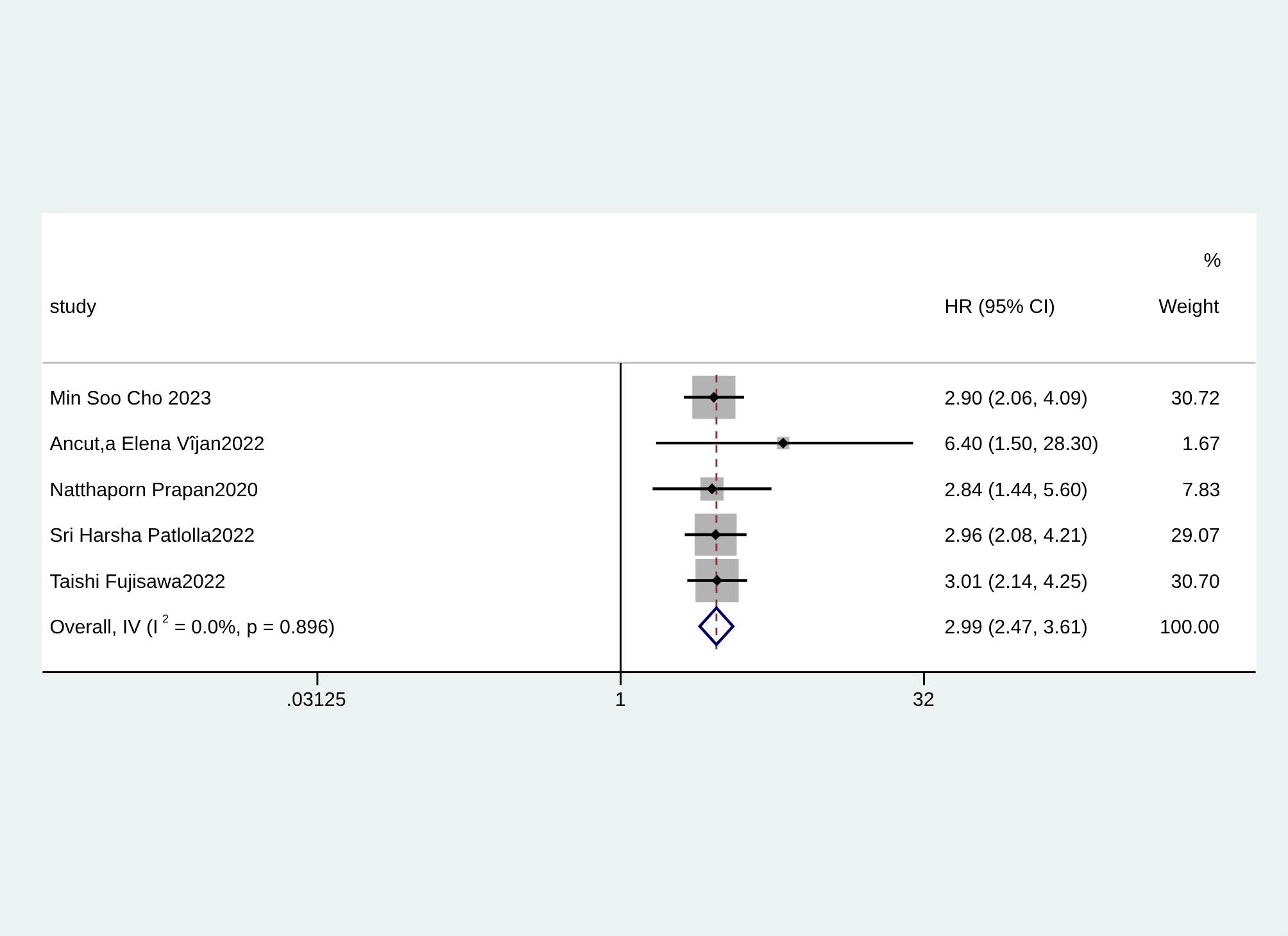
Supplementary Figure 36.**Forest plot for PAF.

**
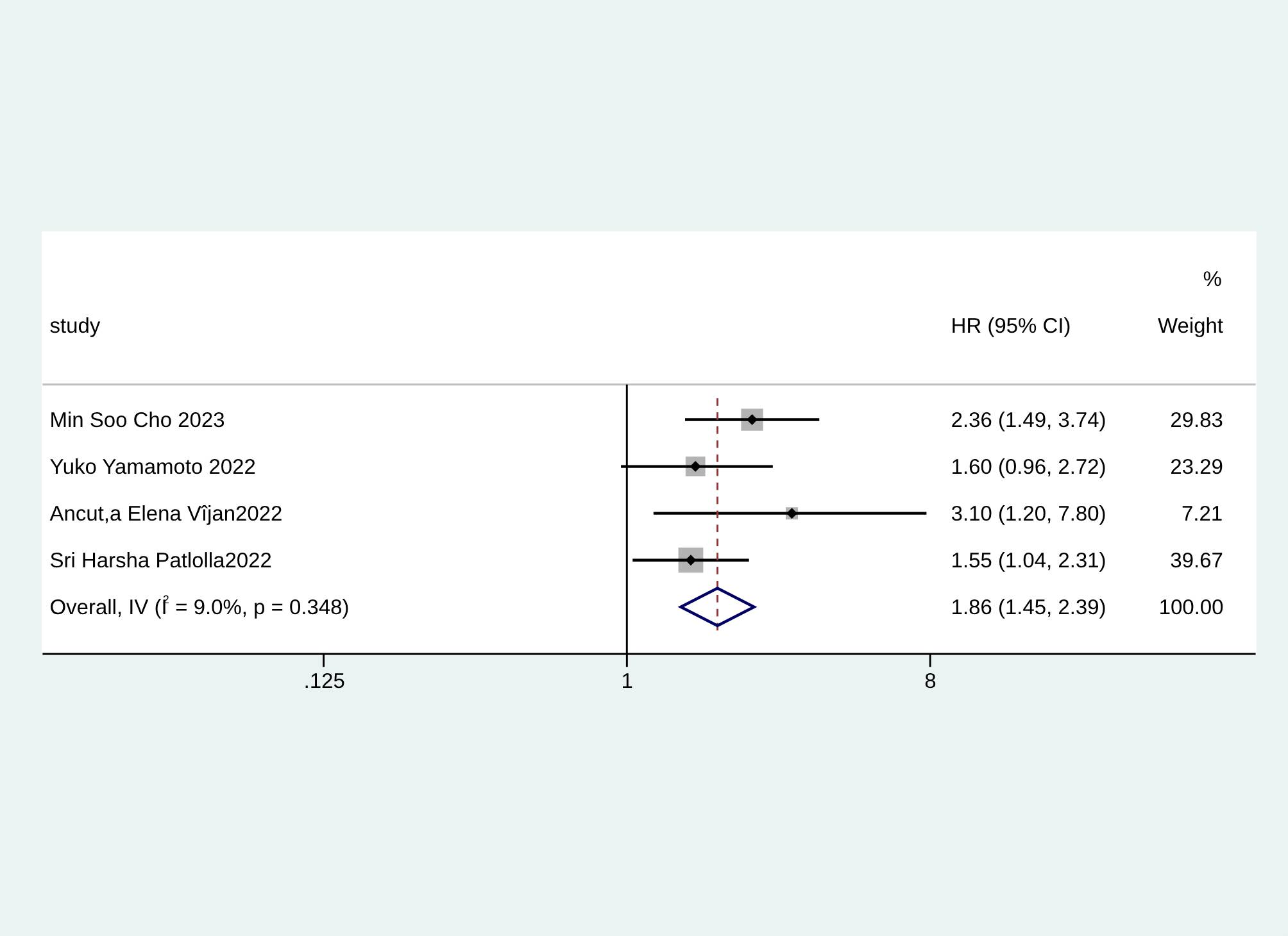
Supplementary Figure 37.**Forest plot for HF.

**
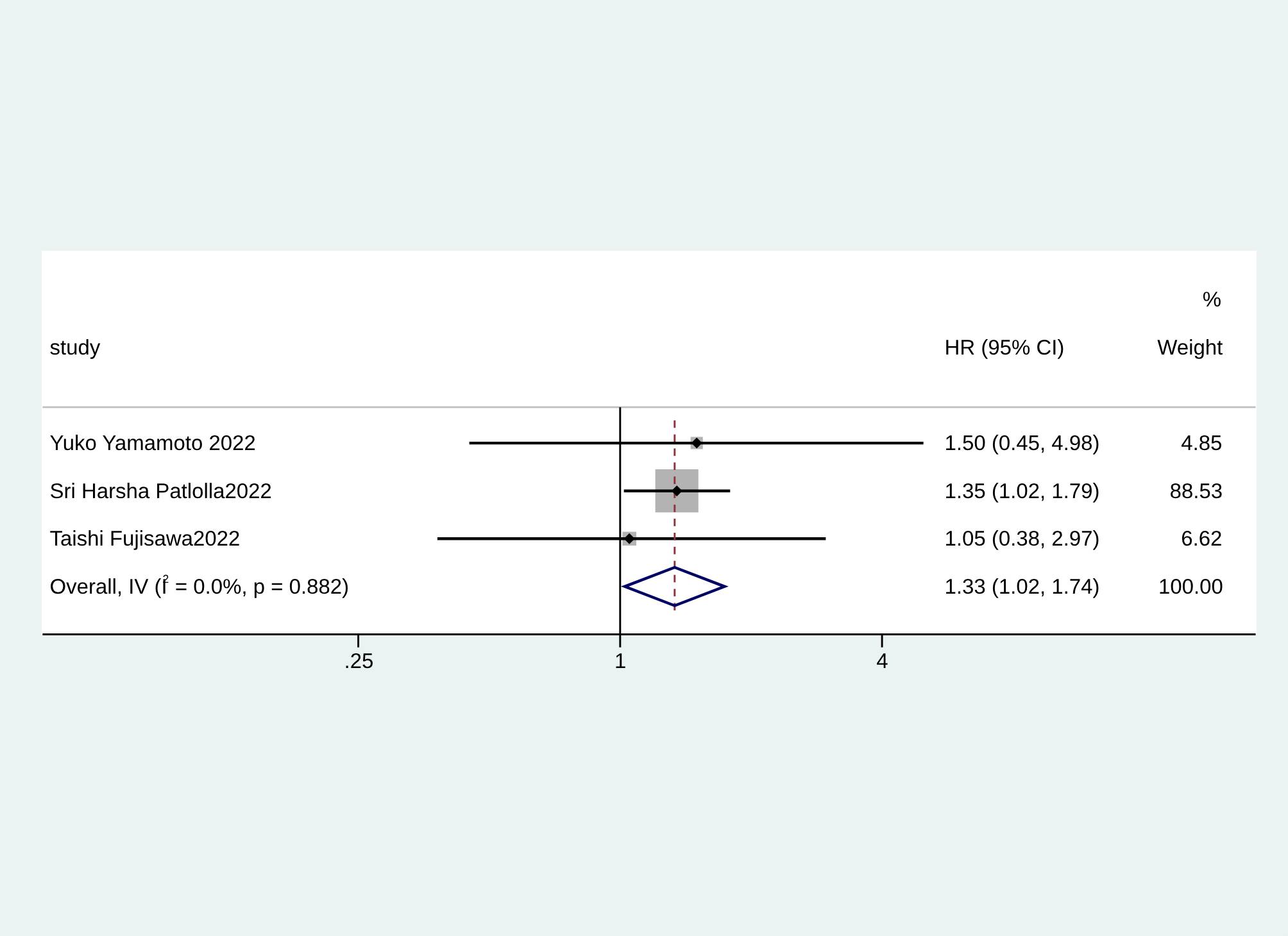
Supplementary Figure 38.**Forest plot for CLD.

**
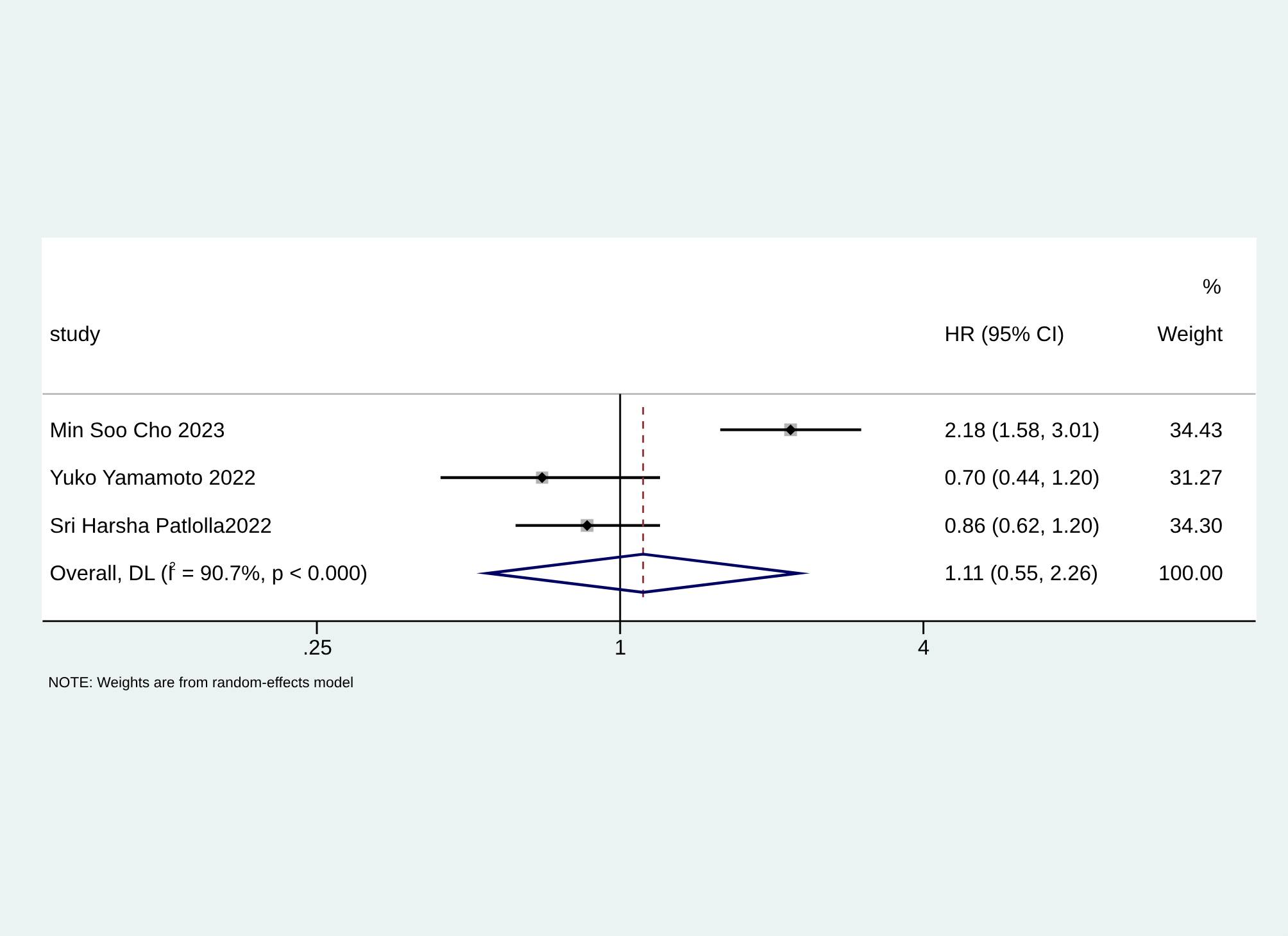
Supplementary Figure 39.**Forest plot for CKD.

**
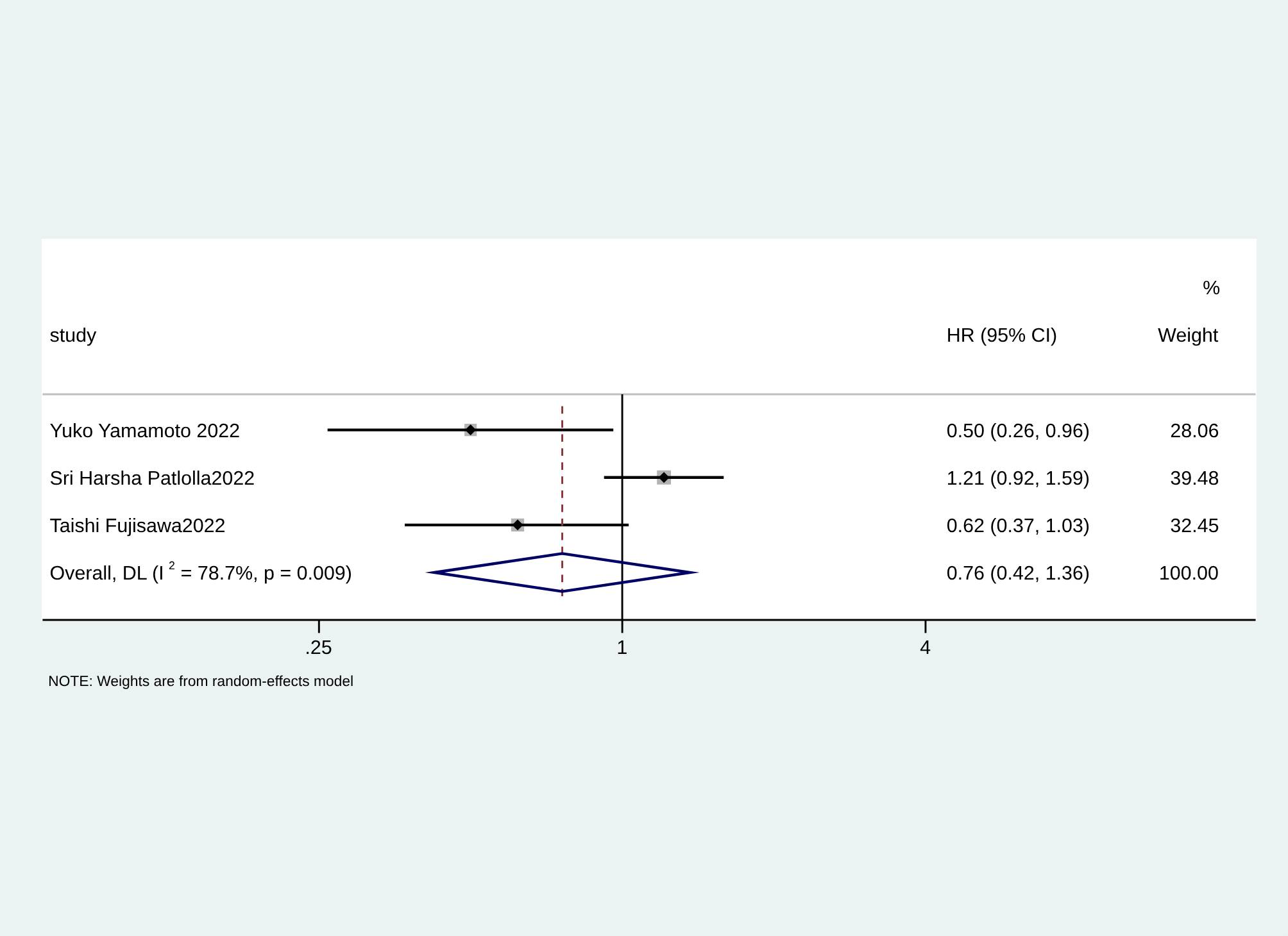
Supplementary Figure 40.**Forest plot for DM.

**
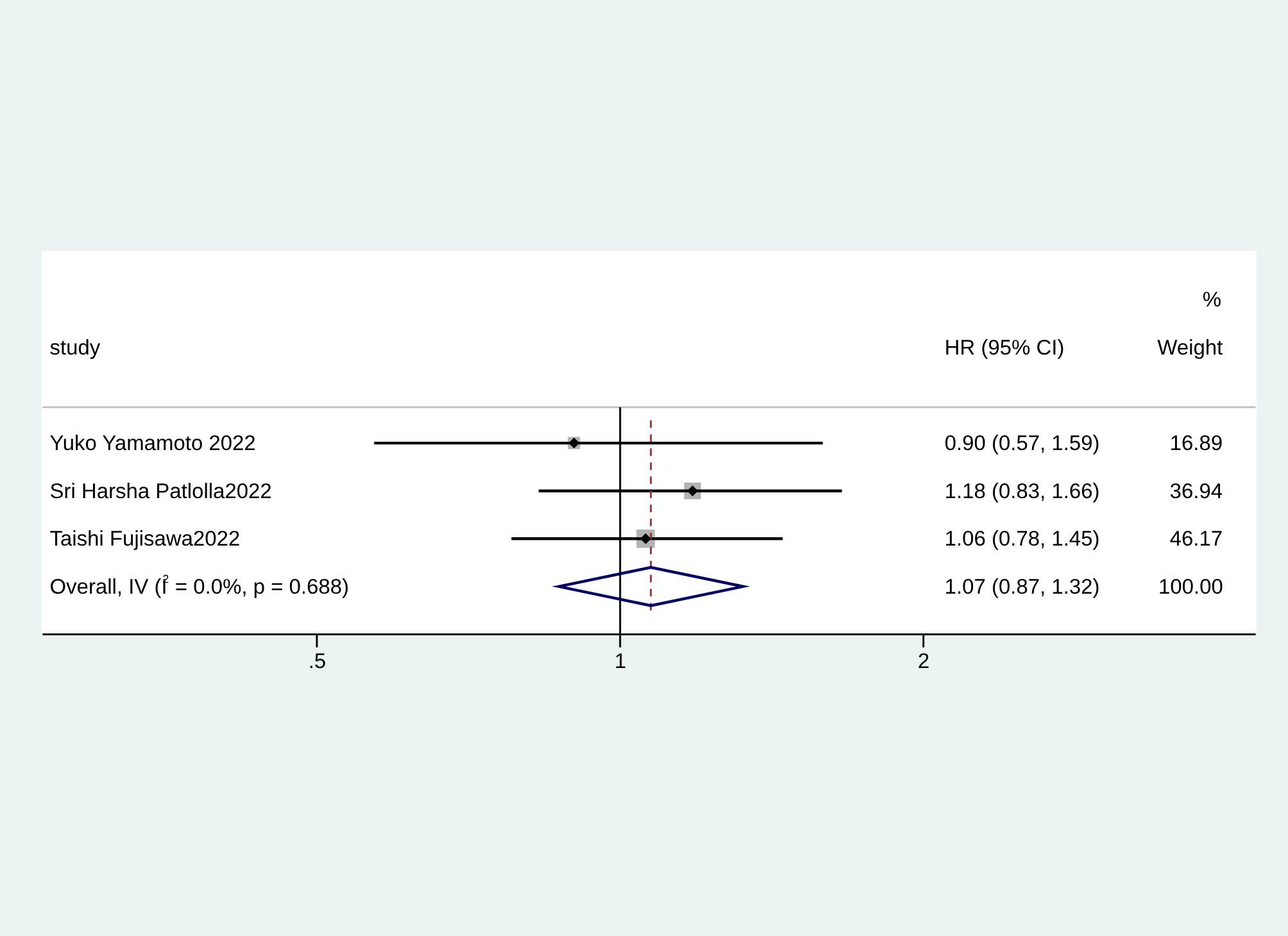
Supplementary Figure 41.**Forest plot for hypertension.

**
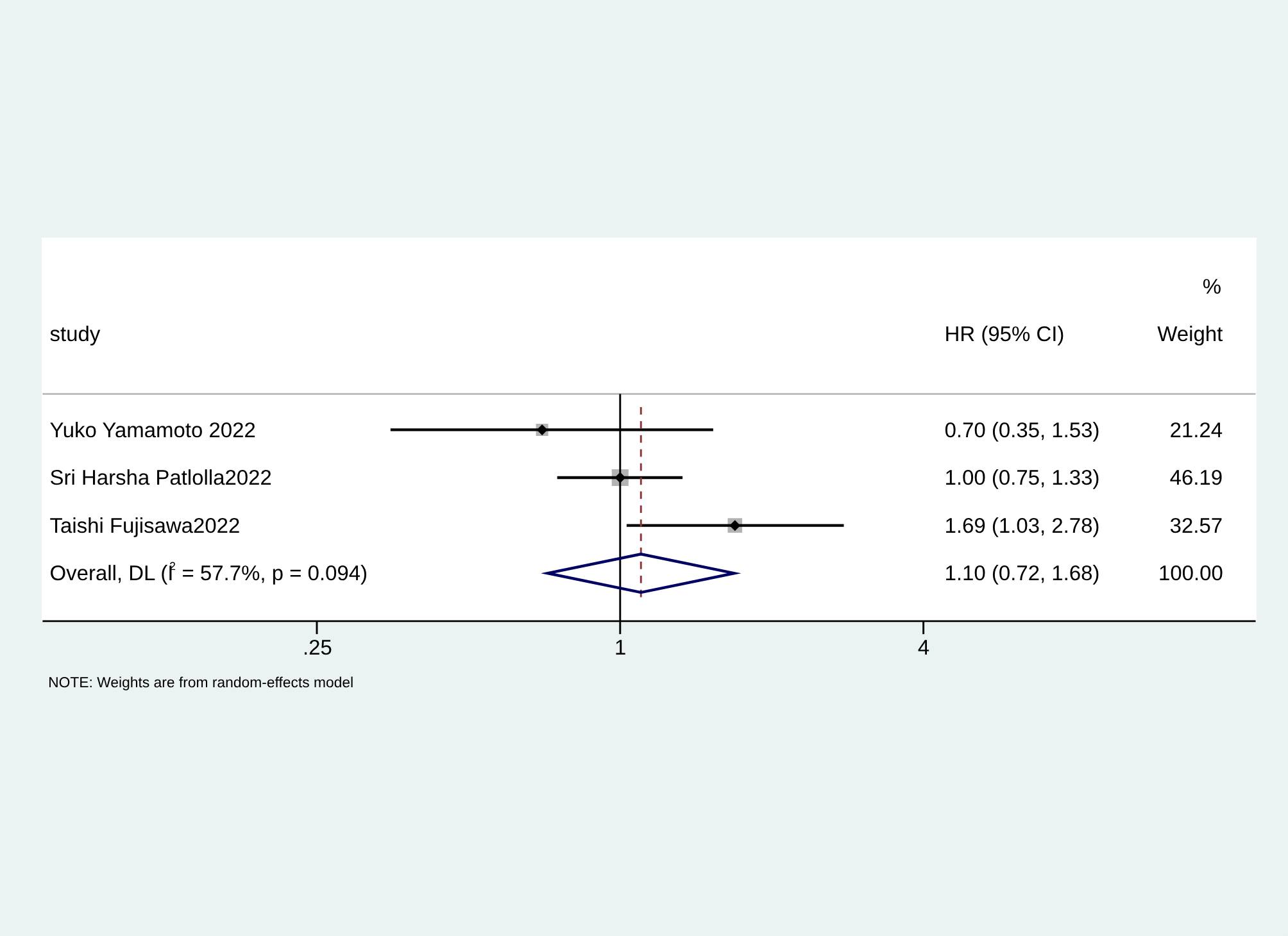
Supplementary Figure 42.**Forest plot for CAD.
