# Supplementary material for: Predictors of significant tricuspid regurgitation in atrial fibrillation: a meta-analysis
Source: Front Cardiovasc Med. 2025 Mar 6;12:1428964. doi: 10.3389/fcvm.2025.1428964 (PMC11922934; doi:10.3389/fcvm.2025.1428964)
Supplement: Supplementary file 1 [file Table1.docx]

Dear Editor，

Thank you for your kind letter of "**Predictors of Significant Tricuspid Regurgitation in Atrial Fibrillation: a meta-analysis**" comments, and carefully proof-read the manuscript to minimize typographical, grammatical, and bibliographical errors.

Here below is our description on revision according to the reviewers' comments.

**Reviewer comments：**

1-L121: Kindly keep the terms consistent.. i.e., Preexisting comorbidities

Response: Thank you for the correction; it has been incorporated into the full text.

2-L159: Random effects.

Response: Thank you for the correction; it has been updated.

3-L207-208: Kindly change punctuation for clarity (eg., SMD =0.18; 95% CI: 0.01,0.36; I2 = 0%; p=0.440).

Response: Thank you for your suggestion; we have updated the format of the full text data report.

4- L265: Consistent with existing literature..

Response: Thanks for your suggestion, we have updated this statement to 'Consistent with existing literature'.

5-L322-323: unclear what is being conveyed here.

Response: Thank you for your correction, we have rewritten the original sentence as‘Given the limitations of the current stage of the study, the original data only support a broad analysis of the relationship between various influencing factors and the occurrence of significant TR in patients with AF.’

6-L338: including / -

Response: Thank you for your suggestion. In order to ensure the fluency of the presentation, we have updated the presentation of the conclusion section. The updated text reads as follows:

*Clinical characteristics, including age, age ≥ 65 years), female sex, echocardiographic parameters (including LVEF, RV FAC, RVSP, and SPAP), and prior comorbidities (including PAF, HF, and CLD) may serve as potential predictors of significant TR. Further studies are needed to determine the long-term prognostic values of the above factors in significant TR, and more attention should be paid to looking for superior predictors of this aspect in the future.*
